# Community‐Based High‐Intensity Multimodal Training: A Mixed‐Method Evaluation of a Randomised Control Trial

**DOI:** 10.1002/ejsc.70211

**Published:** 2026-06-26

**Authors:** Tijana Sharp, Lee Wallace, Samuel Higham, Charlize D. Marques, Aaron J. Coutts, Nathan Mageropolous, Andrew Novak, Mark Brewer, Katie Slattery

**Affiliations:** ^1^ Faculty of Health School of Human Performance Rehabilitation and Population Health University of Technology Sydney Sydney Moore Park Australia; ^2^ Faculty of Health Human Performance Research Centre University of Technology Sydney Sydney Moore Park Australia; ^3^ Performance Everyday Sydney Neutral Bay Australia

**Keywords:** exercise, fitness, high intensity, preventative health, recreational, training

## Abstract

This study evaluated the feasibility, acceptability and perceptual outcomes of a 7‐week High‐Intensity Multimodal Training (HIMT) intervention and an active matched control (Inter‐Session Concurrent Training [ISCT]) in recreationally active adults across three community sites. This study highlights challenges of conducting experimental research in applied settings in collaboration with industry partners and provides recommendations for HIMT research and practice. This study used a mixed‐methods convergent design to evaluate a two‐arm parallel randomised controlled trial in recreationally active adults. The HIMT group completed 4 × 45‐min sessions/week combining aerobic and resistance exercise in a circuit format. The ISCT group completed 2 × 45‐min resistance circuit sessions/week and 2 × 45‐min aerobic circuit sessions/week. Data were collected at baseline (Week 0), post‐intervention (Week 7), and 4 weeks follow‐up (Week 11) across three trial sites between June 2024 and November 2024. 80 participants (49 females, 31 males) completed baseline testing (138% of target). One participant withdrew post‐baseline, with 79 randomised to HIMT (*n* = 41) or ISCT (*n* = 38). Post‐testing was completed by 75 participants and follow‐up by 73 participants. Session attendance was 91.4%, with participants completing 3.52 ± 0.52 sessions/week. Mean session rating of perceived exertion was 7.47 ± 1.40 (CR‐10). The programme was perceived as tolerable and enjoyable. HIMT and ISCT for recreationally active adults within community‐based settings is feasible and acceptable. Exercise professionals may consider using HIMT and ISCT to engage recreationally active adults in structured exercise, promoting preventative health and positive lifestyle behaviours.

**Trial Registration:**

This Randomised Controlled Trial was pre‐registered with the Australian and New Zealand Clinical Trial Registry: ACTRN12624000556549, Protocol: https://osf.io/mxy93/, ETH24‐9364

## Highlights

- Seven weeks of HIMT and ISCT was feasible and acceptable in recreationally healthy adults, with adherence and attendance exceeding expectations, though this may partly reflect the supportive research context.
- Positive perceptual outcomes were observed across both groups, with qualitative responses indicating the intervention was suitable and enjoyable, despite low to moderate quantitative scores of enjoyment, self‐efficacy, and behavioural regulation.
- A mixed methods approach revealed divergence between quantitative and qualitative findings, underscoring its value in exercise intervention evaluation.
- The intervention was co‐designed with community practitioners, supporting ecological validity, and was conducted and reported in accordance with open science and relevant reporting guidelines.

## 1 Introduction

Consistent participation in physical activity is known to improve physical and psychological health, well‐being and quality of life (Warburton and Bredin 2017; Penedo and Dahn 2005). Current guidelines recommend that healthy adults participate in both aerobic (i.e., ≥ 30 min of moderate intensity on 5 days/week or ≥ 20 min of vigorous intensity on 2 days/week) and resistance‐based exercise (i.e., ≥ 2 days/week) to reduce the risk of morbidity and mortality (American College of Sports Medicine 2025). However, adherence to these guidelines remains low, with lack of time and poor enjoyment or motivation among the most frequently reported barriers to participation (Ekkekakis et al. 2008; Ryan and Deci 2000). Combining high‐intensity aerobic and resistance training modalities into a single time‐efficient training session (e.g., group fitness, CrossFit, Hyrox) may assist individuals in achieving physical activity guidelines compared to accumulating separate aerobic and resistance exercise sessions. Recently, the term high‐intensity multimodal training (HIMT) has been introduced to capture all styles of combined aerobic, resistance and/or bodyweight training performed at a high or vigorous intensity (T. Sharp et al. 2022).

Previous studies have demonstrated positive effects of HIMT on health and performance outcomes when examined in isolation or compared to a control group (e.g., sedentary, no intervention, habitual activity) (Ballesta‐García et al. 2019; Batrakoulis et al. 2018; McRae et al. 2012; Sperlich et al. 2017). However, the magnitude of these effects in HIMT remains unclear when compared to other methods of combined aerobic and resistance training (i.e., inter‐session concurrent training [ISCT]). This may be due to a lack of standardisation in the prescription and reporting of training variables in HIMT (e.g., exercise selection, equipment, order, volume, intensity) that limit the ability to synthesise and compare previous findings (T. Sharp et al. 2024). Similar poor rigour has been demonstrated in the reporting of non‐training variables that relate to intervention development or delivery (e.g., training programme prescription, interventionist qualification, physical environment, music selection, participant support and resources) (T. Sharp et al. 2024). While previous studies have commonly reported methodologies in accordance with guidelines such as the Consolidated Standards of Reporting Trials (CONSORT) or Standard Protocol Items: Recommendations for Interventional Trials (SPIRIT) checklists, exercise intervention specific guidelines are not consistently implemented (e.g., Consensus on Exercise Reporting Template [CERT]) resulting in the loss of key details when reporting HIMT interventions (e.g., replicable exercise selection and intensity) (T. Sharp et al. 2024; Slade et al. 2016; Hopewell et al. 2025; Chan et al. 2013). Such disparities reduce the replicability of HIMT research, further limiting comparisons between other modes of combined training (i.e., matched training dose). The use of more rigorous, open science practices may promote greater internal validity of select studies (e.g., laboratory based), at the possible expense of ecological validity among other studies (e.g., community, workplace interventions).

To date, few HIMT interventions have emphasised an ecologically valid approach in research implementation (e.g., school or work‐based interventions) (T. Sharp et al. 2024; Eather et al. 2020; Kennedy et al. 2020). Given the current popularity of HIMT in the community, conducting research at an implementation level (i.e., within community‐based settings) in collaboration with practitioners is an integral step in the integration of research into practice (Bishop 2008). A clearer understanding of the feasibility, acceptability and perceptual outcomes of applied methods of HIMT prescription and delivery may promote greater tailored service delivery in the community and foster sustainable long‐term physical activity behaviours. Therefore, the purpose of this study is to evaluate the feasibility, acceptability and perceptual outcomes of a 7‐week HIMT and ISCT intervention in apparently healthy adults across three community locations using a mixed methods evaluation randomised control trial design. This study also aims to present perspectives of participants, practitioners and researchers to provide insight into the methods used to promote reporting transparency and rigour while optimising internal and ecological validity. Additionally, this study aims to reflect on the challenges associated with conducting experimental research in community settings involving practitioners. Finally, this article aims to provide recommendations for future researchers and practitioners conducting research or working in HIMT.

## 2 Methods

### 2.1 Study Design, Participants, Setting

**FIGURE 1:**
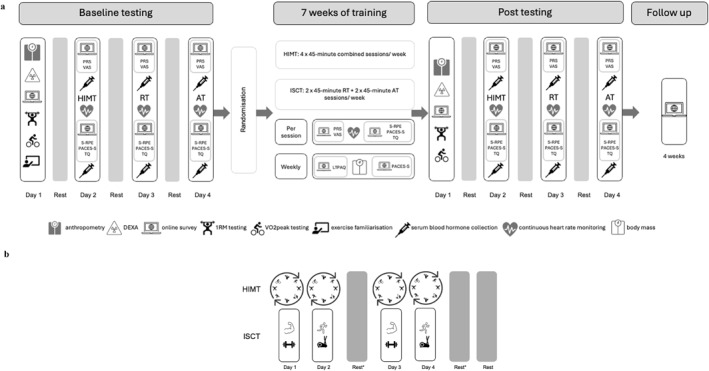
(a) Outline of the Study Procedures. (b) Weekly Training Format. AT, aerobic training, HIMT, High‐Intensity Multimodal Training, ISCT, Inter‐Session Concurrent Training, LTPAQ, Leisure Time Physical Activity Scale, PACES‐S, shortened version of the Physical Activity Enjoyment Scale, PRS, Perceived Recovery Scale, RT, resistance training, S‐RPE, Session Rating of Perceived Exertion, TQ, Training Quality, VAS, Visual Analogue Scale.

This study protocol was pre‐registered with the Australian New Zealand Clinical Trials Registry (ANZCTR: 12624000556549) and prepared in accordance with the CERT (Slade et al. 2016), CONSORT (Hopewell et al. 2025), A Methodological Checklist for Studies of Pleasure and Enjoyment Responses to High‐Intensity Interval Training and Guideline for Reporting for Intervention Development Studies (GUIDED) checklist (Ekkekakis et al. 2023a, 2023b; Duncan et al. 2020) (see Supporting Information [Link], [Link], [Link], [Link]). This study received ethics approval from the University Human Research Ethics Committee (HREC ID: ETH24‐9364), with all participants providing written informed consent. A two‐arm parallel randomised control trail design was used. Complete methods are available on the Open Science Framework (OSF) (osf.io/mxy93). Interventions spanned 7 weeks, with data collection occurring at baseline (T1), post‐intervention (T2) and at 4 weeks follow up (T3) (Figure 1a). Following baseline testing, participants were stratified by sex (i.e., male, female) and individually randomised at a ratio of 1:1. An independent third party generated a series of randomised numbers via a computerised random number generator, and another third party allocated matched participant codes according to the random number sequence. The study ran between June 2024 and November 2024 and was delivered across three indoor gym‐based locations in collaboration with two external HIMT service providers. Recruitment was achieved through flyer distribution (available OSF) via email (i.e., local workplaces and organisations who voluntarily distributed), social media, and local advertising across the three locations. All participants provided written informed consent to participate.

*A priori* sample size calculation was performed using G*Power software (Version 3.1). Power calculations were based on the primary outcome of muscular strength (*α* = 0.05, power = 0.80, effect size 0.42 (Cohen's f) (Currier et al. 2023). The effect size (0.42) has been observed in previous research comparing the effects of resistance training protocols (low external load) for muscular strength (Currier et al. 2023). Sample size was calculated to be *n* = 47, with *n* = 24 per group to detect a small‐to‐medium effect size for the primary outcome measure (muscular strength) (Currier et al. 2023). Assuming a drop‐out rate of ∼20%, 29 participants per group was estimated (totalling *n* = 58) (Collins et al. 2022). To be eligible, participants were aged 18–65 years, recreationally active and considered non‐clinical (see Table 1 for a detailed description of the study eligibility criteria).

**TABLE 1:** Eligibility criteria.

Inclusion criteria	Apparently healthy, recreationally active adults that had been participating in at least 30 min of moderate or 20 min of vigorous activity on at least 3 days per week for the last 6 months. At least 1 of these training sessions must have been resistance training (e.g., lifting weights, bodyweight training). This was to ensure participants are accustomed to both aerobic and resistance exercise stimuli (at a level reaching physical activity guidelines) to account for the possible confounding effects of training status. 6 months was chosen as the minimum participation duration to be currently recreationally active as this reflects a time period where individuals have participated long enough to experience possible health benefits. Aged 18–65 years. Male and female. Willingness to refrain from participating in additional forms of high‐intensity activity or resistance exercise during the study (e.g., competitive or social sport, other group training, other high‐intensity aerobic or resistance exercise), Willingness to give written informed consent and willingness to participate to and comply with the study.
Exclusion criteria	Clinical populations (i.e., individuals requiring assessment and clearance to exercise by a health professional such as a general practitioner or exercise physiologist due to their condition or pathology). Individuals with metabolic or chronic disease, musculoskeletal injuries. Individuals undergoing pharmacological treatment for psychological disorders. Pregnant women. Anti‐inflammatory and/or pharmacological treatment as this may have interfered with inflammatory and metabolic blood markers and exercise adaptations. Exclusion based on these treatments was completed on a case‐by‐case basis. Treatments were identified in the health pre‐screening and participants were excluded if there is evidence linking the treatment to changes in the physical and mental health markers were measured in this study. Contraindications to exercise as identified in the health pre‐screening (Exercise and Sports Science Australia, Adult Pre‐Exercise Screening tool) (ESSA APSS) (Exercise and Sports Science Australia 2019).

### 2.2 Intervention

#### 2.2.1 Training Programs

Following baseline testing (i.e., 4 testing sessions), participants were stratified by sex and randomly allocated into either the HIMT group or ISCT group at a 1:1 ratio (Figure 1a). Both groups trained for 45 min, 4 days per week for 7 weeks (i.e., total of 28 training sessions) (Figure 1a). A 7‐week training period was prescribed to ensure pre and post data were collected at a similar time period of the menstrual cycle for female participants and to align with prior interventions observing strength adaptations following 6–8 weeks of HIMT (Bahremand et al. 2020; Buckley et al. 2015; Posnakidis et al. 2022). The HIMT group completed 4 HIMT sessions per week (combined high‐intensity aerobic and resistance/bodyweight exercise within a single session), while the ISCT group completed 2 aerobic and 2 resistance sessions per week in alternating order (Figure 1b). This frequency allowed participants to meet physical activity guidelines across both training modes. Participants were prescribed a rest day after every two consecutive training days to support recovery.

Training programs were prescribed in collaboration with qualified HIMT practitioners (qualifications described below) with experience in delivering group exercise for healthy adults to enhance ecological validity. Exercise intensity for aerobic and resistance exercises in the HIMT programme was prescribed as ≥ 7 rating of perceived exertion (RPE) (CR‐10) and was monitored during HIMT session delivery using RPE (i.e., RPE ≥ 7) and/or repetitions in reserve (RIR) (i.e., ≤ 3 RIR) to reduce inter‐individual variability in the interpretation of RPE (i.e., RIR may provide a slightly more objective task specific metric) (Bishop et al. 2025; Lovegrove et al. 2022). RPE and RIR were anchored with participants in familiarisation sessions. Session RPE (S‐RPE) (CR‐10) was monitored following each session as a global measure of training session intensity and a measure of programme feasibility (Foster et al. 2001). All resistance‐based sets in the HIMT programme were prescribed as 10 repetitions allowing standardisation of prescribed volume‐load between groups and alignment with repetition ranges associated with peripheral strength adaptations (American College of Sports Medicine 2025; Docherty and Sporer 2000). HIMT sessions (exercise selection, work:relief ratios) varied every 2 weeks to maintain variety and apply progressive overload. The ISCT programme was matched to HIMT for weekly exercise selection, prescribed intensity (≥ 7 RPE), and volume (interval duration, work:rest, sets, reps). Thus, the only prescriptive difference between groups was exercise mode format (i.e., combined vs. separate aerobic and resistance sessions) (Figure 1b).

Progressive overload was applied to the interventions by manipulating prescribed training volume (i.e., increasing work period duration, net‐work duration, reducing work to relief ratio) and/or exercise complexity every 2 weeks. Exercise prescription terminology (e.g., work period, relief period) was defined in accordance with previous high‐intensity interval training (HIIT) literature (Buchheit and Laursen 2013a, 2013b). Work‐to‐relief intervals progressed from 40 s work: 20 s relief to 45 s work: 15 s relief over the 7‐week intervention (Table 2). Relief periods referred to active transitions between exercises within a series, while a series consisted of one full round of exercises (e.g., eight exercises before a recovery period). Between‐series recovery referred to passive rest (Buchheit and Laursen 2013b). This progression was designed to minimise post‐exercise soreness, fatigue, and musculoskeletal injury risk. Training volume was prescribed using time (e.g., 45 s work to 15 s rest).

**TABLE 2:** Prescribed dose of HIMT and ISCT training programs.

Week	T1	1	2	3	4	5	6	7	T2
Total training session time (min)	45	45	45	45	45	45	45	45	45
Total working session time (min)	33	33	33	30	30	30	30	33	33
Total series time (min)	24	24	24	24	24	24	24	24	24
Series (n)	3	3	3	2	2	2	2	3	3
Series duration (min)	8	8	8	12	12	12	12	8	8
Work period (s)	40	40	40	60	60	30	30	45	40
Relief period (s)	20	20	20	30	30	15	15	15	20
Repetitions/set	10	10	10	10	10	10	10	10	10
Between series recovery (s)	180	180	180	180	180	180	180	180	180
Work time (min)	16	16	16	16	16	16	16	18	16
Relief time (min)	17	17	17	14	14	14	14	15	17
W:R (intra‐series)	2:1	2:1	2:1	2:1	2:1	2:1	2:1	3:1	2:1
Sessions/week	3	4	4	4	4	4	4	4	3
Repetitions/week	360	480	480	320	320	640	640	240	360
Intensity (RPE)	≥ 7	≥ 7	≥ 7	≥ 7	≥ 7	≥ 7	≥ 7	≥ 7	≥ 7
Other progression methods	n/a	Repeat of familiarisation exercises with added session	Repeat of week 1	Exercise complexity	Repeat of week 3	Exercise complexity	Repeat of week 5	Exercise complexity, reduction in W:R	n/a

Abbreviations: n/a, not applicable; *n*, number; min, minutes; RPE, Rating of Perceived Exertion; s, seconds; T1, baseline testing; T2, post‐testing; W:R, work to relief ratio.

Both HIMT and ISCT sessions began with a 5‐min warm‐up circuit of the session's prescribed exercises performed at RPE 3 (“moderate”). Instructors provided a group overview of the session format, including exercise selection, order, target volume (determined during baseline familiarisation), and prescribed intensity. Instructors demonstrated each exercise, explained the circuit structure, and provided technique cues as required. Sessions concluded with a 5‐min instructor‐led cool‐down featuring static stretching of major muscle groups. Sessions were offered in the morning (∼7:00–9:00 am), midday (∼12:00–1:00 pm), and evening (∼5:30–7:30 pm) across all indoor gym‐based locations to support scheduling flexibility. Training occurred in small groups (three to eight participants per instructor). When more than eight participants were scheduled, an additional interventionist assisted with session delivery. Participants were asked to refrain from additional high‐intensity aerobic or resistance exercise or competitive or recreational sport during the study. Compliance was monitored via a weekly self‐report survey (Qualtrics XM Platform 2024; Provo, Utah) (Godin 2011). Complete details of prescription considerations and training programs are available in Supporting Information [Link], [Link]. All familiarisation and training sessions took place at participants' preferred location, with each site equipped identically (free weights, ergometers, heart‐rate monitors) to ensure standardisation.

#### 2.2.2 Participant Non‐Adherence

Exercise programs were designed using traditional progressive overload principles to support safety and adaptation (American College of Sports Medicine 2025). Participants were educated on these principles and encouraged to increase load if they exceeded the prescribed repetitions or reduce it if they could not meet the target range. User‐friendly terminology (“combined” [HIMT] and “separate” [ISCT]) was used in participant‐facing materials. Weekly reminders were sent via text or email to assist scheduling, with optional 24‐h booking reminders. Participants could flexibly self‐select or reschedule their training location and time. Adherence was further supported through access to high‐level physical fitness and body composition tests (maximal strength, aerobic fitness, and DXA). SAAFE principles (Supportive, Active, Autonomous, Fair, Enjoyable) guided session delivery to promote engagement, motivation, and enjoyment (Eather et al. 2020; Lubans et al. 2017). Instructors provided verbal encouragement, previously shown to enhance self‐efficacy and autonomous motivation in HIMT (Eather et al. 2020; Lubans et al. 2017). Participants received the upcoming week's programme in advance for familiarisation and had access to a video library of prescribed exercises (https://tinyurl.com/4keemuuv). The primary researcher conducted optional mid‐intervention check‐in calls to gather early feedback on acceptability, perceptual outcomes, feasibility, and improvement suggestions. No other non‐exercise components (e.g., cognitive behavioural therapy) were provided to participants.

#### 2.2.3 Interventionist Recruitment and Training

All training sessions were supervised by qualified professionals (Accredited Exercise Scientists, Personal Trainers with Certificates III/IV [*n* = 7], or trained third‐year Sport and Exercise Science students [*n* = 13]). Each location had a primary interventionist overseeing delivery, and primary interventionists met every 2 weeks to standardise procedures. All interventionists attended a familiarisation session with the primary researcher covering programme design, exercise selection and order, prescribed intensity (≥ 7 RPE), volume, technical execution, and intensity monitoring (≥ 7 RPE or ≤ 3 RIR). The session also trained interventionists in data collection procedures, including pre‐ and post‐session surveys (Qualtrics), heart rate monitor placement and software use (Polar Electro 2024), and training diary completion. Interventionists were instructed to use the exercise video library (https://tinyurl.com/4keemuuv) and were familiarised with equipment and training spaces to ensure safety and consistency. They also were familiarised with standardised verbal encouragement. Initial familiarisation/testing sessions and training sessions in week 1 were supervised by the primary researcher, after which trained instructors delivered sessions under supervision, ensuring a consistent psychosocial environment (SAAFE principles). Instructors encouraged participants to adjust loads as needed. All outcome testing was conducted by qualified exercise professionals trained together to minimise inter‐ and intra‐tester variability.

### 2.3 Study Outcomes

Intervention evaluation was conducted across five domains as per a previous framework and guiding questions (Bowen et al. 2009; Orsmond and Cohn 2015; Teresi et al. 2022). At T1, standard demographic information (e.g., age, sex) were collected from participants using an online survey via Qualtrics. All other assessments were conducted across 4 non‐consecutive days at T1 (July 2024 to August 2024) and T2 (September 2024 to October 2024). Complete testing protocols have been outlined previously (OSF).

#### 2.3.1 Intervention and Training Session Implementation

Recruitment and retention of participants and interventionists were used to assess implementation. Training session implementation was assessed using S‐RPE, HR data collected from polar software (FT7, Polar Electro, Kempele, Finland) (i.e., session intensity) and the number of sessions completed (i.e., training dose achieved). S‐RPE was collected via a post‐session QR‐code survey (Qualtrics 2024) completed on participants' own devices before leaving each session (Castagna et al. 2017). This survey also recorded the HR monitor number worn. HR was continuously monitored during training using the Polar Club app (Polar Electro 2024) on an iPad, with participants able to view HR in real time (not used for prescribing intensity). After each session, interventionists saved and uploaded the Polar Club file, and individual HR files were downloaded from the web‐based software. All files were processed using customised *R* code (imputeTS package, RStudio v4.3.1). Files were classified as a full session (< 5% missing of 45 min), partial session (< 10% of 45 min), or missing (no HR data). Files with > 5% missing data or an average HR < 60 bpm were excluded. For each session type (HIMT, AT, RT), average HR, HR_max_, time > 77% HR_max_, and proportion of work time > 77% HR_max_ were calculated. The > 77% HR_max_ threshold reflects high‐intensity exercise per ACSM guidelines. HR_max_ was defined as the highest value recorded during pre‐ or post‐intervention $\dot{V}O_{2}$_peak_ testing. Attendance was tracked daily (Microsoft Excel, 2024).

#### 2.3.2 Adaptation and Practicality

Adaptation was determined by modifications prescribed during the training intervention. All modifications including changes to exercise selection, intensity, or volume were tracked daily (Microsoft Excel, 2024) and recorded by the head interventionist at a location or communicated to the primary researcher. If modifications were related to an injury or adverse event, the primary researcher followed up with the trainer and participant within 24 h in line with trial protocols. Practicality was assessed via any adverse events or injuries, which were also recorded by the head interventionist or reported to the primary researcher.

#### 2.3.3 Acceptability and Perceptual Outcomes

Acceptability and perceptual outcomes were assessed through several participant measures. Self‐reported physical activity levels, exercise enjoyment, self‐efficacy for exercise and behavioural regulation towards exercise were assessed at T1, T2 and T3 using the Godin Leisure‐Time Physical Activity Questionnaire (LTPAQ), shortened version of the Physical Activity Enjoyment Scale (PACES‐S), Self‐Efficacy for Exercise Scale (SEE) and Behavioural Regulation in Exercise Questionnaire second edition (BREQ‐2) via an online survey (Qualtrics) (Godin 2011; Chen et al. 2021; Resnick and Jenkins 2000; Markland and Tobin 2004). These instruments demonstrate acceptable test‐retest reliability, convergent and criterion validity within adults (Godin 2011; Chen et al. 2021; Markland and Tobin 2004; Fritsch et al. 2022). Surveys also included open‐ended questions enquiring about what participants enjoyed the most and least about their physical activity mode before, during and after the intervention. Responses at T2 were used to analyse acceptability and perceptual outcomes of the intervention only. Additionally, perceived changes in self‐efficacy and motivation for exercise from T2 and T3 were included in analysis of intervention acceptability and perceptual outcomes. Perceived changes in PA were assessed at T3 in the same way. The survey at follow‐up also included an optional platform for participants to provide open‐ended feedback in response to the questions *“Do you have any other feedback for the research team on the training sessions you completed during this study?”* and *“Do you have any other feedback for the research team on your general experiences during this study?”*. The complete survey instruments and background information are available on The OSF.

### 2.4 Analysis

**FIGURE 2:**
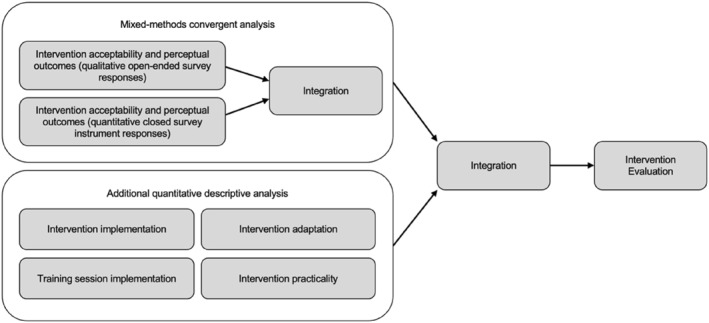
Study analysis flow diagram.

This intervention was evaluated using a mixed methods design. A pragmatic approach enabled the integration of quantitative and qualitative data. This recognises that while reality exists independently of individual perspectives, it is also interpreted through each individual's subjective experience (Morgan 2014). This approach supported methodological flexibility and emphasised practical problem‐solving over philosophical alignment (Creswell and Clark 2017). Quantitative findings were prioritised to evaluate intervention feasibility, acceptability and perceptual outcomes, with qualitative insights used to enrich interpretation and provide additional contextual understanding. Data for intervention and training session implementation, adaptation and practicality were analysed using descriptive statistics (i.e., mean, standard deviation, percentages) or described in a qualitative manner where appropriate. Feasibility, acceptability and perceptual outcomes were analysed using a convergent mixed methods design (Figure 2).

An intention to treat analysis using a linear mixed effects regression (maximum likelihood estimation) was conducted to examine change over time and group × time interactions (2 x 3) for Godin LTPAQ, PACES‐S, SEE and BREQ‐2 scores (Godin 2011; Chen et al. 2021; Resnick and Jenkins 2000; Markland and Tobin 2004). For each outcome measure, a separate regression model was developed with age, sex, baseline, and location specified as fixed effects, the main timepoint:group interaction effect, and random intercepts were specified for each participant ID, given the repeated measures. Sex and age were included as covariates within the model, however, as they were not the primary variables of interest, differences between sexes and ages were not interpreted. To address potential clustering effects of participant training environments, training location was initially included as a random effect, however, given that models did not converge likely due to relatively few locations and additional model complexity, the variable was retained as a fixed effect. Statistical analysis was conducted in *R* (v4.4.3, *R* Core Team, Vienna, Austria) in RStudio (v2024.9.1.394, Posit Team, Boston, MA) via the glmmTMB package (v1.1.11, Brooks et al., 2025). The residuals of each model were inspected via QQ plots and plots of the residuals versus fitted values. Statistical significance was set at *p* < 0.05, however, given that eight different outcome variables were analysed, findings have also been reported in light of a Bonferroni‐corrected value of *p* < 0.006. Ninety‐five percent confidence intervals were used to estimate the precision of effect estimates. Effect sizes for mixed‐effects model contrasts were calculated as standardized mean differences (Cohen's d), with thresholds interpreted as small (0.2), medium (0.5), and large (0.8) (Cohen 1988). The total score for the PACES‐S (mean ± SD) was calculated for participants. Higher scores indicated higher levels of enjoyment (Chen et al. 2021). The self‐efficacy for exercise scale was determined by summing the numerical ratings for each response and dividing by the number of responses (Resnick and Jenkins 2000). BREQ‐2 scores (i.e., amotivation, external regulation, introjected regulation, identified regulation, intrinsic regulation) were calculated by summing the numerical ratings for each sub‐scale (*n* = 5) and dividing by the number of responses (Markland and Tobin 2004). A summary of the outcome measures and respective analysis procedures is available in Table 3.

**TABLE 3:** Summary of outcome measures and analysis methods.

Domain	Outcomes	Measurement tool	Timepoint	Method of analysis
Intervention implementation	Participant recruitment & retention Interventionist and data collector recruitment & retention	Recruitment & retention Recruitment & retention	Intervention duration	Descriptive statistics
Training Session implementation	Training and testing session completion Training session HR Training S‐RPE	Session attendance + reasons for non‐attendance Continuous HR monitoring Post‐training S‐RPE	Every session Every session Every session	Descriptive statistics
Adaptation	Need for modifications	Monitoring spreadsheet (Excel)	Every session	Descriptive statistics
Practicality	Injuries Adverse events Training diaries completed	Monitoring spreadsheet (Excel) Monitoring spreadsheet (Excel) Paper training diaries	Every session Every session Every session	Qualitative description of exercise practicality
Acceptability and perceptual outcomes	Self‐reported physical activity Exercise Enjoyment Self‐efficacy Behavioural regulation for exercise Perceived changes in PA Perceived changes in self‐efficacy and behavioural regulation for exercise Perceptions of exercise enjoyment	Godin LTPAQ (Godin 2011) PACES‐S (Chen et al. 2021) SEE (Resnick and Jenkins 2000) BREQ‐2 (Markland and Tobin 2004) Open‐ended survey responses Open‐ended survey responses Open‐ended survey responses	T1, T2, T3 T1, T2, T3 T1, T2, T3 T1, T2, T3 T3 T2, T3 T2	Mixed methods evaluation with a convergent design (linear mixed models integrated with thematic content analysis in joint displays) (Kondracki et al. 2002; Plano Clark and Ivankova 2016; Jones 2024; Nessle et al. 2023; Guetterman et al. 2015)
	Programme feedback	Open‐ended survey responses	T3	Thematic content analysis (Kondracki et al. 2002)

Abbreviations: BREQ‐2, Behavioural Regulation in Exercise Questionnaire second edition; HR, heart rate; LTPAQ, Leisure Time Physical Activity Questionnaire; PA, physical activity; PACES‐S, Shortened version of the Physical Activity Enjoyment Scale; RPE, Rating Of Perceived Exertion; SEE, Self‐Efficacy for Exercise Scale; T1, baseline testing; T2, post‐testing; T3, 4 weeks follow up.

Open‐ended survey responses relating to perceptual outcomes were analysed using qualitative content analysis previously used in survey research (Kondracki et al. 2002; Smith‐Turchyn et al. 2022). Responses were imported into MAXQDA 2020, and analysis was conducted using a combination of inductive and deductive coding strategies, with themes refined iteratively to reflect common reasons for perceived changes in programme experience. Two authors independently coded all responses, and inter‐rater agreement was calculated. Quantitative data (instrument scores) and qualitative data (open‐ended responses) were integrated using side‐by‐side joint displays, as recommended in mixed‐methods evaluation research, to visually align data sources (Plano Clark and Ivankova 2016; Jones 2024; Nessle et al. 2023; Guetterman et al. 2015). Meta‐inferences were developed to demonstrate integration across datasets (Jones 2024; Nessle et al. 2023; Guetterman et al. 2015). All data were merged following analysis to support overall intervention evaluation (Figure 2).

## 3 Results

### 3.1 Intervention Implementation

**FIGURE 3:**
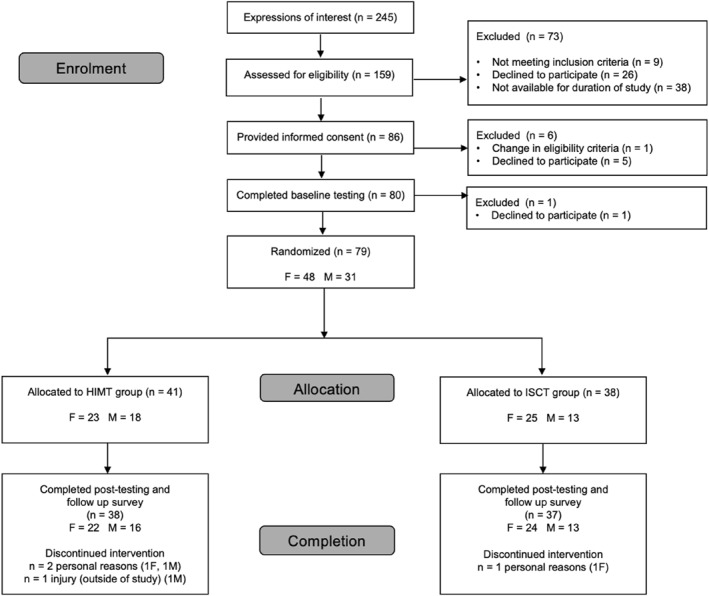
Study Flow Diagram. F, female, HIMT, High‐Intensity Multimodal Training, ISCT, Inter‐Session Concurrent Training, M, male, *n*, number.

Participant flow is shown in Figure 3, and baseline characteristics are presented in Table 4. In accordance with CONSORT guidelines, no statistical tests were performed to compare baseline characteristics (Bland and Altman 2011). Eighty participants completed baseline testing and 79 (48 females, 31 males) were stratified by sex and individually randomised 1:1 to HIMT (*n* = 41; 23 females, 18 males) or ISCT (*n* = 38; 25 females, 13 males). Reasons for not completing the intervention included personal circumstances (*n* = 3) and an injury occurring outside the study (*n* = 1). Seventy‐five participants completed post‐testing, and 73 completed the four‐week follow‐up survey. Seven qualified professionals and 13 undergraduate students were recruited to deliver the intervention, and all were retained. An additional eight volunteers supported data collection at T1 and T2. Results are reported for the 75 participants who completed post‐testing.

**TABLE 4:** Participant baseline characteristics.

Characteristics, mean ± SD	HIMT (*n* = 41)	ISCT (*n* = 38)	Total (*n* = 79)
Age (y)	35.8 ± 11.7	37.0 ± 12.5	36.3 ± 11.9
Female	23	25	48
Male	18	13	31
Body mass (kg)	71.4 ± 11.7	73.2 ± 13.9	72.1 ± 12.7
Height (cm)	169.0 ± 8.9	170.6 ± 8.2	169.7 ± 8.6
BMI (kg/m^2^)	24.9 ± 2.9	25.1 ± 4.4	25.0 ± 3.7
Training location			
1	23	18	41
2	7	10	17
3	11	10	21

Abbreviations: BMI, body mass index; cm, centimetres; HIMT, High‐Intensity Multimodal Training; ISCT, Inter‐Session Concurrent Training; kg, kilograms; m^2^, metres squared, n, number; SD, standard deviation; y, years.

### 3.2 Training Session Implementation

Training session attendance was high (i.e., 63 participants completed > 80% [23 out of 28 sessions], HIMT = 30, ISCT = 33) (P. Sharp et al. 2020; Jankowski et al. 2013) (Table 5). The total number of sessions completed per participant ranged from 13 to 28 out of a possible 28. The average missed session rate was 8.4% (*n* = 2. 3 ± 3.3), with non‐attendance attributed to travel, work, study, illness, injury unrelated to the training, other or non‐reported reasons. Participants completed an average of 3.52 ± 0.5 sessions per week. Thirteen testing sessions (2.89%) were missed due to illness (*n* = 10) or injury outside of the study (*n* = 3). Mean S‐RPE across all training session types was 7.47 ± 1.40, indicating a high‐intensity level of exercise was achieved (American College of Sports Medicine 2025). S‐RPE values for each training session type are presented in Table 6. All recorded S‐RPEs were included and among these 18.57% of S‐RPE reports were below 7.

**TABLE 5:** Training session attendance of a possible 2100 sessions.

Group	Missed training sessions (n)	Missed training sessions (%)
HIMT	113	10.6
ISCT	63	6.1
Total	176	8.4

Abbreviations: HIMT, High‐Intensity Multimodal Training; ISCT, Inter‐Session Concurrent Training; n, number; %, percentage.

**TABLE 6:** Training session intensity.

Training group	HIMT	ISCT		
Session type	HIMT	At	RT	Total
Average HR (bpm)	127.2 ± 14.4	138.3 ± 15.3	123.3 ± 15.7	129.3 ± 16.1
%HR_max_ (%)	74.3 ± 6.2	78.6 ± 5.7	74.7 ± 5.7	75.6 ± 6.2
Time spent > 77%HR_max_ (mins)	18.4 ± 8.1	24.5 ± 8.1	13.2 ± 9.4	18.7 ± 9.5
% work time > 77%HR_max_ (%)	55.6 ± 24.6	74.3 ± 24.6	40.1 ± 28.5	56.7 ± 28.7
RPE	7.4 ± 1.4	8.0 ± 1.2	7.1 ± 1.4	7.5 ± 1.4

*Note:* All values are expressed as mean ± standard deviation.

Abbreviations: %, percentage; AT, aerobic training; bpm, beats per minute; HIMT, High‐Intensity Multimodal Training; HR, heart rate; HR_max_, heart rate maximum; ISCT, Inter‐Session Concurrent Training; mins, minutes; RT, resistance training; RPE, Rating of Perceived Exertion.

Of 2194 available heart rate data files, 2003 were downloaded. Of these, 423 were categorised as complete sessions, 1507 as partial. Seventy‐three sessions (from location 2) were excluded from analysis due to upload error. Therefore, only data from location 1 and 3 was included in the analysis. Among the complete sessions, 31 files were excluded due to > 5% HR data loss (e.g., flat lines) and 1 due to an average HR < 60 beats per minute (bpm). The remaining 390 files (i.e., 19.5% of total downloaded) are included in the descriptive statistics (HIMT = 174, AT = 111, RT = 106) (Table 6). All available HR files were included regardless of participant withdrawal. Only the AT sessions exceeded 77%HR_max_ on average, indicating a high intensity of exercise. The proportion of ‘work’ time per session type spent above 77% was 55.6 ± 24.6% (HIMT), 74.3 ± 24.6% (AT) and 40.1 ± 28.5% (RT) respectively (Table 6).

### 3.3 Adaptation and Practicality

Across the study period, 59 reports of soreness, discomfort, or injury (HIMT = 27, ISCT = 32) were recorded during training sessions by 31 participants. Seven of these resulted in a participant not completing the session (HIMT = 4, ISCT = 3). The remaining 52 events were managed through modifications to the prescribed exercise to allow participants to continue. Modifications included reducing external load (e.g., lowering weight), reducing active range of motion, or simplifying exercise complexity (e.g., unilateral to bilateral stance). No adverse events or injuries occurred during any exercise testing or training sessions.

### 3.4 Acceptability and Perceptual Outcomes

Both the HIMT and ISCT groups significantly increased PA levels from T1 to T2 (HIMT: *b* = 12.331, 95% CI [3.669 to 20.953], *p* = 0.003, *d* = 0.569; ISCT: *b* = 15.047, 95% CI [6.262 to 23.832], *p* < 0.001, *d* = 0.695). The ISCT group also demonstrated a significant decrease in PA levels at T3 compared to T2 (*b* = −13.353, 95% CI [‐22.258 to −4.448], *p* = 0.001, *d* = −0.617) (Table 7). At T2 100% of participants met a ‘sufficient’ (i.e., Godin LTPAQ score > 14) level of activity despite the ISCT group demonstrating a significant reduction in PA from T2 to T3. At T3, only two participants reported scores below 14 (both from the ISCT group). Exercise enjoyment levels were reported as low to moderate across all timepoints in both groups (Table 8). A significant group interaction was observed at T2 and T3 wherein the ISCT group demonstrated an increase in enjoyment scores relative to T1, compared to the HIMT group (T2: *b* = 2.010, 95% CI [0.246 to 3.775], *p* = 0.026, *d =* 0.632; T3: *b* = 1.788, 95% CI [0.011 to 3.566], *p* = 0.049, *d* = 0.562), although no difference was observed between T2‐T3. Self‐efficacy for exercise demonstrated moderate scores in both groups across all timepoints. No significant time or group interactions were demonstrated (*p* > 0.05) (Table 9). Amotivation and extrinsic regulation remained low across groups and time points. Introjected, identified and intrinsic regulation remained moderate across groups and time points. Extrinsic motivation in ISCT significantly decreased from T2 to T3 compared to HIMT (*b* = −0.226, 95% CI [−0.466 to −0.005], *p* = 0.045, *d* = −0.400) (Table 9). No other significant time:group interactions were observed for amotivation, introjected, intrinsic or identified motivation (*p* > 0.05) (Table 9). The complete statistical results are available in Supporting Information S7.

**TABLE 7:** Physical activity levels (Godin LTPAQ scores and open‐ended survey responses).

Group	LTPAQ score	Themes at T3 in both groups	Survey responses at T3 in both groups	Meta‐inferences
HIMT	T1: 41.15 ± 19.84 T2: 55 ± 15.78^a^ T3: 46.68 ± 24.82	‘Routine created consistency’: The intervention created a sense of routine participants could commit to.	*“Was in a good routine during the programme and was easy to maintain.”–3* *“The study allowed me to create a consistent routine, making working out and exercising become more of a habit to me.” –72* *“Programme was a circuit breaker that got me back into regular exercise routine!”–16* *“The study put me into a good daily routine that I have tried to continue each week now.”–58*	Physical activity was likely to increase when the intervention provided a sense of routine.
		‘Motivated to move’: The extrinsic and intrinsic benefits felt during the intervention provided motivation to continue similar PA behaviours.	*“I saw progress in my body, strength and fitness during the study.”–75* *“I feel more confident going to the gym.”–75* *“I feel fitter since the doing the trial, but now I want to lose the weight and maintain the fitness.”–52* *“I'm now more motivated to continue building muscle and becoming more fit.”–70* *“Want to carry on the positive impact after the training.”–11* *“Yes, I am doing more exercise than I was before. I'm more motivated since finishing the study.”–69*	Motivation and confidence were greater when participants were able to notice their improvements (e.g., physical fitness) during the physical activity intervention.
ISCT	T1: 43.76 ± 23.49 T2: 58.84 ± 15.24^b^ T3: 45.96 ± 24.48^a^	‘Already active’: Many participants were already completing similar PA behaviour prior to participation.	*“I was already engaged in physical activity before I started to participate in this project.”–13* *“It was pretty full on before time wise so that's about the same.”–10* *“I used to train similar amounts of time pre the study.”–21*	For those already active, the interventions had less of an impact on their ongoing physical activity levels.
		‘When life gets in the way’: PA behaviours were impacted by life circumstances following the intervention.	*“Less exercise due to life events.”–36* *“Very busy at work. Hard to find time to exercise. Unrelated to the study ending, just a coincidence.”–63* *“I tried to maintain similar activities as I did in the programme. However, due to the limited equipment in the outdoor gym, I could not maintain a completely similar activity.”–79*	For those who were not already active to a similar level of the intervention, maintaining physical activity after the intervention was challenging.

Abbreviations: HIMT, High‐Intensity Multimodal Training; ISCT, Inter‐Session Concurrent Training; T1, baseline testing; T2, post‐testing; T3, 4 weeks follow up.

^a^ *p* < 0.01.

^b^ *p* < 0.001.

**TABLE 8:** Exercise enjoyment (PACES‐S scores and open‐ended survey responses).

Group	PACES‐S score	Themes at T2	Survey responses at T2	Meta‐inferences
HIMT	T1: 12.8 ± 3.64 T2: 11.54 ± 2.99 T3: 11.97 ± 2.86	*What was most* enjoyable about the training?		Although enjoyment scores were modest overall, feelings of achievement and social support contributed meaningfully to participants' positive affect. This disconnect highlights that positive factors such as the group environment and a sense of achievement may have outweighed a dislike for high‐intensity exercise.
		‘Variety and balance’: The variety and balance of resistance and aerobic exercise in HIMT was enjoyable and engaging.	*“Variety of exercise, breaking up the cardio.”–27, “I enjoyed the mix of cardio and weights and the mental engagement going from one station to another.”–31*	
		‘Sense of achievement’: Feeling a physical and/or mental accomplishment, while making progress during in the training was enjoyable.	*“Feeling fitter, the accomplishment.”–76, “It made me feel fitter and stronger and more energised.”– 8*	
		‘Supportive environment’: Training with a consistent supportive coach and group of people was enjoyable.	*“Working out together.”–13, “The comradery was great.”–19* *“Encouragement of the trainers.”–55, “Support from coaches.”–36*	
		*What was least* enjoyable about the training?		Despite similar enjoyment scores, the groups reasons for enjoyment were underpinned by different factors (e.g., structural novelty vs. routine predictability), illustrating that enjoyment can manifest through multiple pathways. Perceptions of exercise intensity influenced enjoyment differently: In HIMT, high effort and low recovery reduced affective responses, while for ISCT, monotony dampened enjoyment. This may explain the trivial difference in enjoyment scores between groups.
		‘No time to recover’: The alternate resistance and aerobic exercise with short rest periods in HIMT sessions was tiring.	*“Not getting to fully recover between movements.”–49, “It was harder to lift weights after doing all the cardio so couldn't lift as heavy.” –75*	
		‘Too intense’: The training (particularly the cardio exercises) was tiring and physically intense, and this was not enjoyable.	*“Exhaustion after cardio exercises.”–48, “Very little rest and cumulative fatigue is a killer.”–25*	
ISCT	T1: 11.39 ± 3.08 T2: 12.38 ± 2.87^#^ T3: 12.56 ± 3.46^#^	*What was most* enjoyable about the training?		
		‘Keeping it consistent’: Focussing on one modality per day, while maintaining variety in a week and throughout the programme was uniquely enjoyable for ISCT participants.	*“I liked being able to focus on strength days, and not have it be compromised by cardio.”–42, “I like separate so that I was only doing one type per day. Could just focus on strength or cardio.”–34*	
		‘Sense of achievement’: Feeling a physical and/or mental accomplishment, while making progress during in the training was enjoyable.	*“Feeling I was getting stronger in the resistance training.”–30, “The mood lift/uplift it provided afterwards.”– 35*	
		‘Supportive environment’: Training with a consistent supportive coach and group of people was enjoyable.	*“The group aspect of it. Having people to motivate with you.”–34, “Meeting the same people training with me every week.”–53, “The helpful coaches.”–7, “They really pushed me in each session to try more and get better.”–77*	
		*What was least* enjoyable about the training?		
		‘Repetitive’: The training felt repetitive at times completing the same exercises in one session.	*Cardio—not enough diversity, only did 4 differences exercises each time.”–60* *“Felt very repetitive at times and was hard to track if I was improving when it changed every 2 weeks.”–34*	
		‘Too intense’: The training (particularly the cardio exercises) was tiring and physically intense, and this was not enjoyable.	*“Feeling super out of breath on the cardio.”–30, “Although it was good to do strength separate to my cardio sessions. I found the cardio sessions hard when I was tired.”–21*	

Abbreviations: HIMT, High‐Intensity Multimodal Training; ISCT, Inter‐Session Concurrent Training; PACES‐S, shortened version of the Physical Activity Enjoyment Scale; T1, baseline testing; T2, post‐testing; T3, 4 weeks follow up.

#Significant between group effect (*p <* 0.05).

**TABLE 9:** Self‐efficacy and behavioural regulation for exercise scores.

Group		HIMT			ISCT		
Timepoint		T1	T2	T3	T1	T2	T3
SEE score		5.98 ± 2.04	5.91 ± 2.06	5.89 ± 2.20	6.11 ± 1.53	5.56 ± 1.81	5.89 ± 1.73
BREQ‐2 domain score	Amotivation	0.13 ± 0.34	0.16 ± 0.35	0.16 ± 0.35	0.12 ± 0.26	0.21 ± 0.33	0.12 ± 0.27
	Extrinsic regulation	0.43 ± 0.54	0.50 ± 0.60	0.48 ± 0.63	0.47 ± 0.54	0.59 ± 0.59	0.34 ± 0.48^a^
	Introjected regulation	1.76 ± 0.66	1.95 ± 0.59	1.85 ± 0.65	1.58 ± 0.8	1.76 ± 0.73	1.63 ± 0.72
	Identified regulation	2.29 ± 0.35	2.24 ± 0.28	2.19 ± 0.31	2.19 ± 0.32	2.22 ± 0.30	2.12 ± 0.25
	Intrinsic regulation	2.52 ± 0.37	2.39 ± 0.38	2.38 ± 0.42	2.28 ± 0.34	2.35 ± 0.44	2.35 ± 0.41

Abbreviations: HIMT, High‐Intensity Multimodal Training; ISCT, Inter‐Session Concurrent Training; T1, baseline testing; T2, post‐testing; T3, 4 weeks follow up; SEE, Self‐Efficacy for Exercise Scale; BREQ‐2, Behavioural Regulation in Exercise Questionnaire second edition.

^a^Significant between group effect (*p <* 0.05).

Themes and meta‐inferences derived from open‐ended responses on physical activity behaviour and exercise enjoyment are presented in joint display Tables 7 and 8. Inter‐rater reliability was excellent, ICC(A, 2) = 0.98 (CI 0.969–0.987). The complete codebook and code frequencies are provided in Supporting Information S8. Participants were also given the opportunity to provide broader feedback on their training and intervention experience. Expressions of gratitude and feeling supported by interventionists were common themes. For example,

This was a very well organised and ran study. I like being able to plan ahead and your organisation and communication enabled me not to miss a session and to participate to my full abilities.

‐ Participant 48, HIMT

All the coaches were very supportive and positive. It made working out feel great.

‐ Participant 42, ISCT

## 4 Discusssion

### 4.1 Intervention Implementation

The findings of this study support the feasibility and acceptability of a community based 7‐week group training intervention, with positive perceptual outcomes observed across both groups. The intervention was well received by participants and demonstrated high recruitment and retention rates. Session attendance rates were also considered high compared to previous similar scale exercise interventions (Ballesta‐García et al. 2019; Eather et al. 2020; P. Sharp et al. 2020; Jankowski et al. 2013). These findings may reflect both the benefits offered to participants (e.g., free supervised group training, high‐level sport science testing) and the strategic, multi‐site arm's length recruitment approach. Additionally, the short intervention duration (i.e., reflecting common ‘fitness challenges’ in the community), regular engagement with interventionists during sessions and participant autonomy (i.e., selecting training times and locations) likely enhanced adherence in alignment with the SAAFE principles framework previously used in HIMT (Eather et al. 2020; Lubans et al. 2017). Despite this, artificial adherence due to the research setting (i.e., behaviour modification due to awareness of observation, frequent training session reminders) must be considered (Holden 2001; James and Vo 2010). Moreover, there is the possibility of a motivated sample bias wherein the population recruited were already recreationally active and may have been more inclined to engage in similar exercise behaviours.

A key strength of this intervention was the ecologically valid context of its delivery. In contrast to the majority of previous HIMT research, this intervention was grounded in the Applied Research Model for the Sport Sciences (ARMSS), promoting ecological validity through contextually relevant training prescription, locations and instructor preparation. This aligns with a small number of HIMT implementation studies that have similarly prioritised ecological validity (Eather et al. 2020; Kennedy et al. 2020). While this approach also expanded reach and resources, it may have introduced threats to internal validity (e.g., inter‐interventionist variability, clustering effects) which were mitigated through rigorous reporting and monitoring practices (e.g., standardised interventionist resources, preparation and session delivery across sites) (Table 10). An adapted CERT framework was also used to standardise training prescription and delivery across groups and locations, facilitating future replication and/or comparison of HIMT and ISCT interventions (T. Sharp et al. 2024; Slade et al. 2016).

**TABLE 10:** Recommendations for research and practice.

Implementation research
– Use similar feasible strategies to reduce non‐adherence in recreationally active adults. – Engage with industry collaborations to increase capacity, reach, and resources of larger scale interventions. – Rigorously report HIMT and ISCT interventions against exercise specific guidelines to enable greater transparency for replication and comparison to other studies.
Prescribing and monitoring Exercise intensity
– Use RPE as a global indicator of intensity in HIMT that more appropriately captures aerobic and resistance stimuli compared to other specific metrics (i.e., HR or % one repetition maximum). – Use RPE in HIMT as a simple tool to prescribe and monitor intensity in the community following appropriate anchoring (i.e., RIR as an adjunct anchoring tool). – Adopt similar progressive group‐training prescription models to promote safe exercise participation in recreationally active adults (i.e., manipulate training volume or exercise complexity while standardising exercise intensity).
Acceptability and perceptual outcomes
– Use group environments, supportive or educational resources, and exercise variety as strategies to promote positive perceptual responses to exercise. – A mixed method evaluation approach to examine intervention acceptability may provide greater understanding compared to one method alone. – More frequent examination (e.g., weekly or per session) of perceptual responses in future research may allow a clearer understanding of possible differences in the perceived participant experience in HIMT and ISCT.

Abbreviations: HIMT, High‐Intensity Multimodal Training; HR, heart rate; ISCT, Inter‐Session Concurrent Training; RPE, rating of perceived exertion; RIR, repetitions in reserve.

### 4.2 Training Intervention: Intensity and Progressive Overload

Previous HIMT research has demonstrated an inability to consistently prescribe high levels of exercise intensity in alignment with ACSM guidelines (American College of Sports Medicine 2025; T. Sharp et al. 2024). The combined nature of HIMT (i.e., aerobic and resistance exercise) may contribute to this challenge, wherein one single metric (e.g., HR or % one repetition maximum) cannot capture the intensity of both exercise modes (Suchomel et al. 2021; Hoeger et al. 1990). Additionally, the use of %HR_max_ or % heart rate reserve to prescribe exercise intensity may not effectively elicit category specific aerobic responses (e.g., HR zones) in all individuals (Bishop et al. 2025). The HR findings of this study support this understanding where the average %HR_max_ achieved in RT and HIMT sessions was 74.7 ± 5.7% and 74.3 ± 6.2% respectively, indicating a moderate level of intensity based on ACSM guidelines (i.e., < 77% HR_max_) (Table 6) (American College of Sports Medicine 2025). However, participants spent 55.6 ± 24.6% of HIMT session work time > 77% HR_max_, suggesting that average %HR_max_ may obscure the intermittent high‐intensity cardiovascular demands characteristic of HIMT. Among the available HR files, 19.5% were considered eligible for inclusion in this descriptive analysis, reducing the sample represented. This was likely due to interventionist, participant or software error (e.g., strap placement, upload error). These findings may highlight a trade‐off between controlled training protocols and feasible, practical exercise monitoring in the community (Costigan et al. 2020).

To address these limitations, RPE and RIR were used to monitor exercise intensity throughout the training programs (Bishop et al. 2025). In contrast to the HR findings, the average S‐RPE findings demonstrate all training session types were of a high‐intensity effort (≥ 7 RPE), reflecting the prescribed intensity (Table 6). This may be due to the more global measure of intensity that a perceptual measure can provide (i.e., captures perceptual responses to both aerobic and resistance exercise). Moreover, these findings align with previous evidence supporting the validity and reliability of RPE across aerobic, resistance, and combined aerobic and resistance training modalities (Wallace et al. 2009; Lea et al. 2022; Tibana et al. 2018; Egan et al. 2006). Notably, this use of RPE and RIR to prescribe and monitor exercise intensity (particularly in resistance exercises) in the current study aligns with recent recommendations (Bishop et al. 2025; Lovegrove et al. 2022). The use of RPE and RIR to prescribe and monitor intensity also allowed for a safe (i.e., no adverse events) and standardised method of progressive overload throughout the intervention (i.e., participants self‐selected weights or effort to meet a prescribed target RPE or RIR). Given the numerous prescriptive variables associated with HIMT (e.g., exercise selection, work: rest, intensity), this approach may overcome previous limitations in applying traditional progressive overload methods (T. Sharp et al. 2024) (Table 10). Despite the feasibility and practicality of RPE and RIR based prescription and monitoring, the limitations of self‐report, inter‐individual variability, social desirability bias and response fatigue must be acknowledged. Namely, participants were asked to report their S‐RPE a maximum of 34 times throughout the intervention. Additionally, there may be a risk of recall bias among some S‐RPE responses due to initial non‐completion or upload error directly following the session, resulting in a delayed response. The primary interventionist cross‐checked survey uploads daily and followed up any missing data within 24 h to reduce this risk.

### 4.3 Acceptability and Perceptual Outcomes

The moderate increase in PA levels from baseline to post‐test in both groups suggest that the prescribed programme was feasible and tolerable for recreationally active adults. This may be partially attributed to the motivated sample bias, where the majority of participants were already “sufficiently” active (i.e., Godin LTPAQ score > 24, HIMT: 41.15 ± 19.84, ISCT: 43.76 ± 23.49) (Godin 2011). This is further supported by the open‐ended survey responses where participants reported that participation in the study increased their perceived knowledge, skills and confidence for exercise mode, equipment and intensity which may have supported increases in PA despite no significant changes in self‐efficacy. e.g., *“I am more confident using weights, so this encourages me to go to the gym.”‐ Participant 75, HIMT.* Additionally, participants reported that the routine of the intervention was physically and logistically achievable (e.g., “*I found it easy to fit it into my routine which I wasn't expecting*.”–*Participant 59, ISCT*). Similarly, behavioural regulation scores remained stable throughout the intervention, yet participants reported many positive contributions to extrinsic (e.g., “*Seeing the tangible results in strength and cardio gain makes you want to keep improving”*–*Participant 45, HIMT*) and intrinsic motivators (e.g., *“I think I have just been amazed with the benefits, mood, emotional regulation, feeling more confident in my body”*–*Participant 77, ISCT*). In contrast, some participants reported their PA participation and motivation to be impacted by common barriers including time and other commitments (e.g., “*I was busy with my schedule”*–*Participant 13, HIMT)* (Australian Instituate of Health and Welfare 2018). These barriers may explain the moderate decrease in PA levels demonstrated in the ISCT group from post‐test to follow up. The integration of quantitative and qualitative findings suggests that increases in physical activity were most likely when the intervention provided a routine and participants were aware of their progress. This is consistent with Self‐Determination Theory and Social Cognitive Theory, wherein factors such as autonomy, competency, self‐efficacy and self‐regulation are associated with behaviour change (Deci and Ryan 1985; Bandura 1991). The intervention also reinforced the exercise behaviour of participants who were previously engaged in similar PA behaviour (i.e., exceeding PA guidelines). However, less active individuals (i.e., meeting PA guidelines) found it more challenging to maintain this behaviour without the support of the intervention.

Despite high adherence and session attendance rates, both the HIMT and ISCT groups demonstrated low to moderate exercise enjoyment scores at baseline, post‐test and follow‐up. Enjoyment levels significantly increased from baseline to post‐test and baseline to follow‐up in the ISCT group compared to the HIMT group (*p* = 0.025, *p* = 0.048). While these outcomes reached moderate statistical significance below the conventional threshold (*p* < 0.05), this did not meet the Bonferri‐corrected threshold (*p* < 0.006). Despite this, these findings align with the nuanced understanding of enjoyment in high‐intensity exercise and warrant consideration. The mixed methods analysis revealed a disconnect between the quantitative and qualitative data as well as a manifestation of enjoyment in high‐intensity exercise through multiple pathways. Both groups demonstrated low to moderate enjoyment scores, aligning with previous research reporting feelings of pain, displeasure and reduced affective valence as associated with high‐intensity aerobic exercise (in comparison to moderate or low intensity activity) (Kilpatrick et al. 2014; Jung and Little 2013; Howard et al. 2022; Tavares et al. 2021). Despite this, self‐reported reasons for enjoyment in the groups were underpinned by both similar (e.g., sense of achievement and supportive group environment) and distinct factors (e.g., structural variety of HIMT vs. routine predictability of ISCT). Previous research supports this wherein, HIMT participation has been associated with favourable perceptual responses, including exercise enjoyment and intrinsic factors relating to adherence (Eather et al. 2020; Heinrich et al. 2014; Batrakoulis et al. 2020; T. Sharp et al. 2023). Additionally, comparable interval‐based exercise has been suggested to provide more variety in stimulus and more frequent recovery compared to continuous exercise, contributing to an enjoyable experience (Kilpatrick et al. 2014; Jung and Little 2013; T. Sharp et al. 2023; Thum et al. 2017). The divergence of the low to moderate enjoyment scores and positive open‐ended responses may partially be attributed to limitations of the PACES‐S instrument, wherein the terminology used may not have appropriately captured the positive affective responses described (e.g., inclusion of the term pleasurable or pleasant). Further examination of additional outcomes such as subjective training quality and perceived recovery of HIMT and ISCT may contribute to a greater understanding of the perceptual responses to these training modes (Shell et al. 2023) (Table 10).

The risk of recall bias and inter‐individual variation in interpretation of survey instruments must also be acknowledged. To overcome this, participants were familiarised with the surveys at baseline. Moreover, more frequent examination of exercise enjoyment (i.e., per session), self‐efficacy and behavioural regulation (e.g., weekly) may have provided greater insight into the perceptual responses to HIMT and ISCT. However, participant burden must be considered. It is also possible that a longer period of training or follow up may have been associated with greater changes in self‐efficacy or behavioural regulation (i.e., up to 10–24 weeks) (Rodgers et al. 2010). Despite low to moderate exercise enjoyment, self‐efficacy and behavioural regulation scores at T1, T2 and T3, participants demonstrated high adherence and session attendance rates. This may indicate that the artificial research environment created a sense of accountability amongst participants that outweighed their perceptual responses to the training (Holden 2001). Additionally, the open‐ended responses suggest positive perceptual outcomes associated with the training modes. From a pragmatic mixed‐methods perspective, the integration of quantitative and qualitative data provides a more practical understanding of exercise experiences, that recognises both measurable outcomes and the participant‐centred factors rather than quantitative or qualitative alone. Moreover, more frequent examination of these perceptual responses among others (e.g., training quality) may provide greater insight into the participant experience in HIMT and ISCT. This understanding may contribute to more tailored service delivery in the community and research, thereby promoting long‐term PA behaviours and intervention adherence.

## 5 Conclusion

This study supports the feasibility and acceptability of 7 weeks of HIMT and ISCT in recreationally active adults, with positive perceptual outcomes demonstrated across both groups. High adherence and attendance were observed, likely attributed to the structured research context and supportive group‐training environment. The mixed‐method evaluation revealed divergence between quantitative and qualitative data, reinforcing the value of integrating data sources when assessing acceptability and perceptual outcomes. To enhance adherence, acceptability and perceptual outcomes in future HIMT interventions or practice, researchers and practitioners may consider progressive group‐training models, opportunities for participant autonomy, exercise variety, and practical intensity‐monitoring tools (e.g., RPE, RIR). Although collaborating with industry partners may have introduced threats to internal validity, these partnerships increased delivery capacity and improved ecological validity. Transparent reporting aligned with exercise‐specific guidelines remains essential for replicability. While the inherent variability of HIMT presents implementation challenges, employing feasible, standardised strategies can support consistent engagement and promote meaningful health outcomes in recreationally active populations.

**Abbreviations**
ACSM: American College of Sports Medicine
AES: Accredited Exercise Scientist
APSS: Adult pre‐exercise screening tool
ARMSS: Applied Research Model for the Sport Sciences
AT: Aerobic Training
BMI: Body Mass Index
bpm: Beats per minute
BREQ‐2: Behavioural Regulation in Exercise Questionnaire second edition
CERT: Consensus on Exercise Reporting Template
cm: Centimetres
CONSORT: CONsolidated Standards of Reporting Trials
ESSA: Exercise and Sports Science Australia
F: Female
GUIDED: Guidance for reporting intervention development studies in health research
HIMT: High‐Intensity Multimodal Training
HR: Heart rate
HR_max_
: Heart rate maximum
ISCT: Inter‐Session Concurrent Training
kg: Kilograms
LTPAQ: Leisure Time Physical Activity Questionnaire
M: Male
m^2^
: Metres squared
n: Number
PA: Physical Activity
PACES‐S: Shortened Version of the Physical Activity Enjoyment Scale
PRS: Perceived Recovery Scale
RIR: Repetitions in Reserve
RPE: Rating of Perceived Exertion
RT: Resistance Training
S‐RPE: Session Rating of Perceived Exertion
SAAFE: Supportive, Active, Autonomous, Fair and Enjoyable
SD: Standard deviation
SEE: Self‐Efficacy for Exercise Scale
SPIRIT: Standard Protocol Items:Recommendations for Interventional Trials
T1: Baseline testing
T2: Post‐testing
T3: 4 weeks follow up
TQ: Training Quality
VAS: Visual Analogue Scale
$\dot{V}O_{2}$_peak_
: Peak oxygen uptake
W:R: Work to rest ratio
y: Years

## Funding

This research was funded by an Australian Government Research Training Programme (RTP) PhD Stipend Scholarship, Faculty Funding by the University of Technology Sydney and with an in‐kind donation from Douglas Hanly Moir Pathology for venous blood sample analysis.

## Ethics Statement

Ethical approval for this trial was obtained from the University of Technology Sydney Research Ethics Board (reference no ETH24‐9364). Participants provided informed consent and completed the ESSA APSS with the primary researcher prior to all baseline assessments. Participants were also informed that they may withdraw from the study at any time, for any reason, without consequence. Amendments to the study protocol publicly are available via the Australian and New Zealand Clinical Trials Registry (trial number: ACTRN12624000556549). Data management procedures were conducted by the first and last authors. All collected data was de‐identified using participants codes and stored electronically in a password‐protected drive at the University. The subsequent findings of the study will be disseminated through national and international academic meetings, through peer‐reviewed publication and by web‐based activities (e.g., podcasts, research webinars). Additionally, findings may also be disseminated through social media (e.g., Facebook, Twitter). Finally, participants received a personal and de‐identified group results summary of the study findings.

## Consent

All participants provided written informed consent prior to participation.

## Conflicts of Interest

The authors declare no conflicts of interest.

## Supporting information


Supporting Information S1

Supporting Information S2

Supporting Information S3

Supporting Information S4

Supporting Information S5

Supporting Information S6

Supporting Information S7

Supporting Information S8

## Data Availability

Data will be provided upon reasonable request to the corresponding author.
