# Supplementary figures and images for: Community‐Based High‐Intensity Multimodal Training: A Mixed‐Method Evaluation of a Randomised Control Trial
Source: Eur J Sport Sci. 2026 Jun 26;26(7):e70211. doi: 10.1002/ejsc.70211 (PMC13309294; doi:10.1002/ejsc.70211)

**Supplementary Material 1** Consensus for Exercise Reporting Template


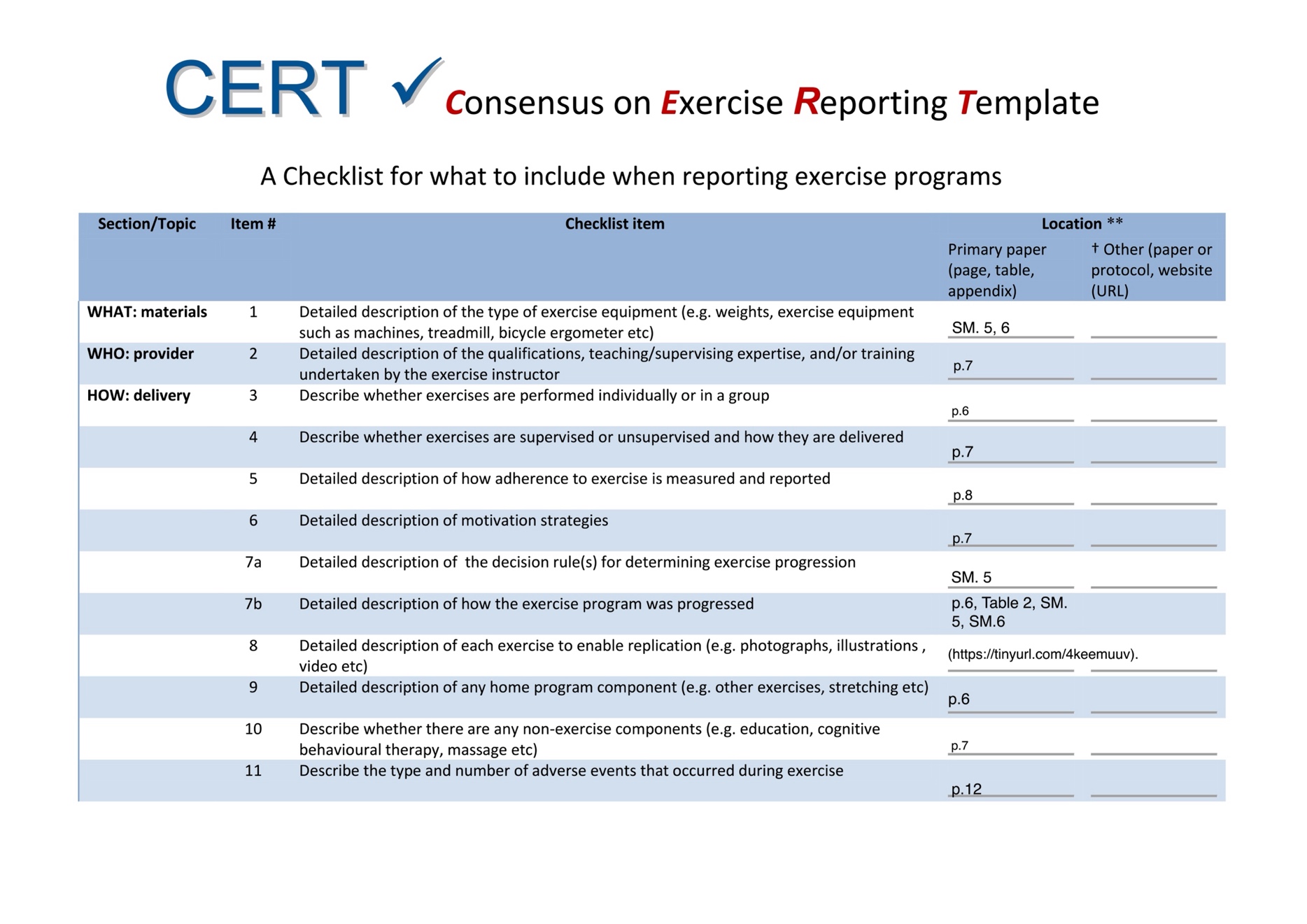


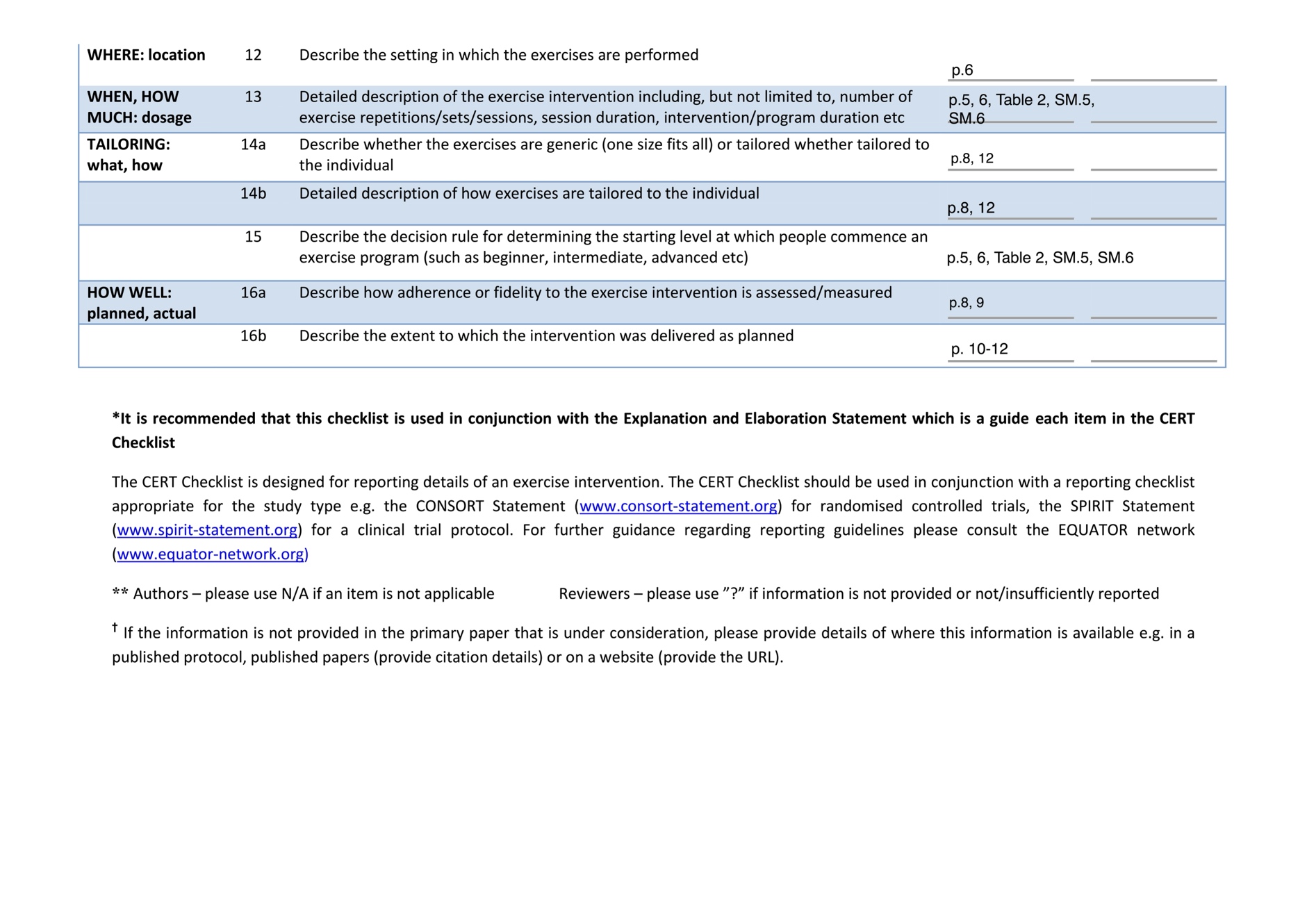

Supplement: Supplementary file 1 — Supporting Information S1 [file EJSC-26-e70211-s008.docx]

**Supplementary Material 4** GUIDED Checklist
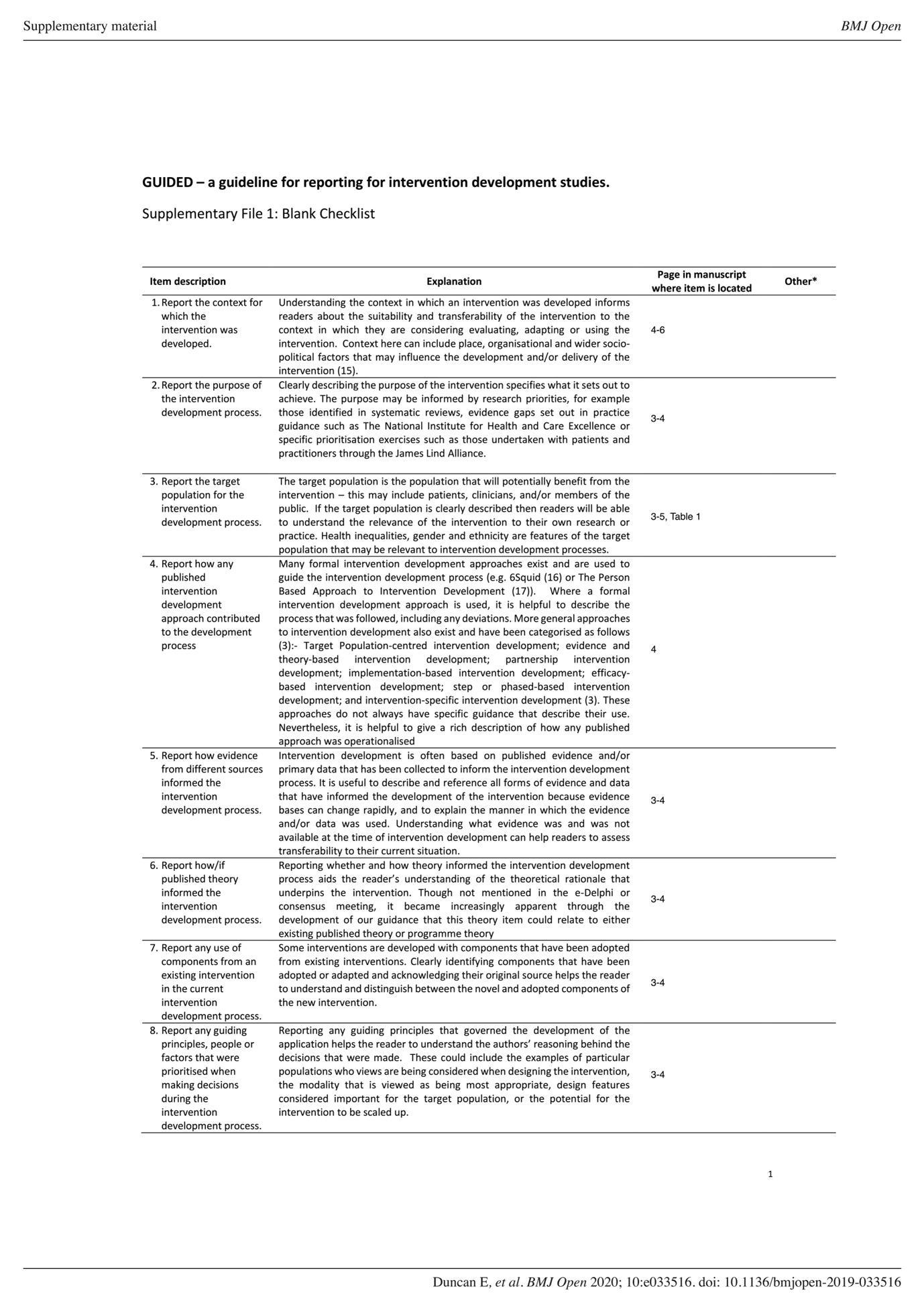

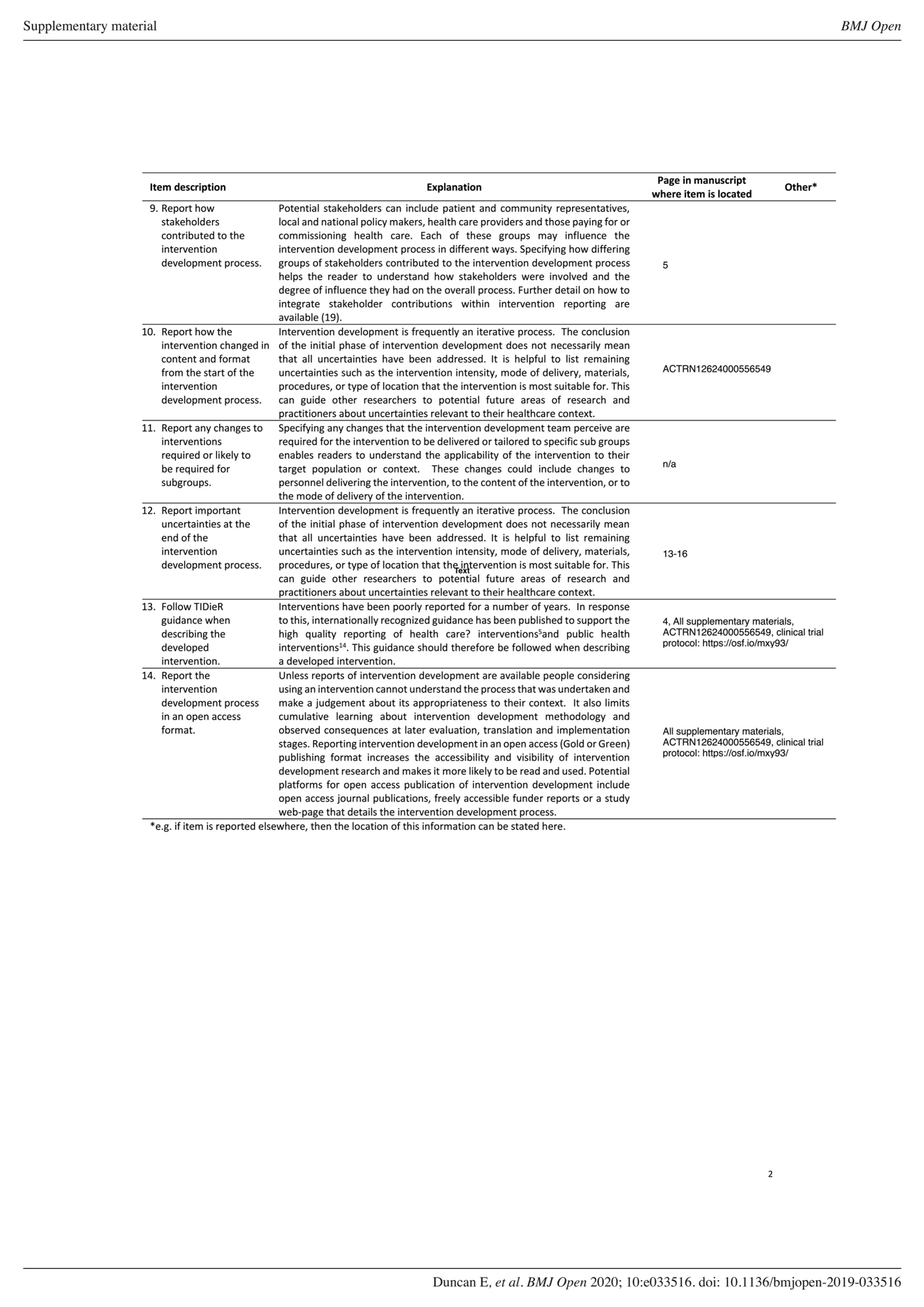

Supplement: Supplementary file 4 — Supporting Information S4 [file EJSC-26-e70211-s006.docx]
