# Supplementary material for: Community‐Based High‐Intensity Multimodal Training: A Mixed‐Method Evaluation of a Randomised Control Trial
Source: Eur J Sport Sci. 2026 Jun 26;26(7):e70211. doi: 10.1002/ejsc.70211 (PMC13309294; doi:10.1002/ejsc.70211)

**Supplementary Material 3** Methodological Checklist for Studies of Pleasure and Enjoyment Responses to High-Intensity Interval Training


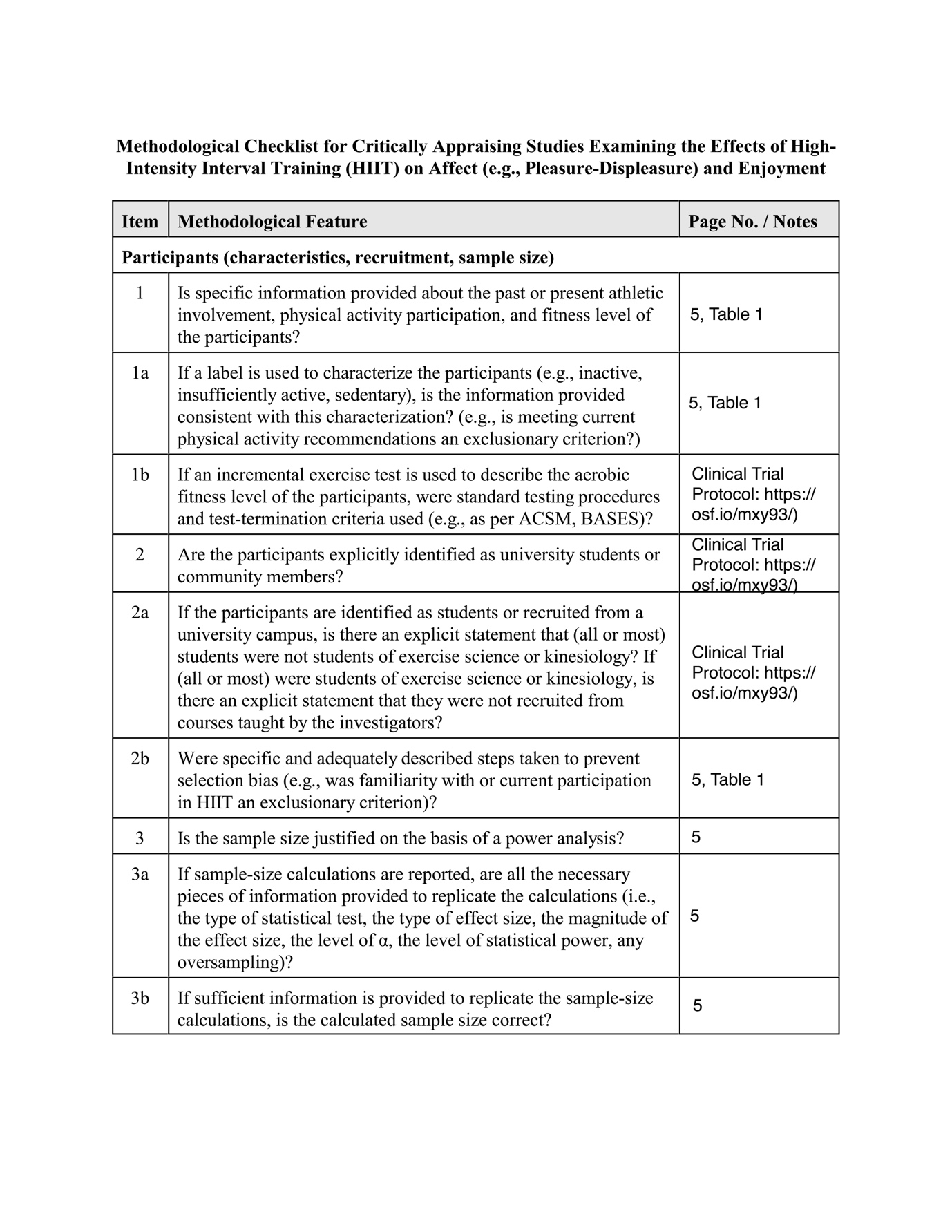


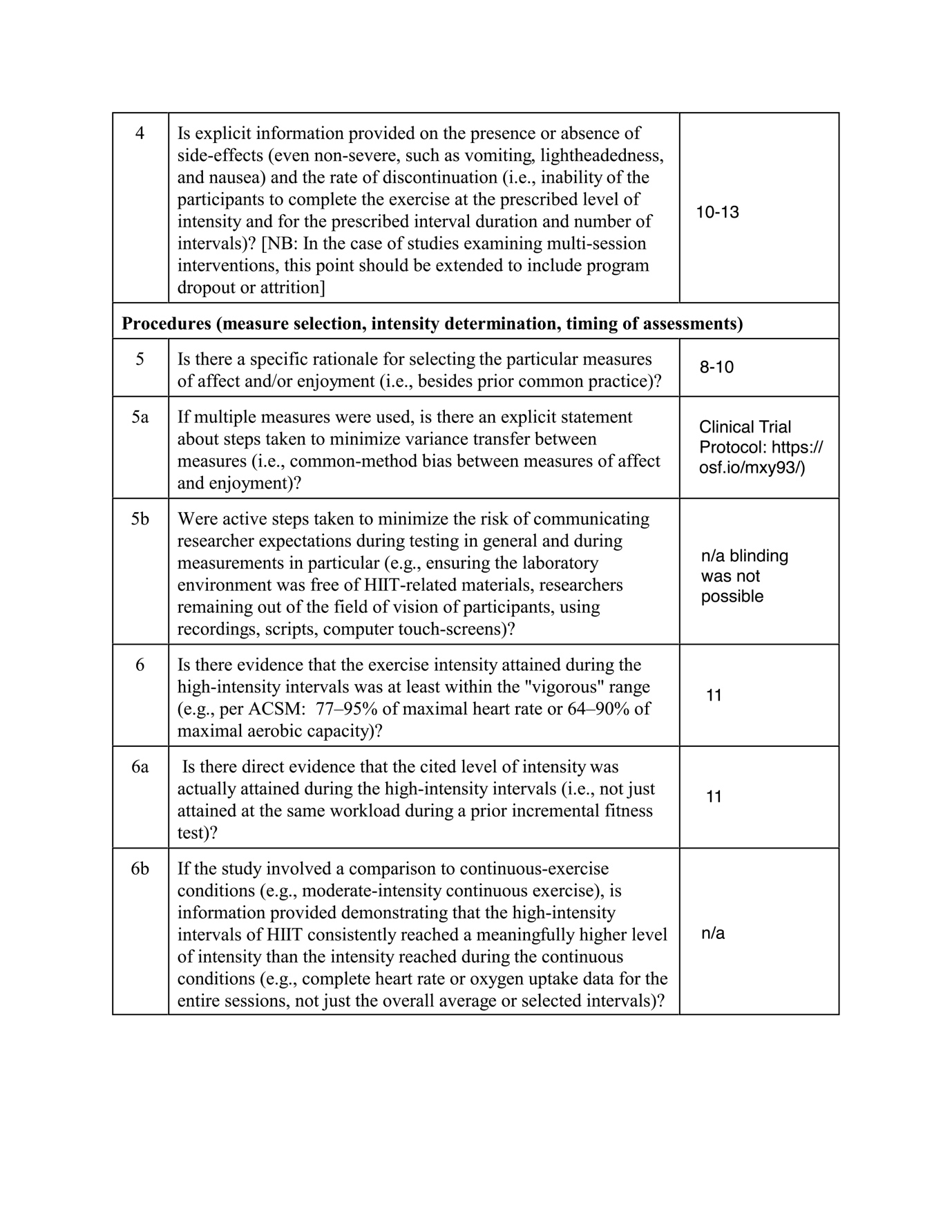

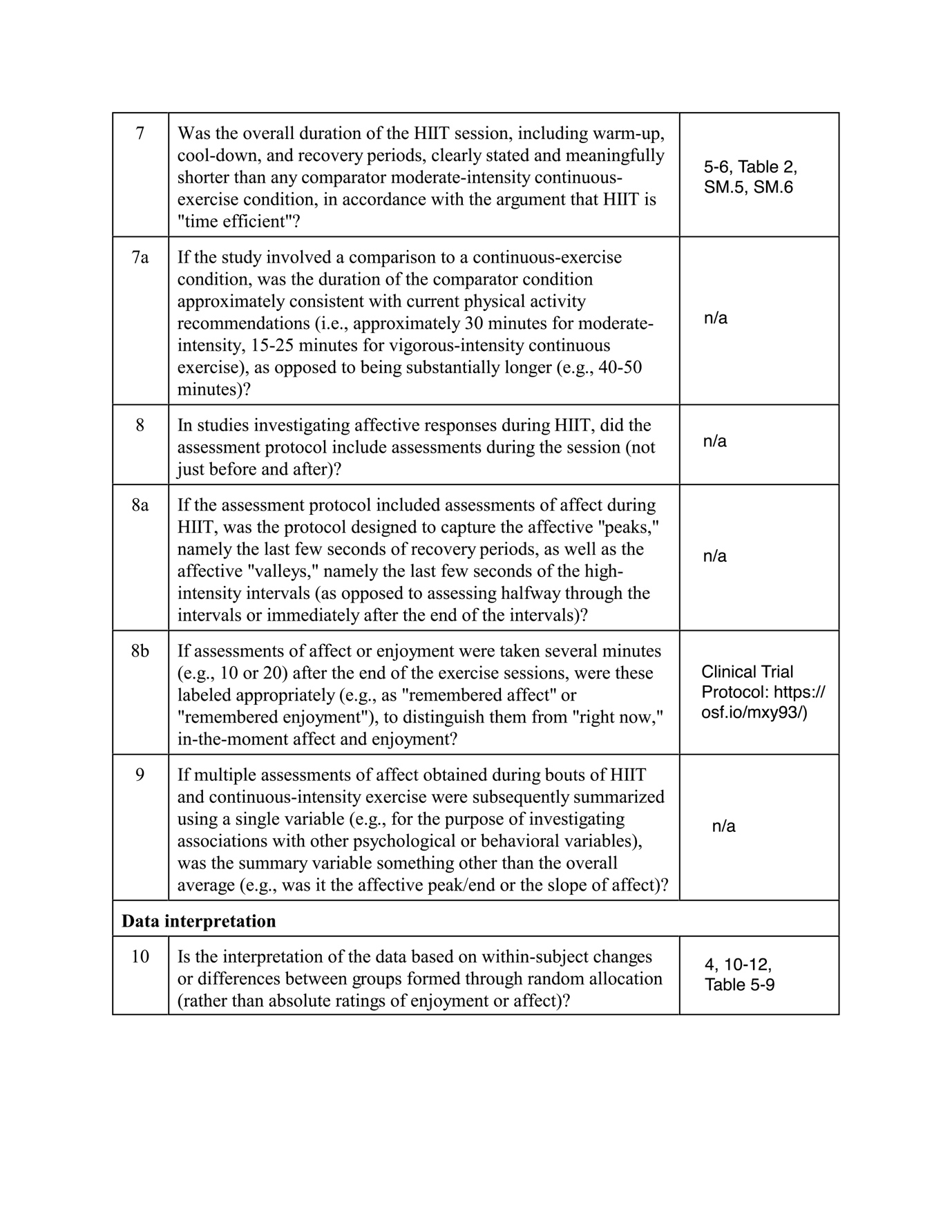

Supplement: Supplementary file 3 — Supporting Information S3 [file EJSC-26-e70211-s007.docx]
