# Supplementary material for: Community‐Based High‐Intensity Multimodal Training: A Mixed‐Method Evaluation of a Randomised Control Trial
Source: Eur J Sport Sci. 2026 Jun 26;26(7):e70211. doi: 10.1002/ejsc.70211 (PMC13309294; doi:10.1002/ejsc.70211)
Supplement: Supplementary file 5 — Supporting Information S5 [file EJSC-26-e70211-s002.docx]

**Supplementary Material 5** Training Program Exercise Prescription Details

| Frequency | HIMT: 4 different x 45 minute sessions/ week.  ISCT: 2 different x 45 minute aerobic + 2 different x 45 minute resistance sessions/ week. |
| --- | --- |
| Session Duration | Total 45 minutes (included ~5 minute warm up and cool down) |
| Session Format | Exercises were performed in a circuit format during a session. Sessions involved one or more circuits to ensure variety. |
| Exercise Selection | Each training week included the following movement patterns: hinge, squat, rotation, push, pull. Exercise selection for HIMT and ISCT changed fortnightly to ensure variety and progressive overload. Sessions in a training week were matched for exercise selection across both HIMT and ISCT programs (i.e., HIMT sessions included both aerobic and resistance exercises. ISCT aerobic sessions included the same aerobic exercises as the HIMT sessions and ISCT resistance sessions included the same resistance exercises as the HIMT sessions). |
| Exercise Equipment | HIMT and ISCT programs used the same exercise equipment as exercise selection is matched across a training week. All training locations had access to the same equipment to ensure standardisation.  Exercise equipment included ergometer machine (e.g., cycle and row ergometers), free weights (e.g., dumbbells, kettlebells, medicine balls), skipping ropes and exercise mats. Exercise equipment (i.e., exercise selection) varied fortnightly to ensure variety. |
| Exercise Order | Exercises were ordered in a session to reduce fatigue across muscle groups (i.e., alternate lower body/ upper body exercises). |
| Prescribed Volume | Total and net volume was matched for HIMT and ISCT protocols during a training week.  Time based volume prescription was primarily utilised (e.g., 45 seconds of work with 15 seconds of rest to transition to next exercise). Work to rest ratios varied fortnightly throughout the program to ensure variety. Work to rest ratios increased across the program as a means of progressive overload.  The initial starting external load for participants in the baseline testing sessions (days 2, 3, 4) was prescribed by the primary researcher based on the familiarisation activity during baseline testing (day 1). External loads for week 1 of training were adjusted if required based on testing days 2, 3, 4.  Participants were instructed to always aim to perform 10 reps of a resistance exercise at the prescribed intensity. This allowed standardisation in prescribed volume-load. |
| Prescribed Intensity | Participants were instructed to work at an RPE ≥ 7/10 (CR-10) (‘very hard’) to ensure high-intensity activity (1). Participants were familiarised with the RPE scale during baseline testing Days 2, 3 and 4 (2).  Aerobic exercises (e.g., ergometer): Participants were instructed to:   - “Work as hard as you can to maintain a ≥ 7/10 RPE” - “You should not be able to hold a conversation during the work period” - “You should be able to regain your breath within the rest period and move to the next station appropriately.”   Resistance exercises (e.g., weights or bodyweight) :   - “Aim to complete 10 reps using the heaviest weight you can for this rep range maintaining a ≥ 7/10 RPE.” - “Perform each exercise with at least a 2 second lower and 1 second lift.” - “If you finish the 10 reps before the work period is over - that is okay you can rest for the remaining time rather than performing more reps if you achieved ≥ 7/10 RPE.” - “If you do not reach ≥ 7/10 RPE by 10 reps, complete more reps so that you achieve this RPE, and then increase your external load for the next set as guided by your instructor (e.g., 9kg DB goblet squat, increase to 10kg DB goblet squat).” - “You should aim to feel like you have 3 reps left in the tank for each set”.   10 reps was chosen to reflect common prescription in HIMT (i.e., ~10RM, strength-hypertrophy stimulus) (3) |
| Tempo | Aerobic exercises (e.g., ergometer): Participants were instructed to:   - “Work as hard as you can to maintain a ≥ 7/10 RPE”   Resistance exercises (e.g., weights or bodyweight) :   - “Perform each exercise with at least a 2 second lower and 1 second lift.”   This tempo was prescribed for resistance exercises to ensure appropriate exercise technique and safety. |
| Monitored Volume | The training volume-load (i.e., weight lifted in resistance exercises x reps completed or distance covered x ergometer resistance) was recorded each session by participants. |
| Monitored Intensity | RPE ≥ 7/10 or 3 ≤ RIR  HR (continuously during), S-RPE (post session). |
| Progressive Overload | Participants were encouraged to increase the weights lifted during a work period when they could complete > 10 reps in the work period and remain at an RPE < 7/10 or > 3 RIR. Participants were encouraged to increase this weight by approximately 5-10% (or at the discretion of the instructor) the next time that exercise was performed to achieve a rep range of 10 and RPE ≥ 7 RPE or 3 ≤ RIR.  Participants were encouraged to increase distance achieved on ergometers while maintain an RPE ≥ 7. |
| Regression | Participants were encouraged to decrease the weights lifted during a work period when they were unable to complete 10 reps in the work period.  If a participant was observed to be working to fatigue or exhaustion, or with inappropriate technique instructors advised the participant to reduce either:   - the external load (i.e., weights, ergometer resistance) - the reps completed (while remaining in the prescribed 10R rep range) - tempo at which they are performing the exercise - difficulty of the exercise (e.g., push up on toes may be regressed to kneeling push up). |
| Modifications/ scaling | All exercises were pre-prescribed with scaled alternatives available to suit varying abilities of participants and promote appropriate level of exercise intensity (e.g., kneeling push up, push up on toes, decline push up). Instructors explained all alternatives when delivering the session overview and participants were free to choose their starting point. Instructors would then encourage participants to progress or regress these alternatives based on the above progression/ regression methods.  In the event that a participant was unable to perform an exercise, instructors modified the exercise based on pre-prescribed modifications to the program. Instructors were provided with this information when they receive each training program. Instructors recorded events of modification with the participants’ training volume-load diary for the session. |
| Supervision | All training sessions were completely supervised by qualified exercise professionals (i.e., ESSA AES, Personal Trainers). Third year sport and exercise science students undergoing their placement assisted in session instruction and training volume-load recording. Sessions were performed in small groups of 3-8 participants (i.e., 1 research assistant: maximum 8 participants). |
| Encouragement/ motivation strategies | Instructors frequently used phrases such as “great work”, “keep it up”, among other positive phrases. These strategies were anchored in instructors in a familiarisation session with the primary researcher to ensure standardisation. |
| Exercise technique | Appropriate exercise technique was explained by instructors during the familiarisation sessions (baseline testing) and each time a novel exercise was introduced. Instructors were educated on appropriate exercise technique in a familiarisation session with the primary researcher to ensure standardisation. |
| Training location (physical environment) | All training locations were indoor, weather protected facilities. All locations had access to the same training equipment to ensure standardisation across locations. |
| Music | All training sessions played background music >170bpm via a speaker. |

HIMT, *high-intensity multimodal training,* ISCT, *inter-session concurrent training,* RPE, *rating of perceived exertion,* kg, *kilogram,* DB, *dumbbell,* RM, *repetition maximum,* RIR, *repetitions in reserve,* ESSA AES, *Exercise and Sports Science Australia, Accredited Exercise Scientist,* bpm, *beats per minute*

**References**

1. ACSM ACSM’s guidelines for exercise testing and prescription: 12th edition. Philadelphia : Wolters Kluwer, 2025.

2. Borg G. Perceived exertion as an indicator of somatic stress. Scand J Rehabil Med. 1970;2(2):92-8.

3. Sharp T, Slattery K, Coutts AJ, van Gogh M, Ralph L, Wallace L. Solving the High-Intensity Multimodal Training Prescription Puzzle: A Systematic Mapping Review. Sports Medicine - Open. 2024;10(1):82.
