# Supplementary material for: Community‐Based High‐Intensity Multimodal Training: A Mixed‐Method Evaluation of a Randomised Control Trial
Source: Eur J Sport Sci. 2026 Jun 26;26(7):e70211. doi: 10.1002/ejsc.70211 (PMC13309294; doi:10.1002/ejsc.70211)

**Supplementary Material 7** Perceptual Responses Statistical Analysis

# Outcome: glsi

## Number of Participants Included: 79

## Distribution of DV at Baseline


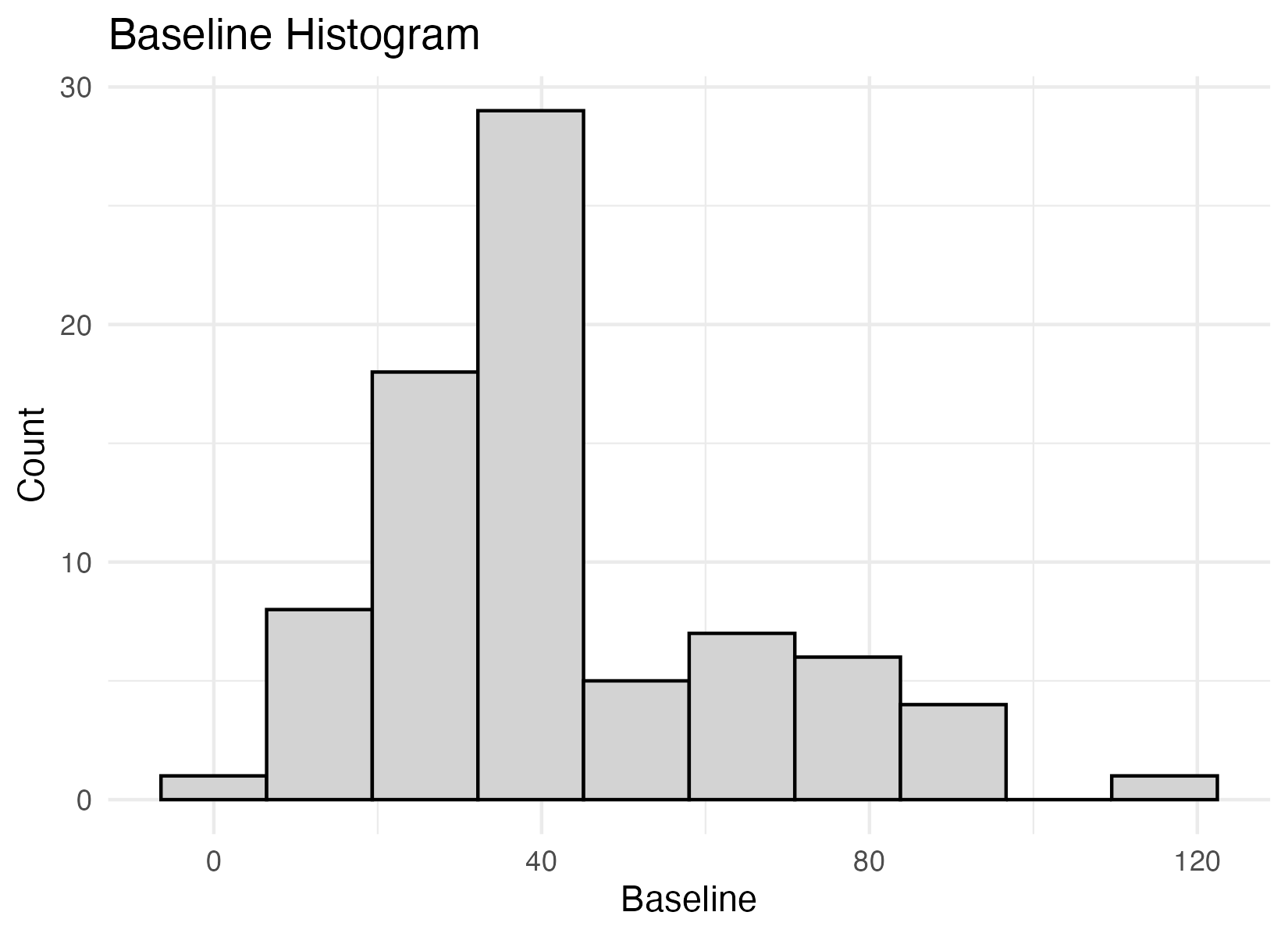


## Fitted vs Residuals


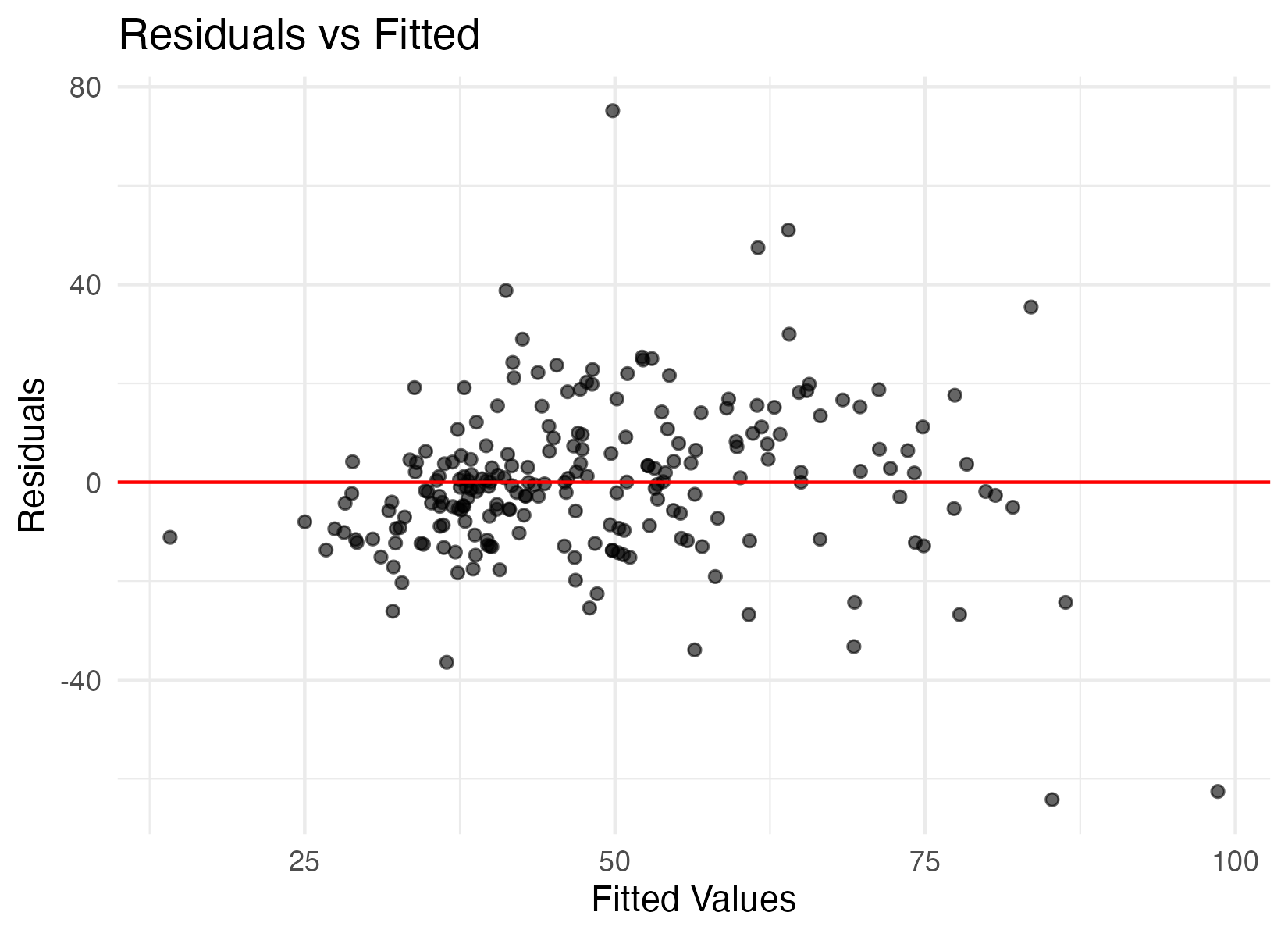


## QQ Plot


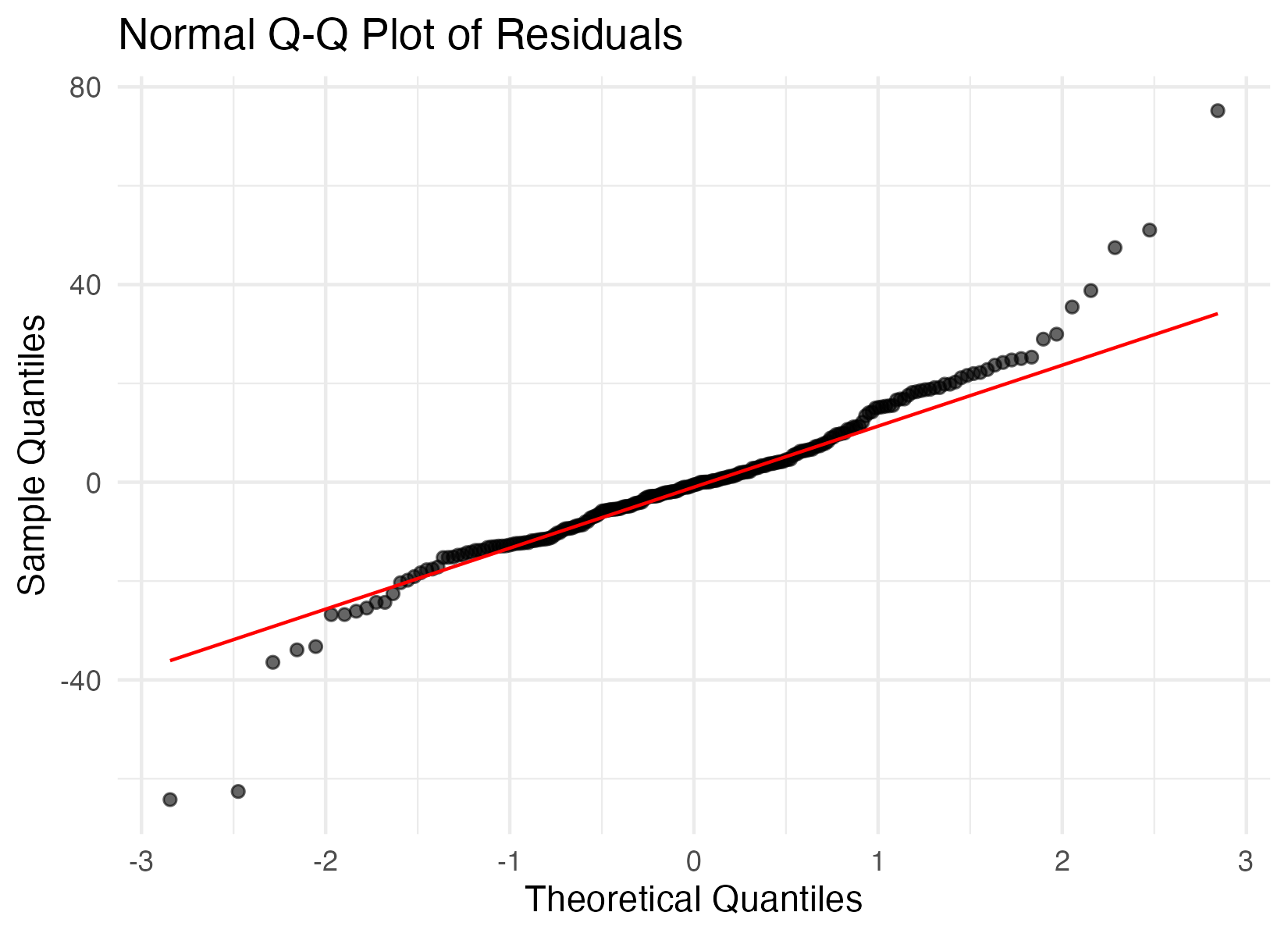


## Within-group change (baseline to follow-up)

| contrast | group | estimate | SE | df | lower.CL | upper.CL | t.ratio | p.value | effect_size |
| --- | --- | --- | --- | --- | --- | --- | --- | --- | --- |
| T2 - T1 | C | 12.311 | 3.661 | 212 | 3.669 | 20.953 | 3.362 | 0.003 | 0.569 |
| T3 - T1 | C | 3.845 | 3.690 | 212 | -4.864 | 12.554 | 1.042 | 0.551 | 0.178 |
| T3 - T2 | C | -8.466 | 3.772 | 212 | -17.369 | 0.437 | -2.244 | 0.066 | -0.391 |
| T2 - T1 | S | 15.047 | 3.722 | 212 | 6.262 | 23.832 | 4.043 | <0.001 | 0.695 |
| T3 - T1 | S | 1.694 | 3.751 | 212 | -7.160 | 10.548 | 0.452 | 0.894 | 0.078 |
| T3 - T2 | S | -13.353 | 3.773 | 212 | -22.258 | -4.448 | -3.539 | 0.001 | -0.617 |

## Between-group difference in change (interaction)

| timepoint_revpairwise | group_revpairwise | estimate | SE | df | lower.CL | upper.CL | t.ratio | p.value | effect_size |
| --- | --- | --- | --- | --- | --- | --- | --- | --- | --- |
| T2 - T1 | S - C | 2.736 | 5.220 | 212 | -7.554 | 13.026 | 0.524 | 0.601 | 0.126 |
| T3 - T1 | S - C | -2.151 | 5.260 | 212 | -12.520 | 8.218 | -0.409 | 0.683 | -0.100 |
| T3 - T2 | S - C | -4.887 | 5.335 | 212 | -15.404 | 5.630 | -0.916 | 0.361 | -0.226 |

## Adjusted Means Over Time (with 95% CI)


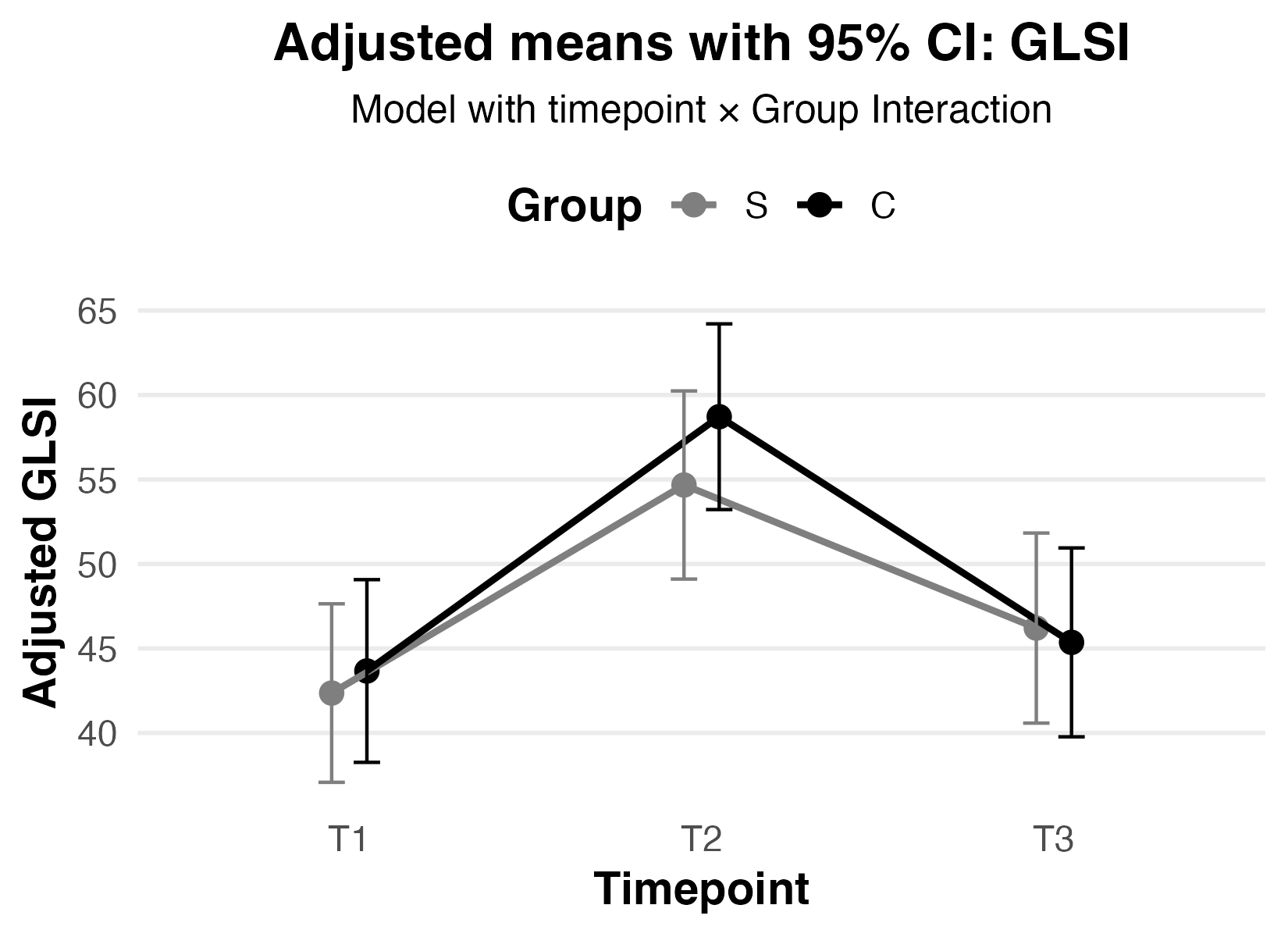


# Outcome: pacesscore

## Number of Participants Included: 79

## Distribution of DV at Baseline


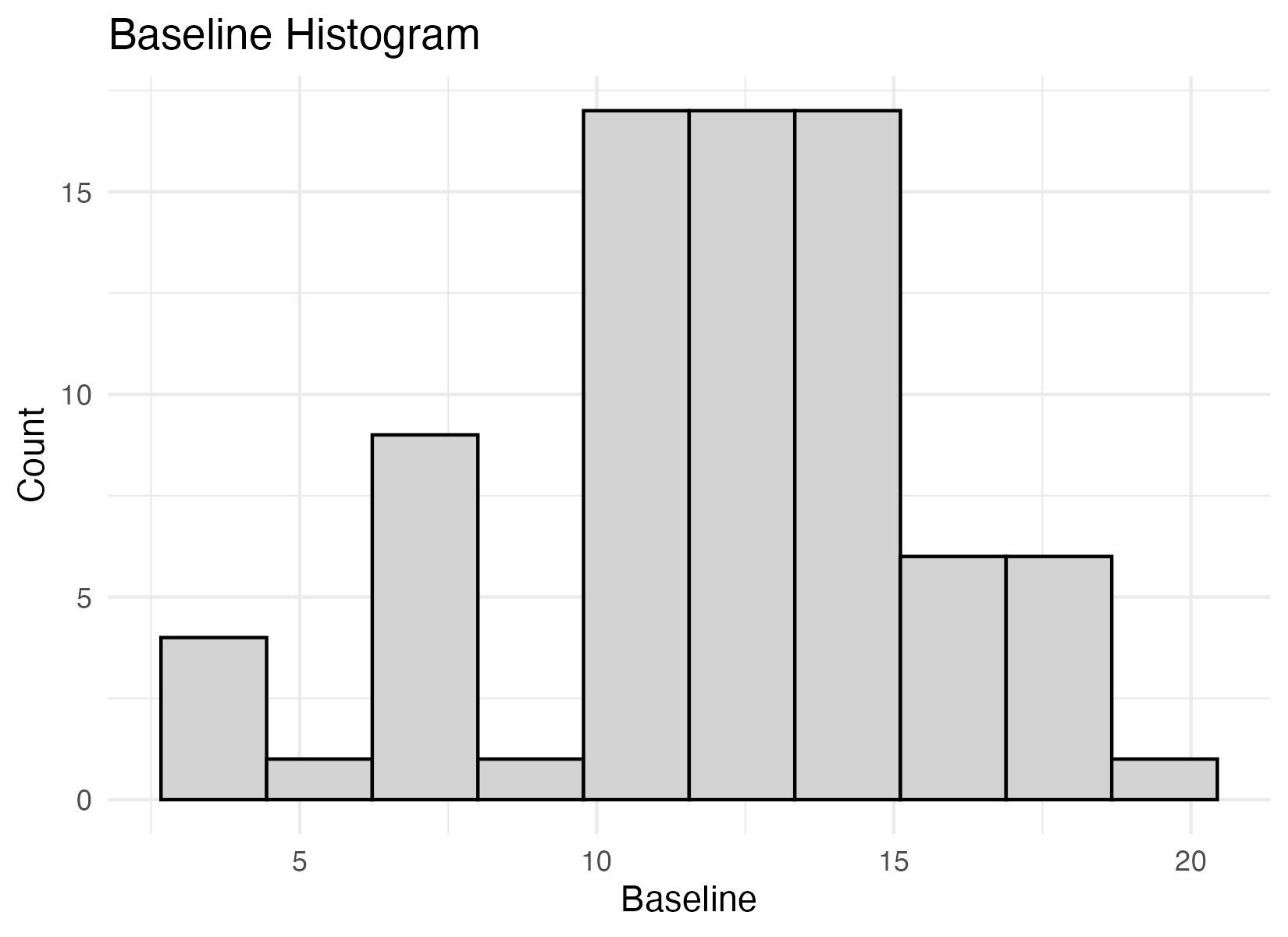


## Fitted vs Residuals


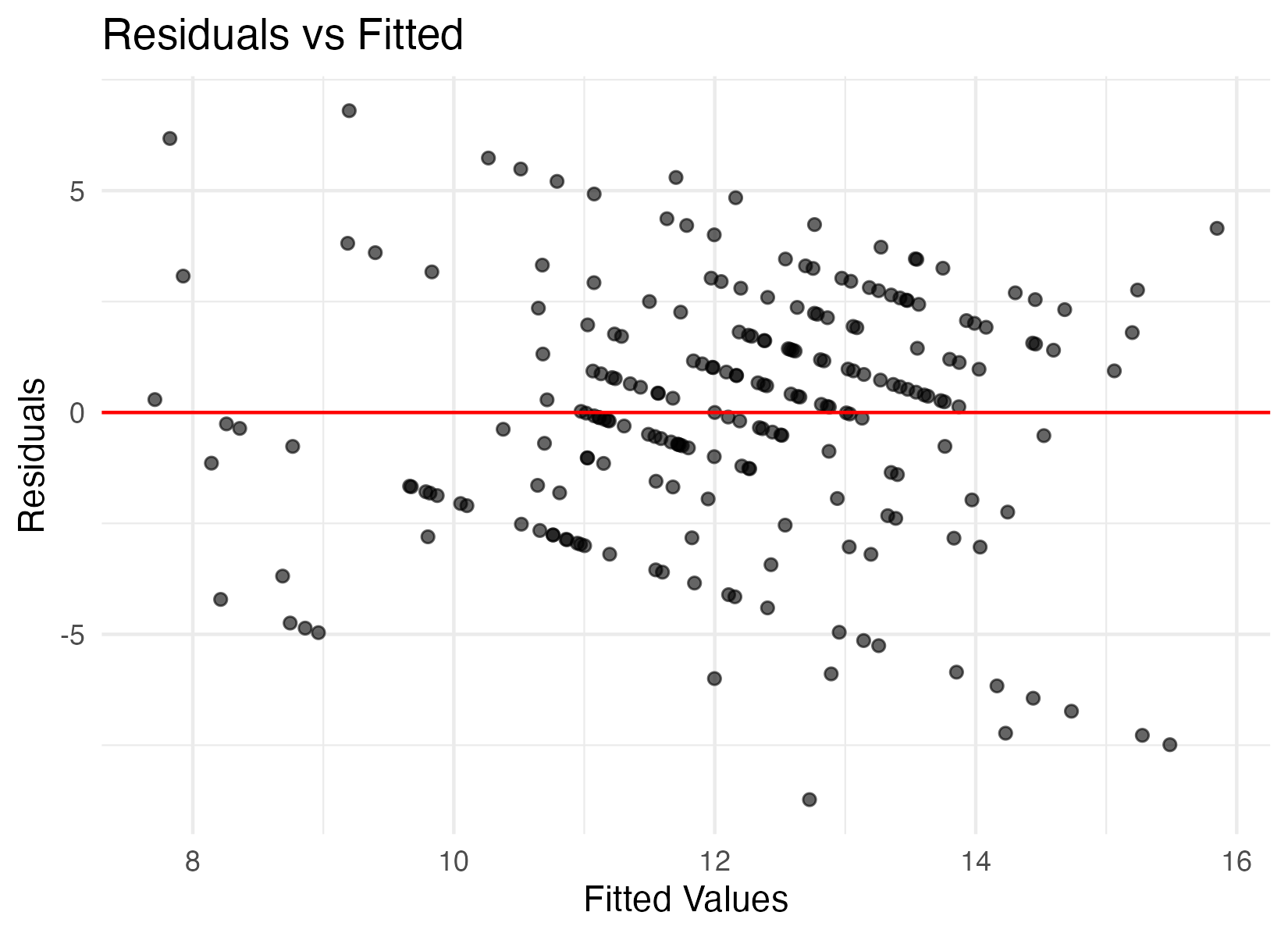


## QQ Plot


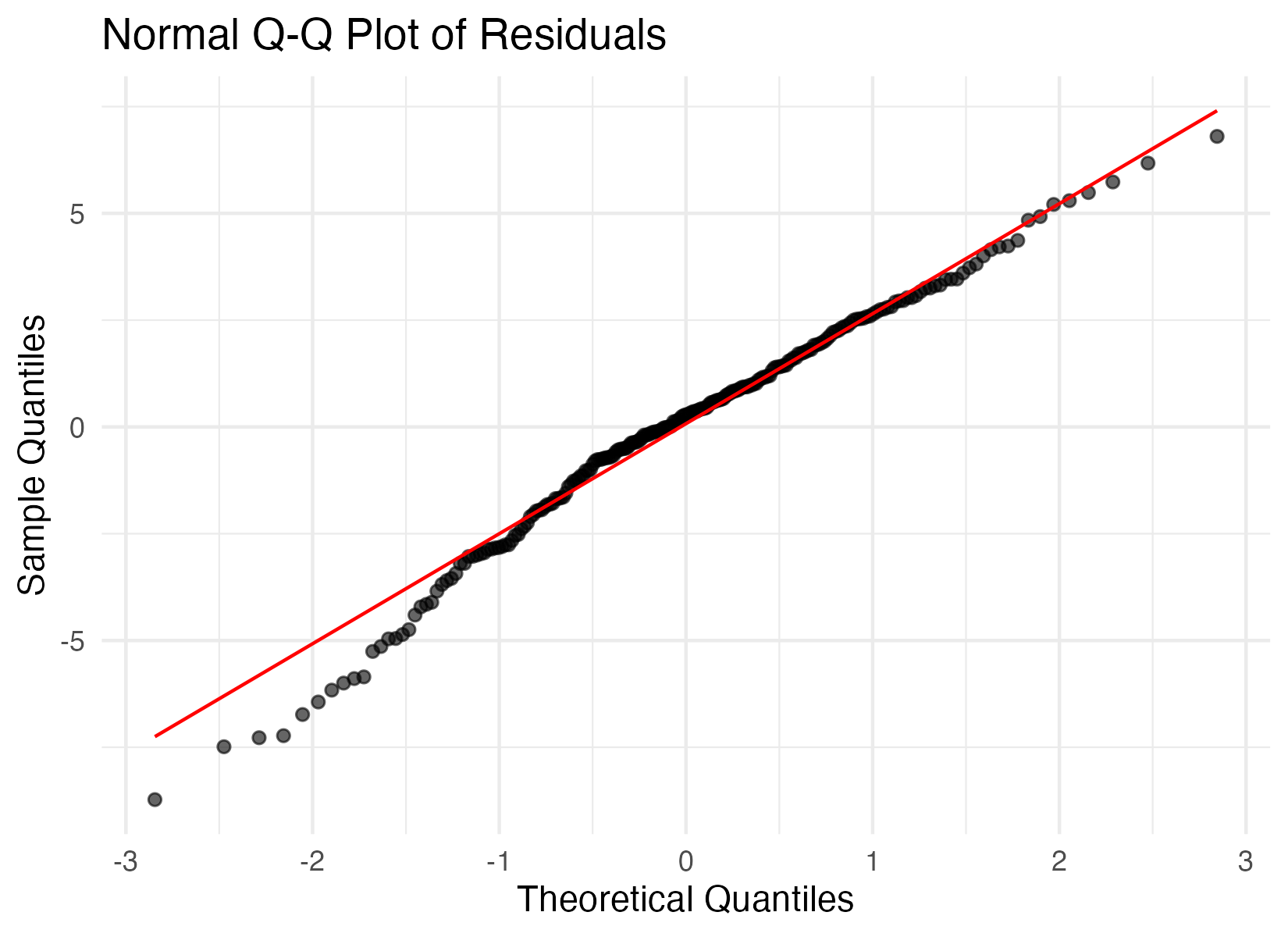


## Within-group change (baseline to follow-up)

| contrast | group | estimate | SE | df | lower.CL | upper.CL | t.ratio | p.value | effect_size |
| --- | --- | --- | --- | --- | --- | --- | --- | --- | --- |
| T2 - T1 | C | -1.037 | 0.627 | 212 | -2.518 | 0.444 | -1.652 | 0.226 | -0.326 |
| T3 - T1 | C | -0.603 | 0.632 | 212 | -2.095 | 0.888 | -0.955 | 0.606 | -0.190 |
| T3 - T2 | C | 0.433 | 0.647 | 212 | -1.094 | 1.961 | 0.670 | 0.781 | 0.136 |
| T2 - T1 | S | 0.974 | 0.638 | 212 | -0.533 | 2.481 | 1.525 | 0.281 | 0.306 |
| T3 - T1 | S | 1.185 | 0.643 | 212 | -0.333 | 2.703 | 1.842 | 0.158 | 0.372 |
| T3 - T2 | S | 0.211 | 0.647 | 212 | -1.316 | 1.739 | 0.326 | 0.943 | 0.066 |

## Between-group difference in change (interaction)

| timepoint_revpairwise | group_revpairwise | estimate | SE | df | lower.CL | upper.CL | t.ratio | p.value | effect_size |
| --- | --- | --- | --- | --- | --- | --- | --- | --- | --- |
| T2 - T1 | S - C | 2.010 | 0.895 | 212 | 0.246 | 3.775 | 2.246 | 0.026 | 0.632 |
| T3 - T1 | S - C | 1.788 | 0.902 | 212 | 0.011 | 3.566 | 1.983 | 0.049 | 0.562 |
| T3 - T2 | S - C | -0.222 | 0.915 | 212 | -2.026 | 1.582 | -0.243 | 0.809 | -0.070 |

## Adjusted Means Over Time (with 95% CI)


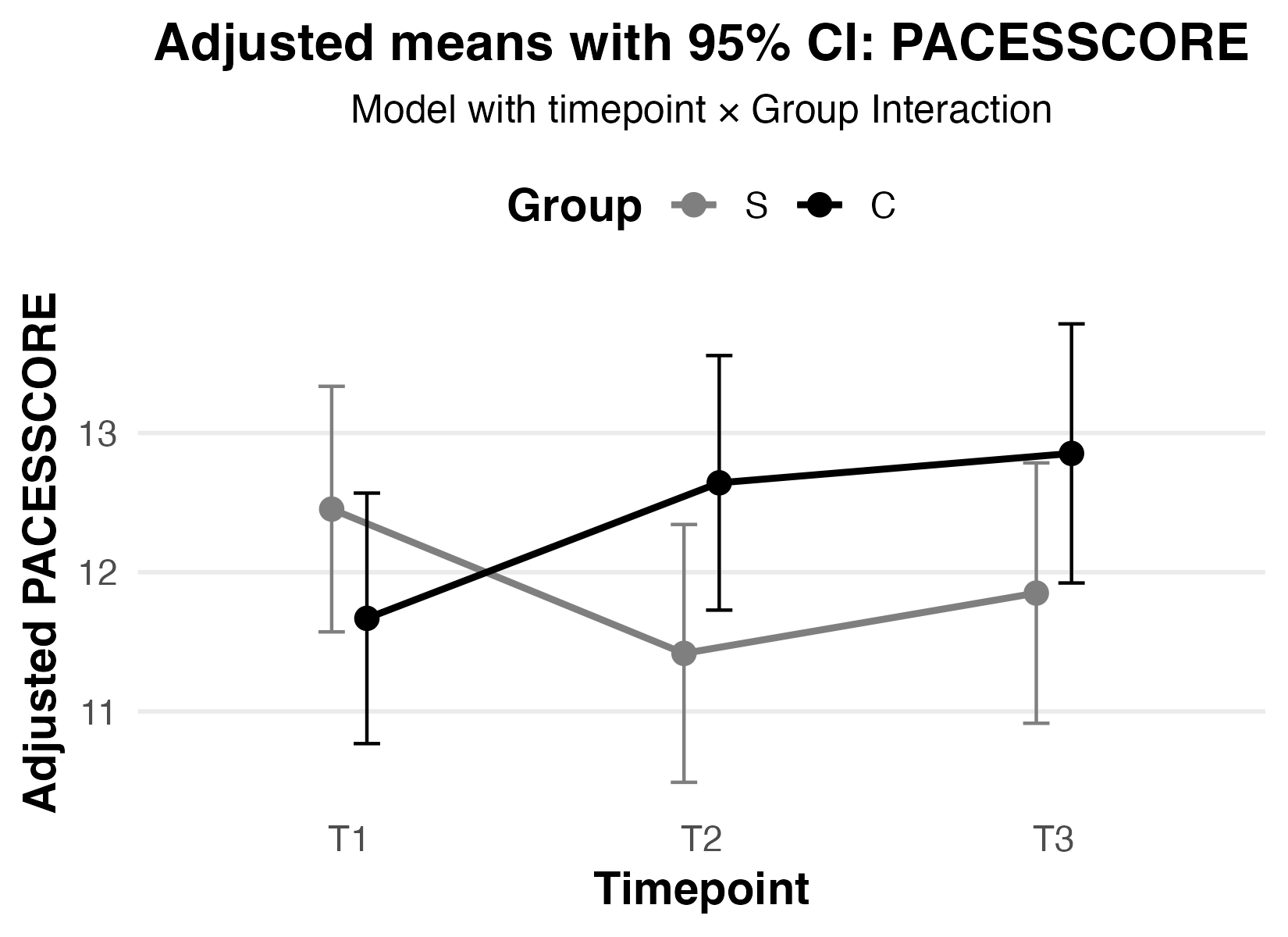


# Outcome: sescore

## Number of Participants Included: 79

## Distribution of DV at Baseline


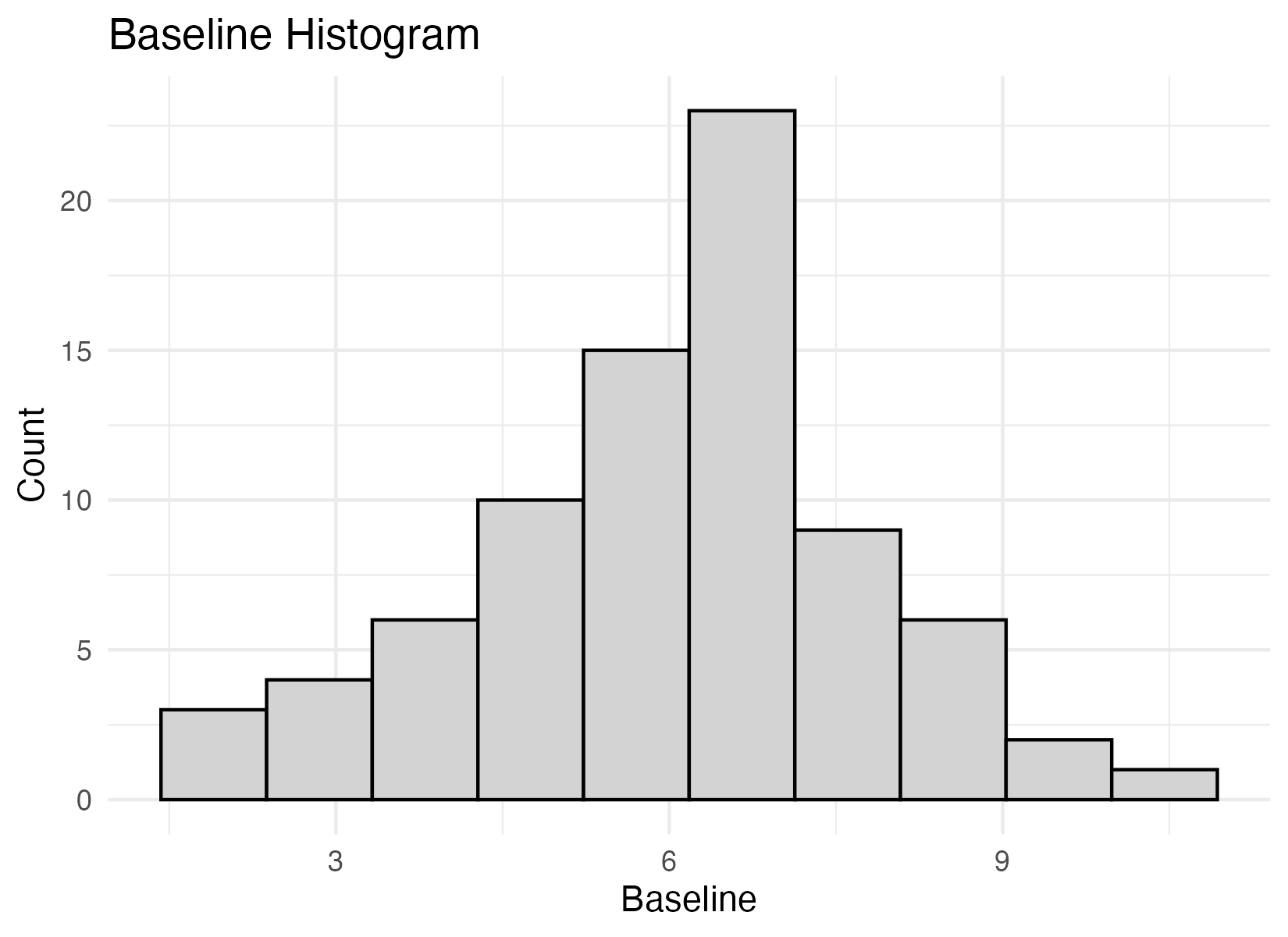


## Fitted vs Residuals


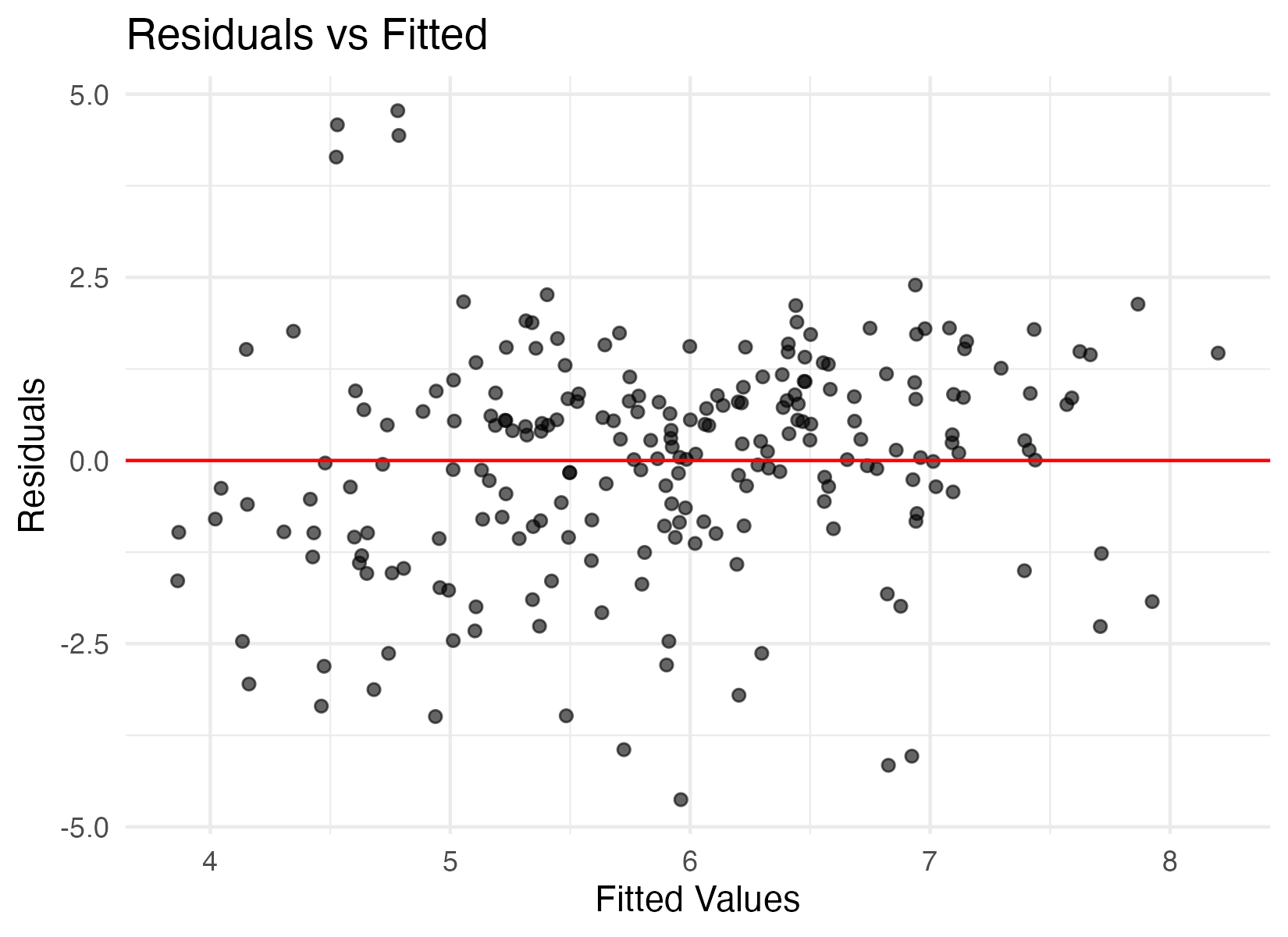


## QQ Plot


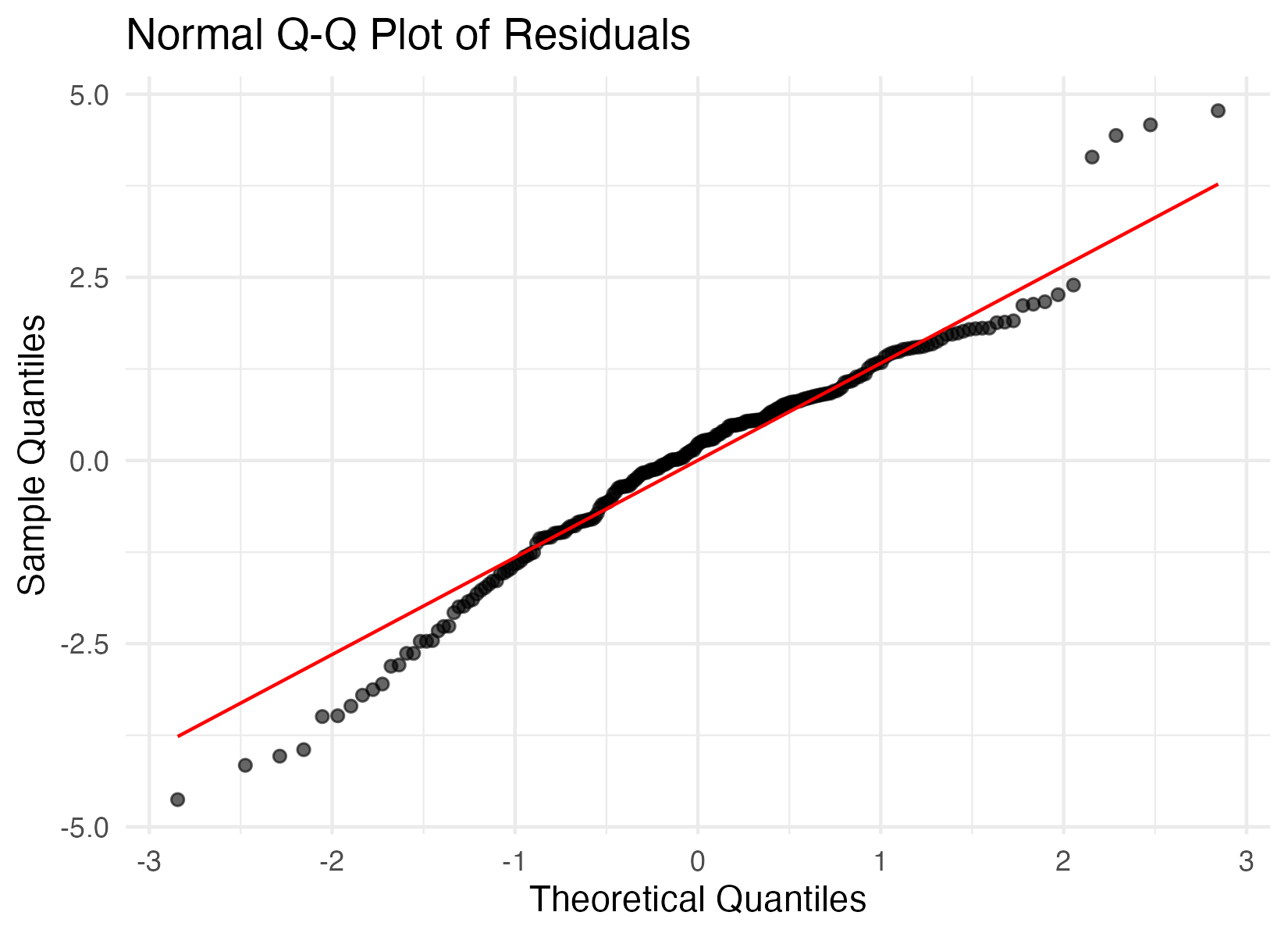


## Within-group change (baseline to follow-up)

| contrast | group | estimate | SE | df | lower.CL | upper.CL | t.ratio | p.value | effect_size |
| --- | --- | --- | --- | --- | --- | --- | --- | --- | --- |
| T2 - T1 | C | -0.152 | 0.359 | 212 | -0.999 | 0.694 | -0.425 | 0.905 | -0.080 |
| T3 - T1 | C | -0.157 | 0.361 | 212 | -1.010 | 0.696 | -0.434 | 0.901 | -0.083 |
| T3 - T2 | C | -0.005 | 0.369 | 212 | -0.876 | 0.867 | -0.012 | 1.000 | -0.002 |
| T2 - T1 | S | -0.576 | 0.364 | 212 | -1.436 | 0.284 | -1.582 | 0.256 | -0.304 |
| T3 - T1 | S | -0.275 | 0.367 | 212 | -1.142 | 0.592 | -0.748 | 0.735 | -0.145 |
| T3 - T2 | S | 0.302 | 0.369 | 212 | -0.570 | 1.173 | 0.817 | 0.693 | 0.159 |

## Between-group difference in change (interaction)

| timepoint_revpairwise | group_revpairwise | estimate | SE | df | lower.CL | upper.CL | t.ratio | p.value | effect_size |
| --- | --- | --- | --- | --- | --- | --- | --- | --- | --- |
| T2 - T1 | S - C | -0.424 | 0.511 | 212 | -1.431 | 0.583 | -0.830 | 0.408 | -0.224 |
| T3 - T1 | S - C | -0.118 | 0.515 | 212 | -1.133 | 0.898 | -0.229 | 0.819 | -0.062 |
| T3 - T2 | S - C | 0.306 | 0.522 | 212 | -0.723 | 1.335 | 0.586 | 0.558 | 0.162 |

## Adjusted Means Over Time (with 95% CI)


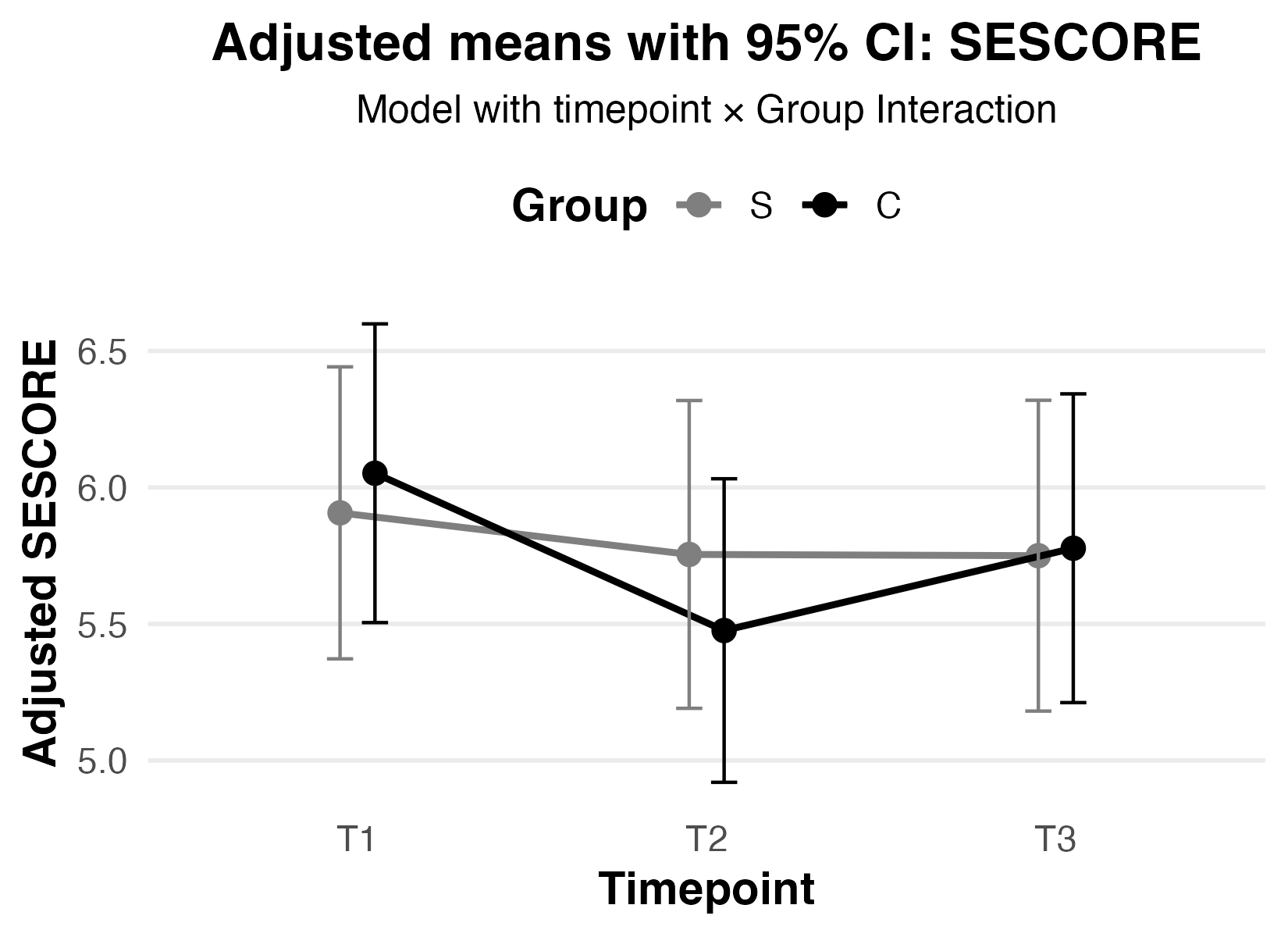


# Outcome: amotivation

## Number of Participants Included: 79

## Distribution of DV at Baseline


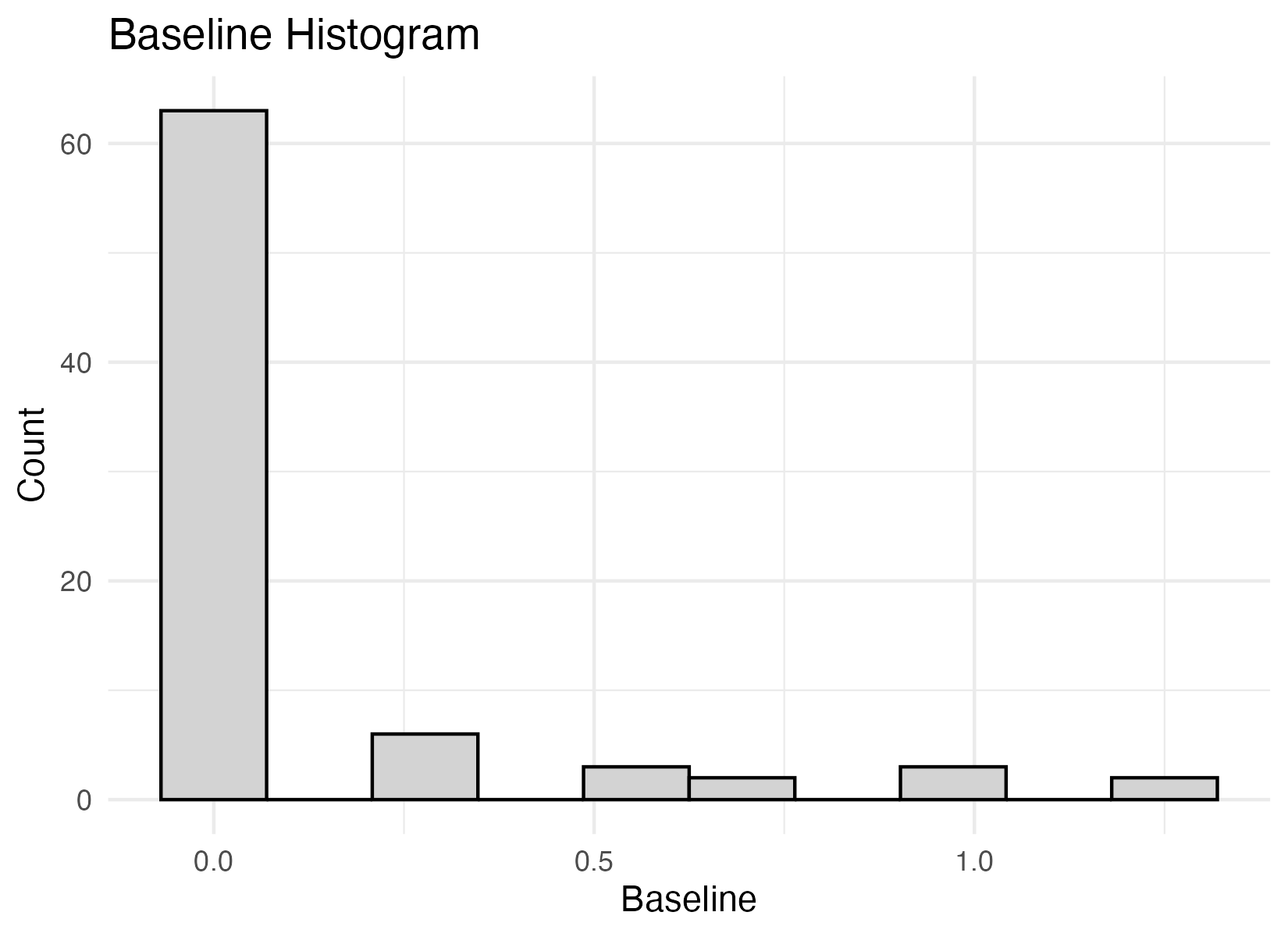


## Fitted vs Residuals


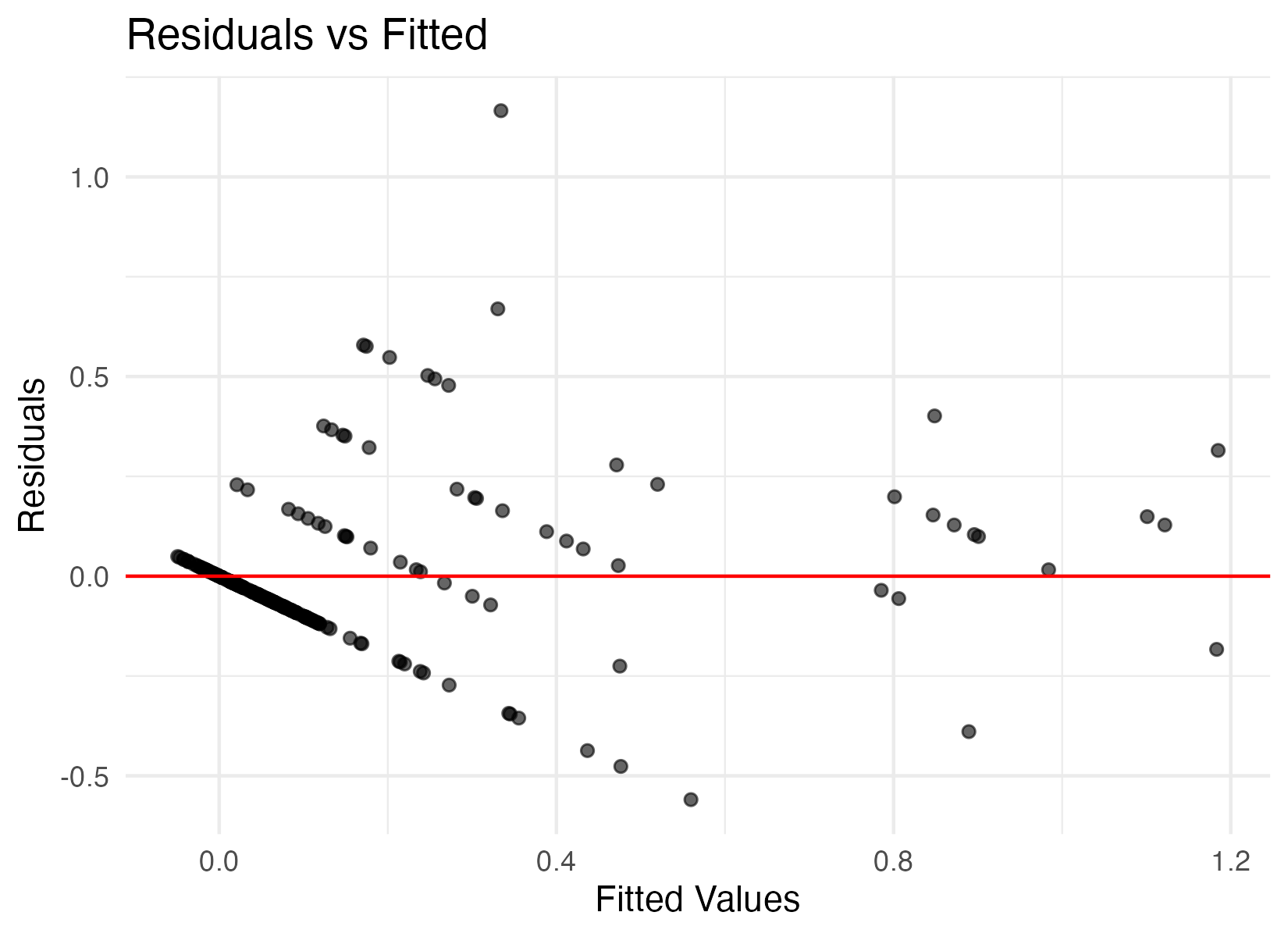


## QQ Plot


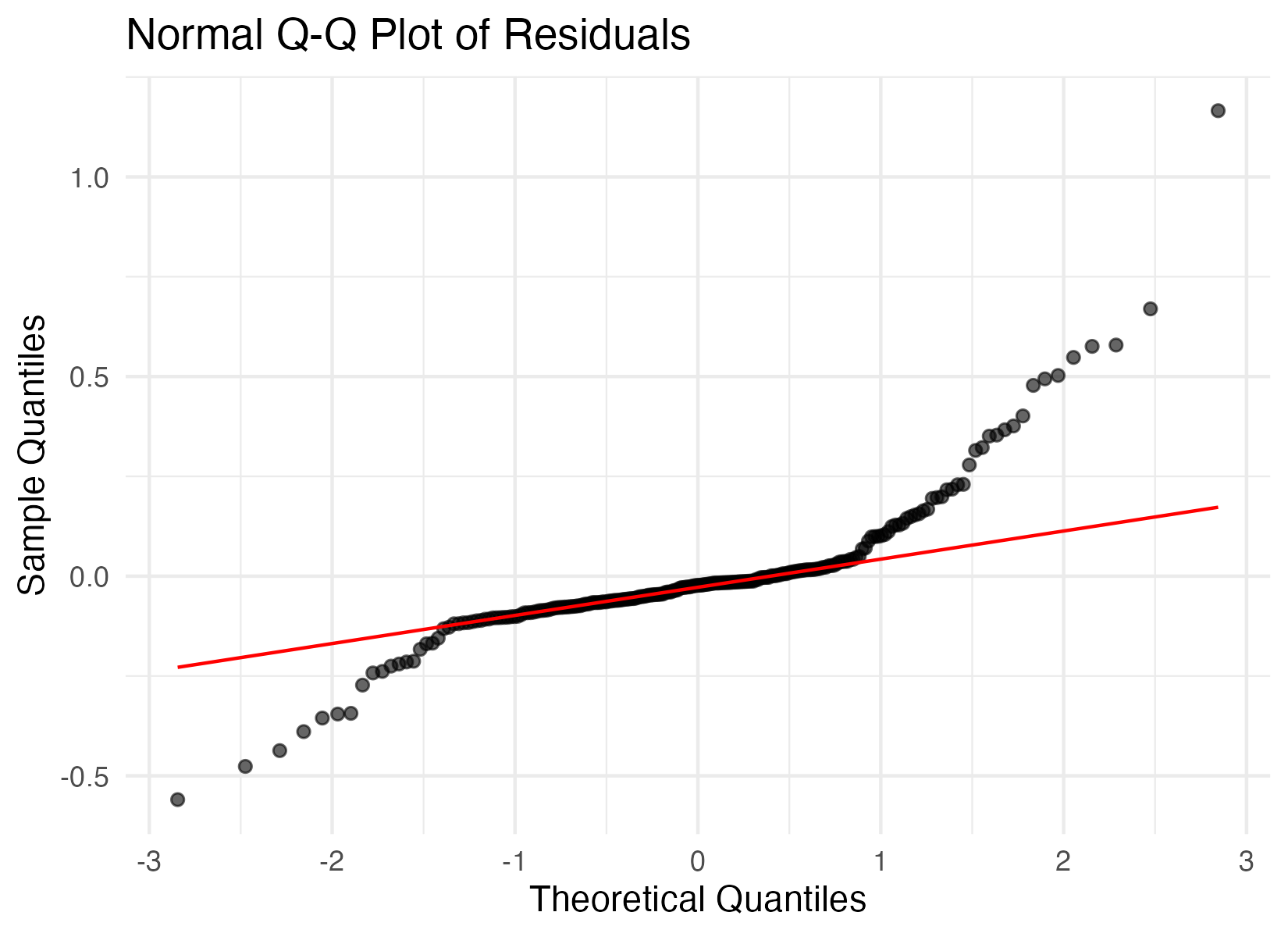


## Within-group change (baseline to follow-up)

| contrast | group | estimate | SE | df | lower.CL | upper.CL | t.ratio | p.value | effect_size |
| --- | --- | --- | --- | --- | --- | --- | --- | --- | --- |
| T2 - T1 | C | 0.061 | 0.044 | 212 | -0.043 | 0.166 | 1.387 | 0.350 | 0.194 |
| T3 - T1 | C | 0.063 | 0.045 | 212 | -0.042 | 0.169 | 1.416 | 0.334 | 0.200 |
| T3 - T2 | C | 0.002 | 0.046 | 212 | -0.106 | 0.109 | 0.039 | 0.999 | 0.006 |
| T2 - T1 | S | 0.088 | 0.045 | 212 | -0.018 | 0.194 | 1.964 | 0.124 | 0.279 |
| T3 - T1 | S | 0.005 | 0.045 | 212 | -0.102 | 0.112 | 0.111 | 0.993 | 0.016 |
| T3 - T2 | S | -0.083 | 0.046 | 212 | -0.191 | 0.024 | -1.827 | 0.163 | -0.263 |

## Between-group difference in change (interaction)

| timepoint_revpairwise | group_revpairwise | estimate | SE | df | lower.CL | upper.CL | t.ratio | p.value | effect_size |
| --- | --- | --- | --- | --- | --- | --- | --- | --- | --- |
| T2 - T1 | S - C | 0.027 | 0.063 | 212 | -0.098 | 0.151 | 0.423 | 0.672 | 0.085 |
| T3 - T1 | S - C | -0.058 | 0.064 | 212 | -0.184 | 0.067 | -0.916 | 0.361 | -0.184 |
| T3 - T2 | S - C | -0.085 | 0.064 | 212 | -0.212 | 0.042 | -1.320 | 0.188 | -0.269 |

## Adjusted Means Over Time (with 95% CI)


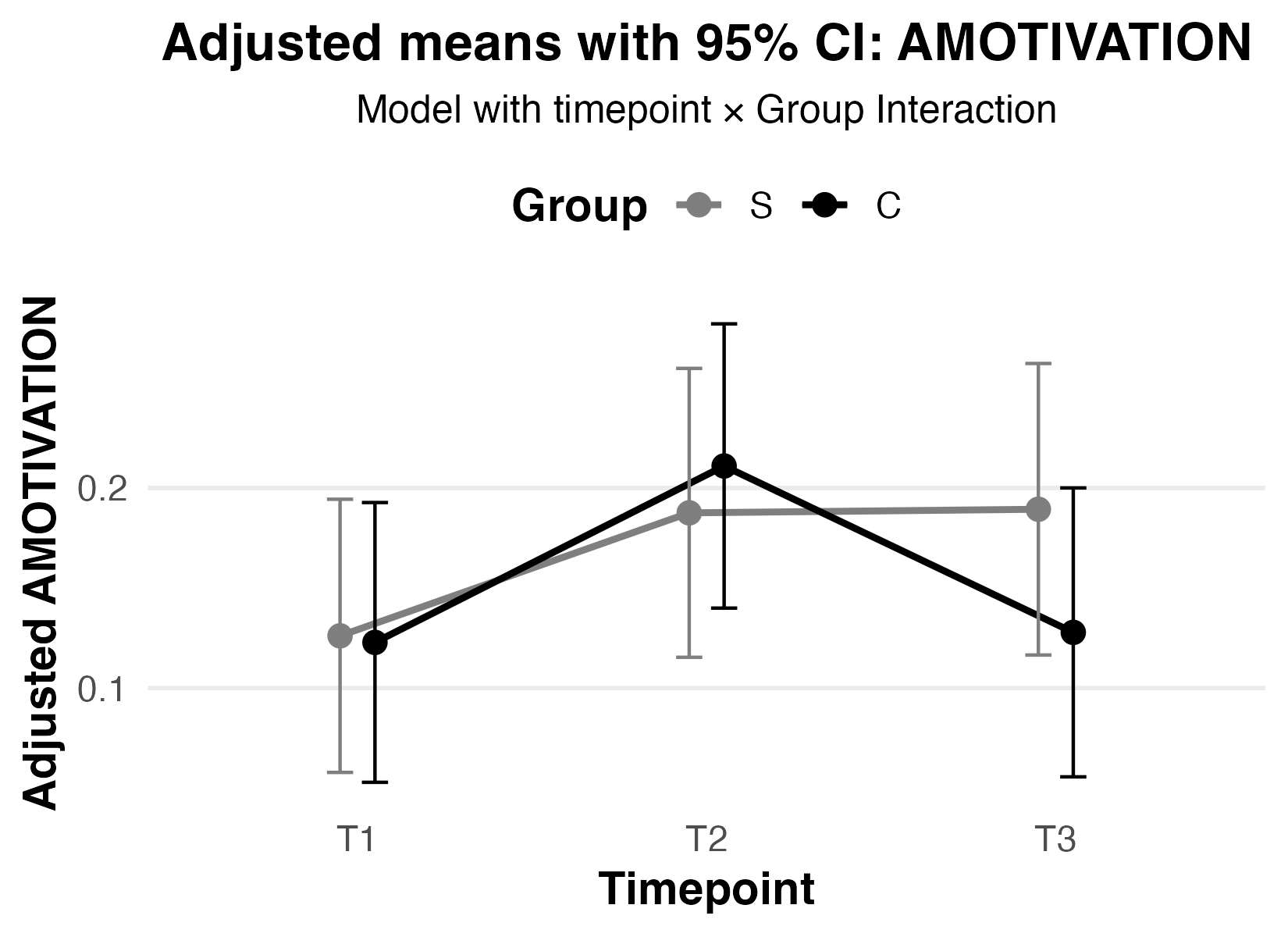


# Outcome: extregulation

## Number of Participants Included: 79

## Distribution of DV at Baseline


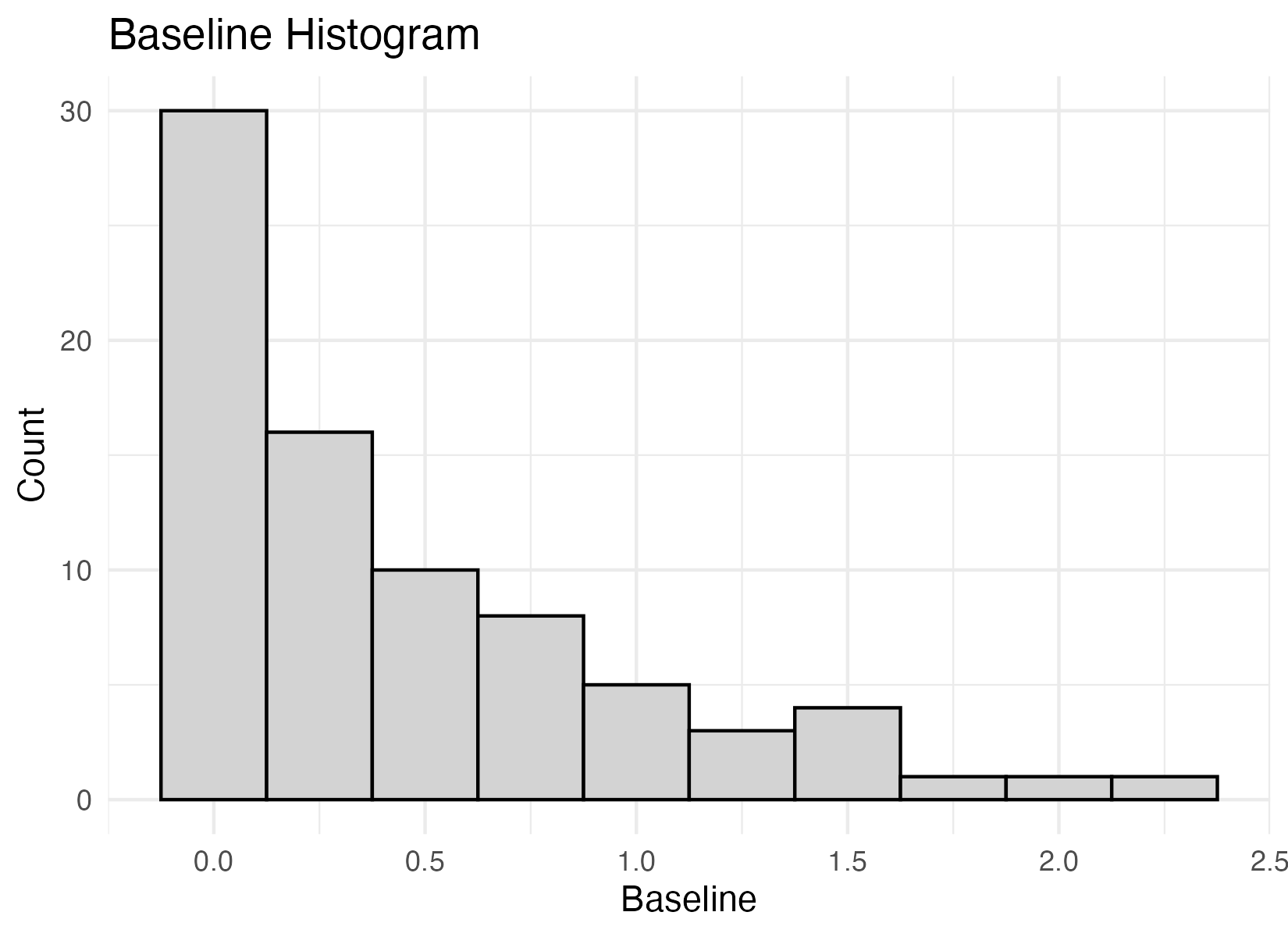


## Fitted vs Residuals


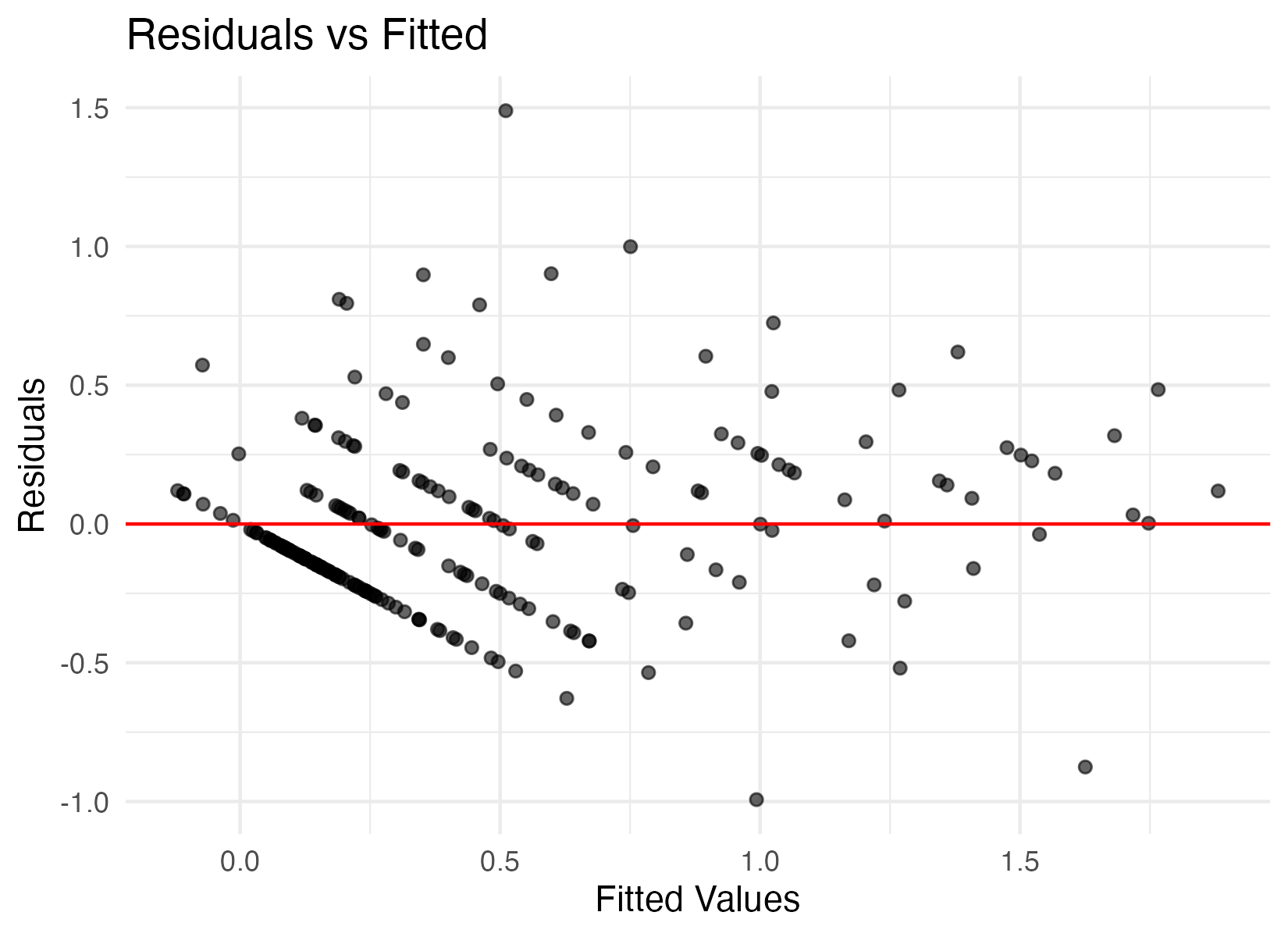


## QQ Plot


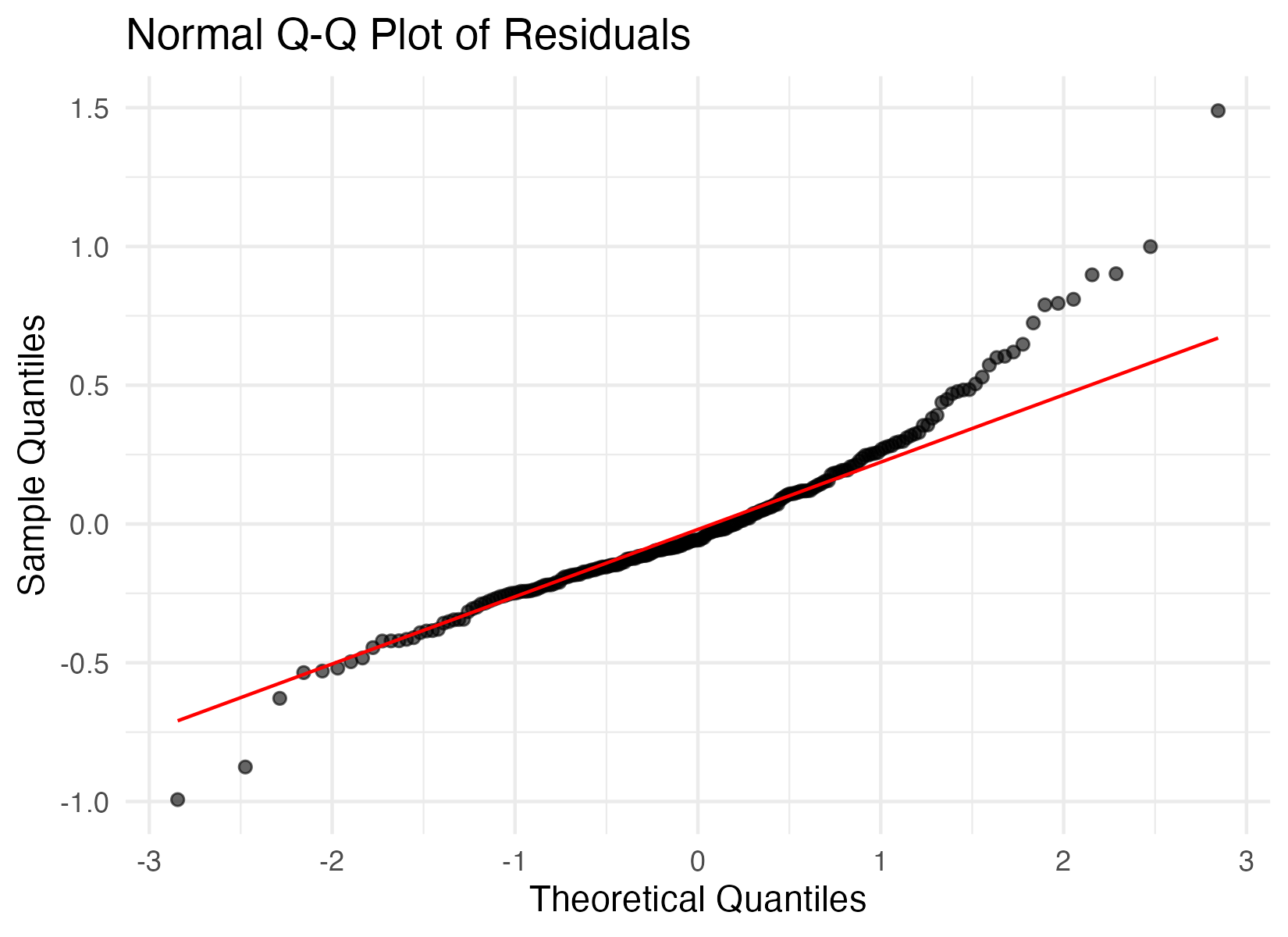


## Within-group change (baseline to follow-up)

| contrast | group | estimate | SE | df | lower.CL | upper.CL | t.ratio | p.value | effect_size |
| --- | --- | --- | --- | --- | --- | --- | --- | --- | --- |
| T2 - T1 | C | 0.066 | 0.077 | 212 | -0.116 | 0.247 | 0.853 | 0.670 | 0.116 |
| T3 - T1 | C | 0.036 | 0.077 | 212 | -0.147 | 0.218 | 0.460 | 0.890 | 0.063 |
| T3 - T2 | C | -0.030 | 0.079 | 212 | -0.216 | 0.156 | -0.379 | 0.924 | -0.053 |
| T2 - T1 | S | 0.115 | 0.078 | 212 | -0.069 | 0.299 | 1.479 | 0.303 | 0.204 |
| T3 - T1 | S | -0.140 | 0.079 | 212 | -0.326 | 0.045 | -1.784 | 0.177 | -0.248 |
| T3 - T2 | S | -0.256 | 0.079 | 212 | -0.442 | -0.069 | -3.235 | 0.004 | -0.453 |

## Between-group difference in change (interaction)

| timepoint_revpairwise | group_revpairwise | estimate | SE | df | lower.CL | upper.CL | t.ratio | p.value | effect_size |
| --- | --- | --- | --- | --- | --- | --- | --- | --- | --- |
| T2 - T1 | S - C | 0.050 | 0.109 | 212 | -0.166 | 0.265 | 0.455 | 0.650 | 0.088 |
| T3 - T1 | S - C | -0.176 | 0.110 | 212 | -0.393 | 0.042 | -1.594 | 0.112 | -0.312 |
| T3 - T2 | S - C | -0.226 | 0.112 | 212 | -0.446 | -0.005 | -2.019 | 0.045 | -0.400 |

## Adjusted Means Over Time (with 95% CI)


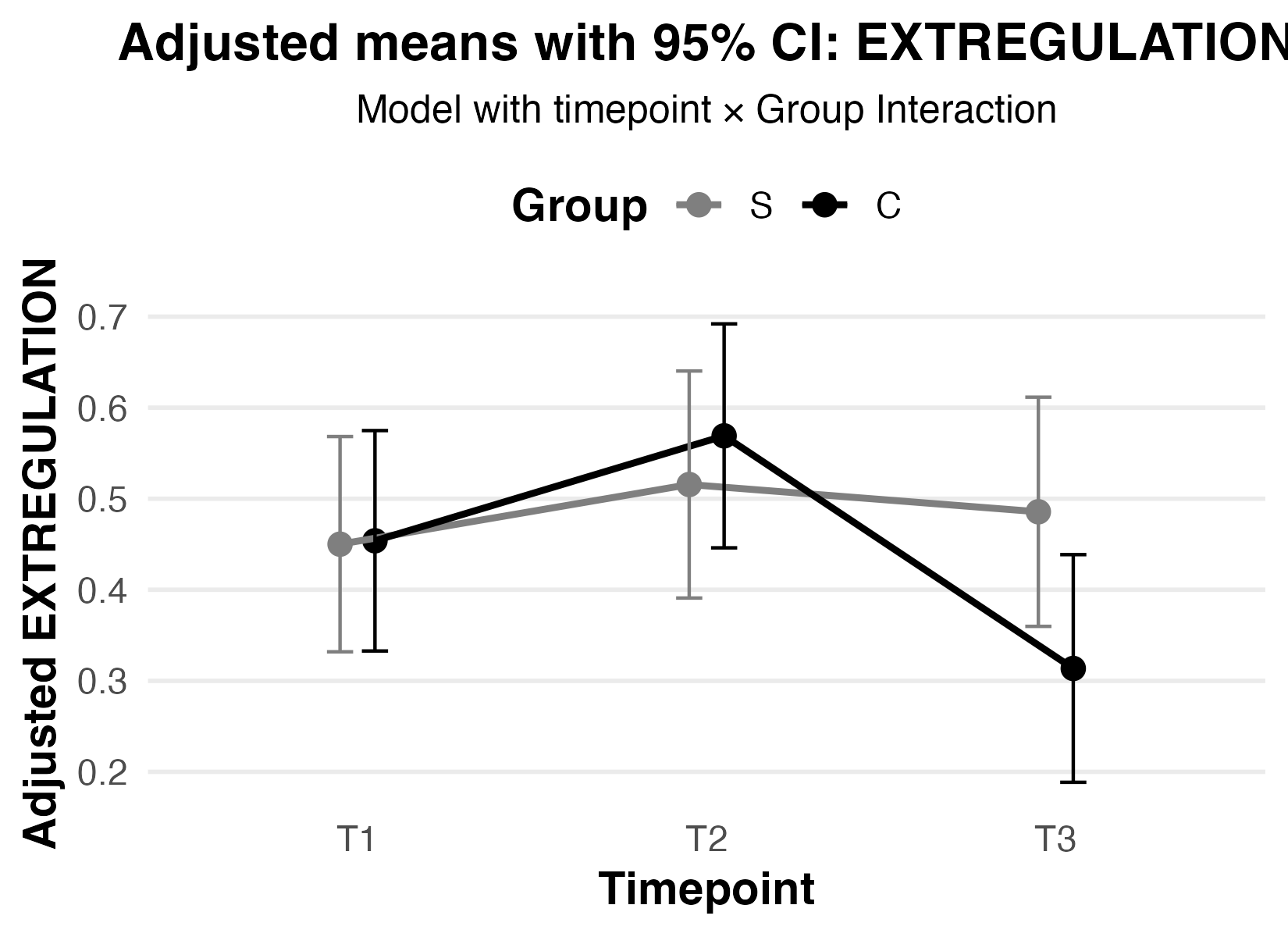


# Outcome: introjregulation

## Number of Participants Included: 79

## Distribution of DV at Baseline


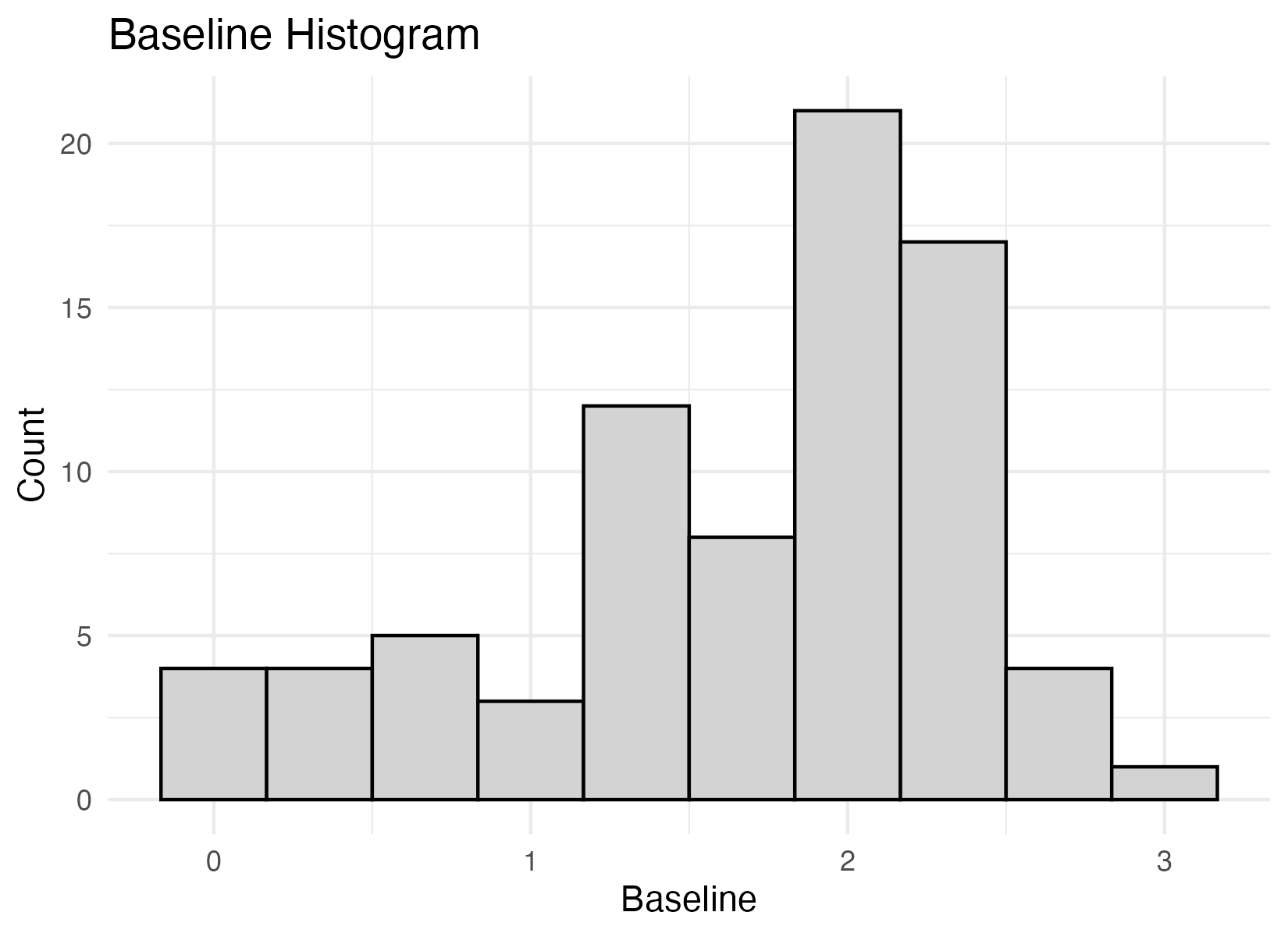


## Fitted vs Residuals


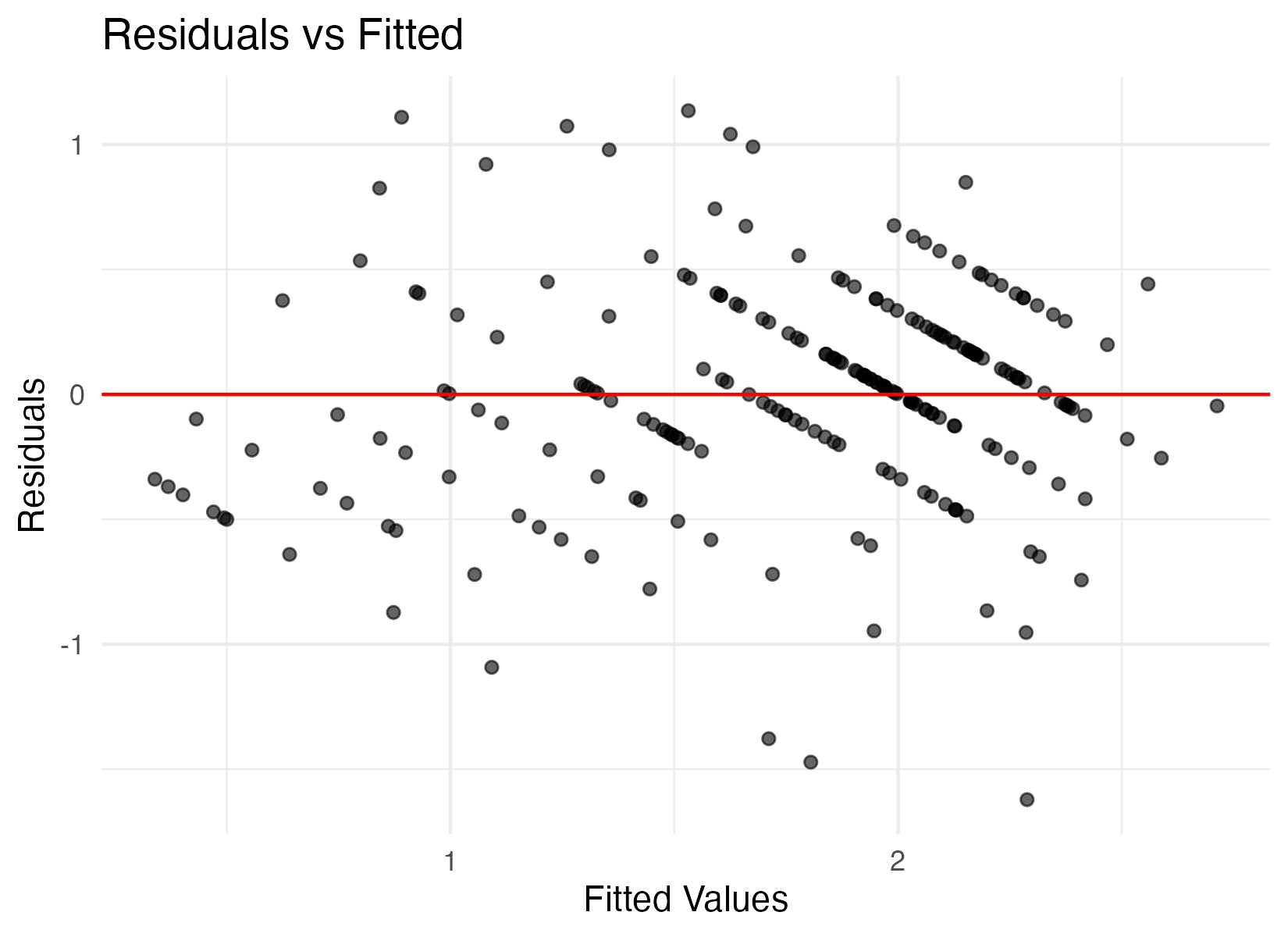


## QQ Plot


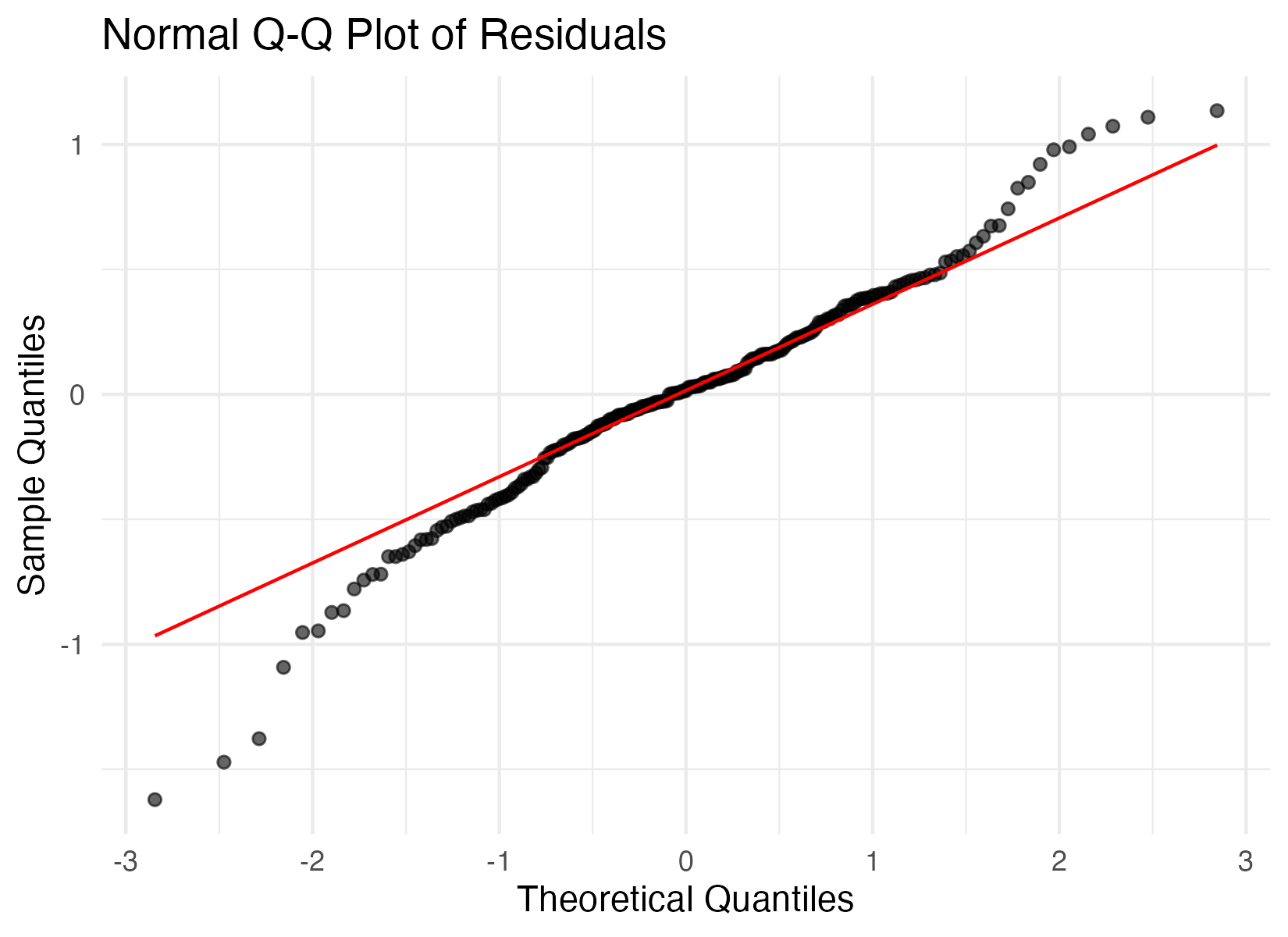


## Within-group change (baseline to follow-up)

| contrast | group | estimate | SE | df | lower.CL | upper.CL | t.ratio | p.value | effect_size |
| --- | --- | --- | --- | --- | --- | --- | --- | --- | --- |
| T2 - T1 | C | 0.201 | 0.104 | 212 | -0.043 | 0.445 | 1.944 | 0.129 | 0.288 |
| T3 - T1 | C | 0.107 | 0.104 | 212 | -0.139 | 0.353 | 1.028 | 0.560 | 0.154 |
| T3 - T2 | C | -0.094 | 0.107 | 212 | -0.346 | 0.158 | -0.881 | 0.653 | -0.135 |
| T2 - T1 | S | 0.154 | 0.105 | 212 | -0.094 | 0.403 | 1.467 | 0.309 | 0.221 |
| T3 - T1 | S | 0.030 | 0.106 | 212 | -0.220 | 0.280 | 0.281 | 0.957 | 0.043 |
| T3 - T2 | S | -0.124 | 0.107 | 212 | -0.376 | 0.127 | -1.167 | 0.474 | -0.178 |

## Between-group difference in change (interaction)

| timepoint_revpairwise | group_revpairwise | estimate | SE | df | lower.CL | upper.CL | t.ratio | p.value | effect_size |
| --- | --- | --- | --- | --- | --- | --- | --- | --- | --- |
| T2 - T1 | S - C | -0.047 | 0.148 | 212 | -0.338 | 0.244 | -0.318 | 0.751 | -0.067 |
| T3 - T1 | S - C | -0.077 | 0.149 | 212 | -0.371 | 0.216 | -0.520 | 0.603 | -0.111 |
| T3 - T2 | S - C | -0.031 | 0.151 | 212 | -0.328 | 0.267 | -0.203 | 0.840 | -0.044 |

## Adjusted Means Over Time (with 95% CI)


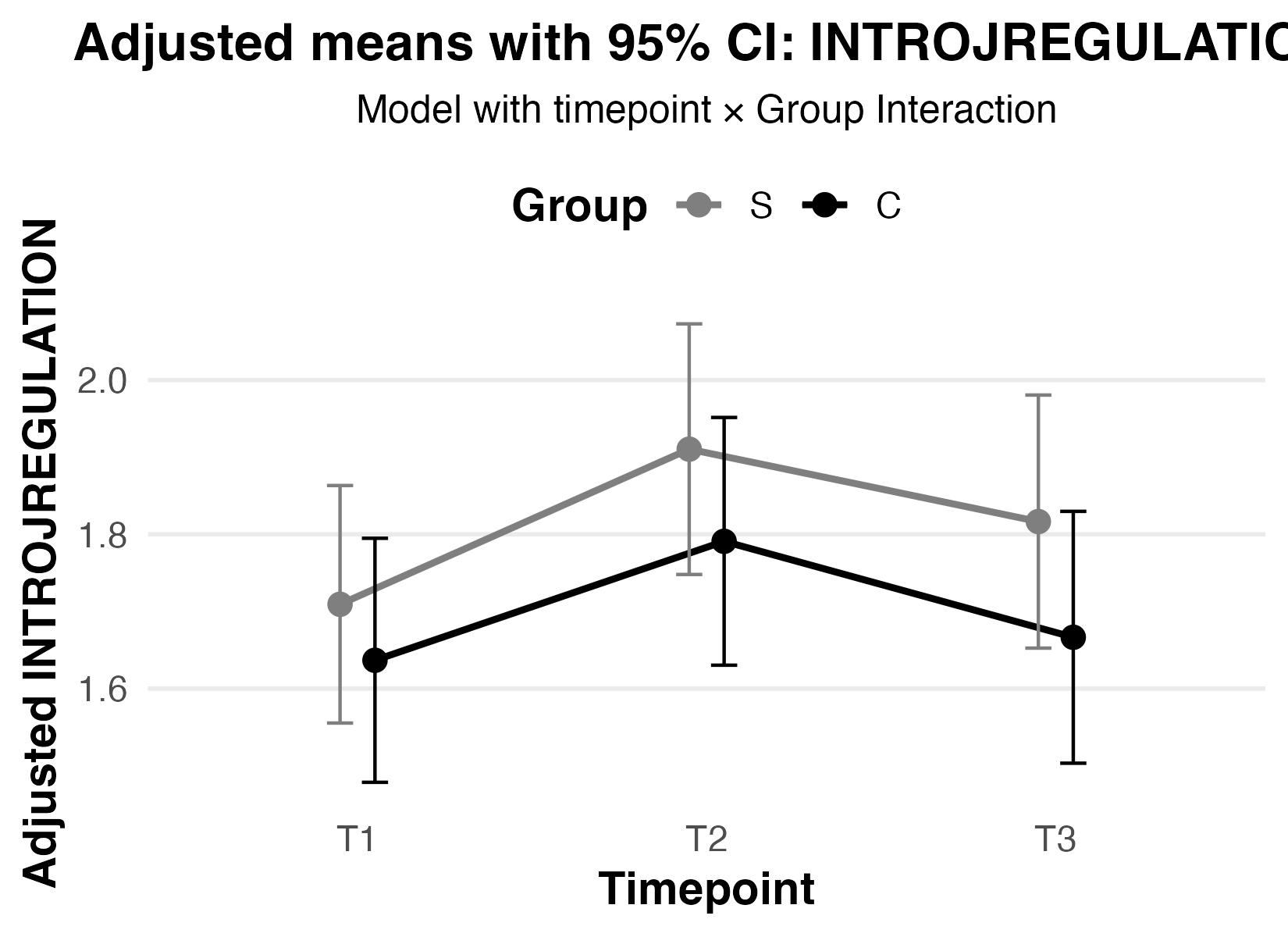


# Outcome: identregulation

## Number of Participants Included: 79

## Distribution of DV at Baseline


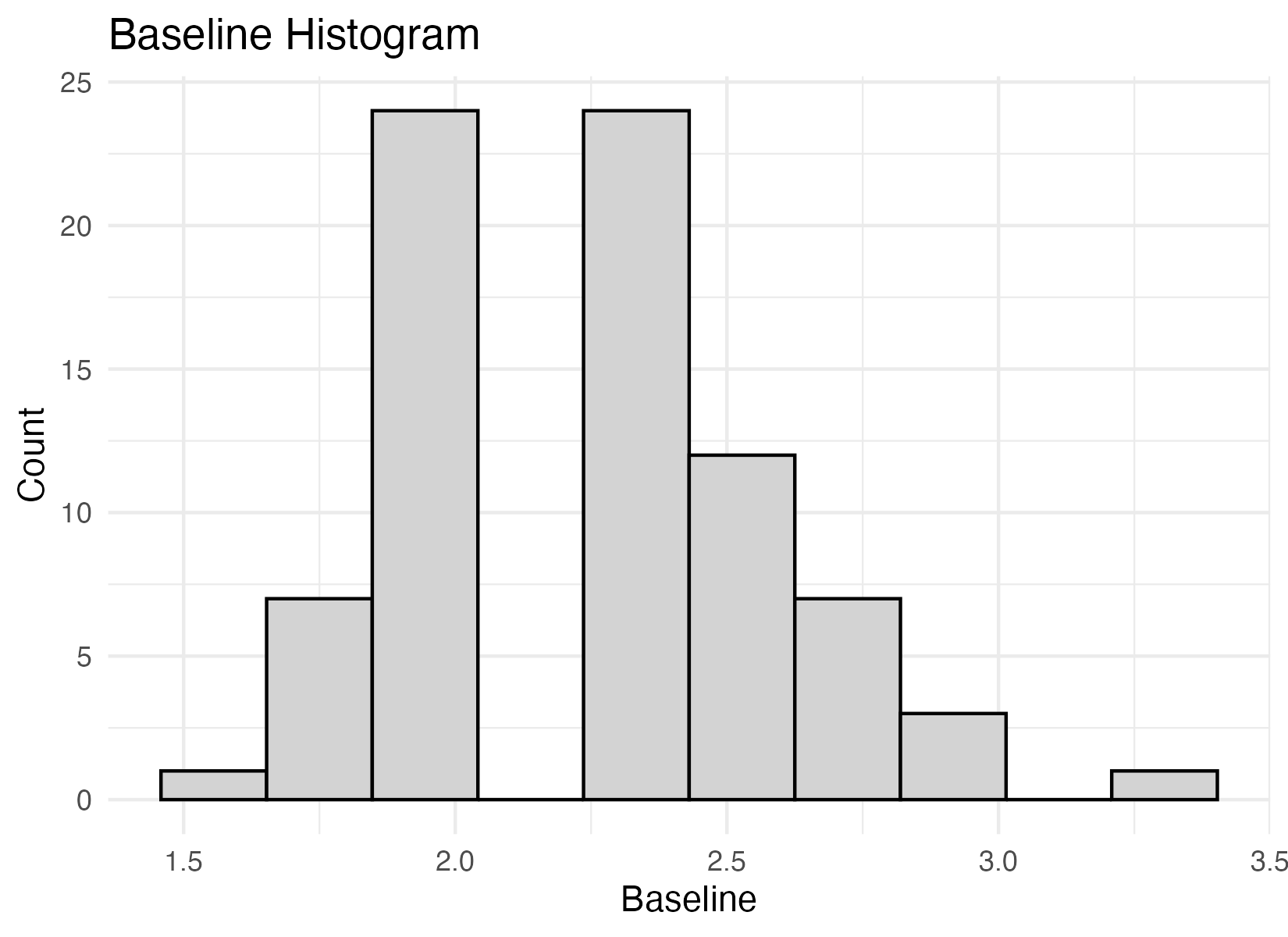


## Fitted vs Residuals


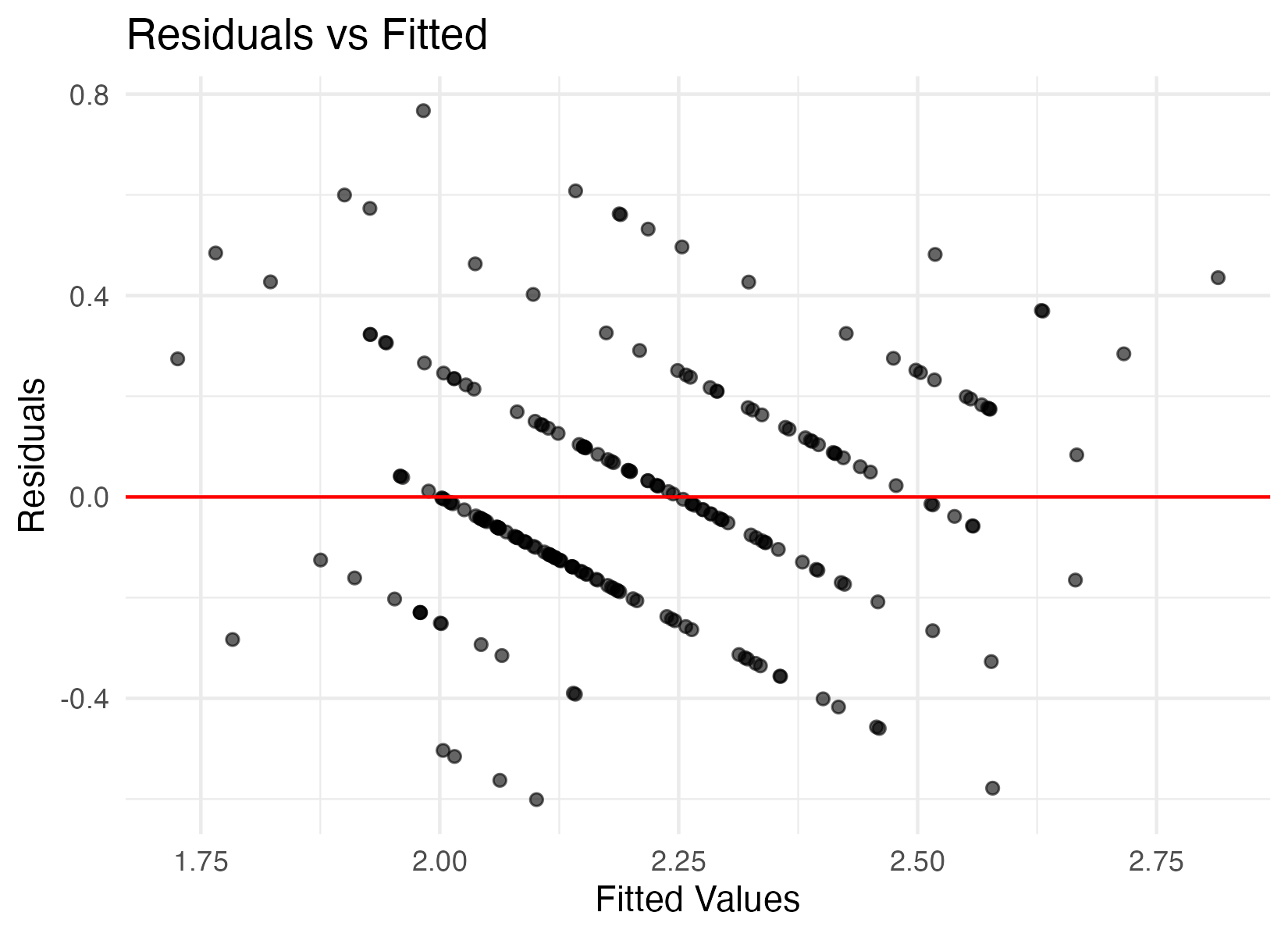


## QQ Plot


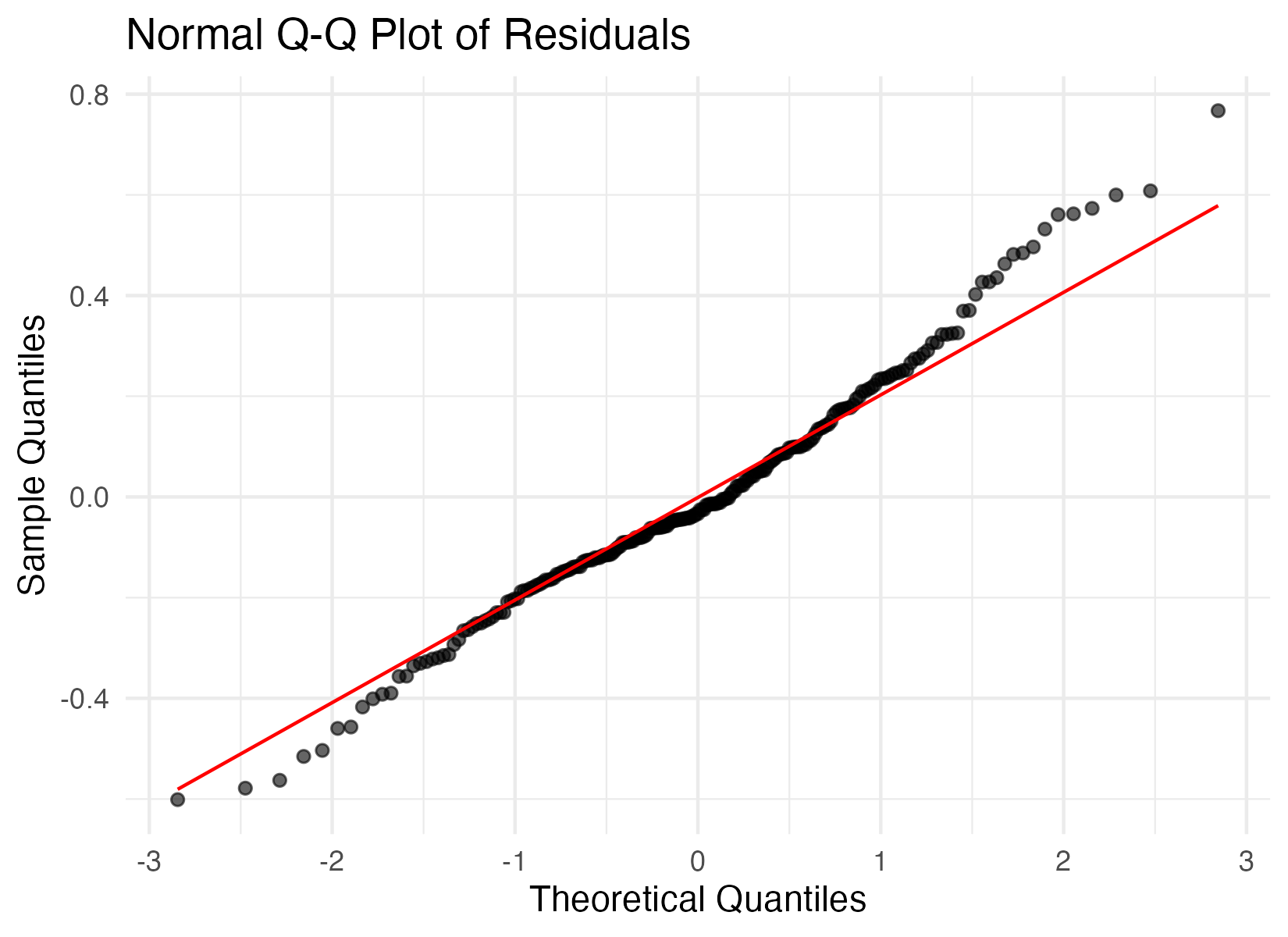


## Within-group change (baseline to follow-up)

| contrast | group | estimate | SE | df | lower.CL | upper.CL | t.ratio | p.value | effect_size |
| --- | --- | --- | --- | --- | --- | --- | --- | --- | --- |
| T2 - T1 | C | -0.018 | 0.053 | 212 | -0.144 | 0.108 | -0.332 | 0.941 | -0.058 |
| T3 - T1 | C | -0.057 | 0.054 | 212 | -0.184 | 0.070 | -1.068 | 0.535 | -0.188 |
| T3 - T2 | C | -0.040 | 0.055 | 212 | -0.170 | 0.090 | -0.722 | 0.751 | -0.130 |
| T2 - T1 | S | 0.036 | 0.054 | 212 | -0.093 | 0.164 | 0.656 | 0.789 | 0.117 |
| T3 - T1 | S | -0.052 | 0.055 | 212 | -0.182 | 0.077 | -0.958 | 0.604 | -0.172 |
| T3 - T2 | S | -0.088 | 0.055 | 212 | -0.218 | 0.042 | -1.599 | 0.248 | -0.289 |

## Between-group difference in change (interaction)

| timepoint_revpairwise | group_revpairwise | estimate | SE | df | lower.CL | upper.CL | t.ratio | p.value | effect_size |
| --- | --- | --- | --- | --- | --- | --- | --- | --- | --- |
| T2 - T1 | S - C | 0.053 | 0.076 | 212 | -0.097 | 0.203 | 0.700 | 0.485 | 0.175 |
| T3 - T1 | S - C | 0.005 | 0.077 | 212 | -0.146 | 0.156 | 0.065 | 0.948 | 0.016 |
| T3 - T2 | S - C | -0.048 | 0.078 | 212 | -0.202 | 0.105 | -0.620 | 0.536 | -0.158 |

## Adjusted Means Over Time (with 95% CI)


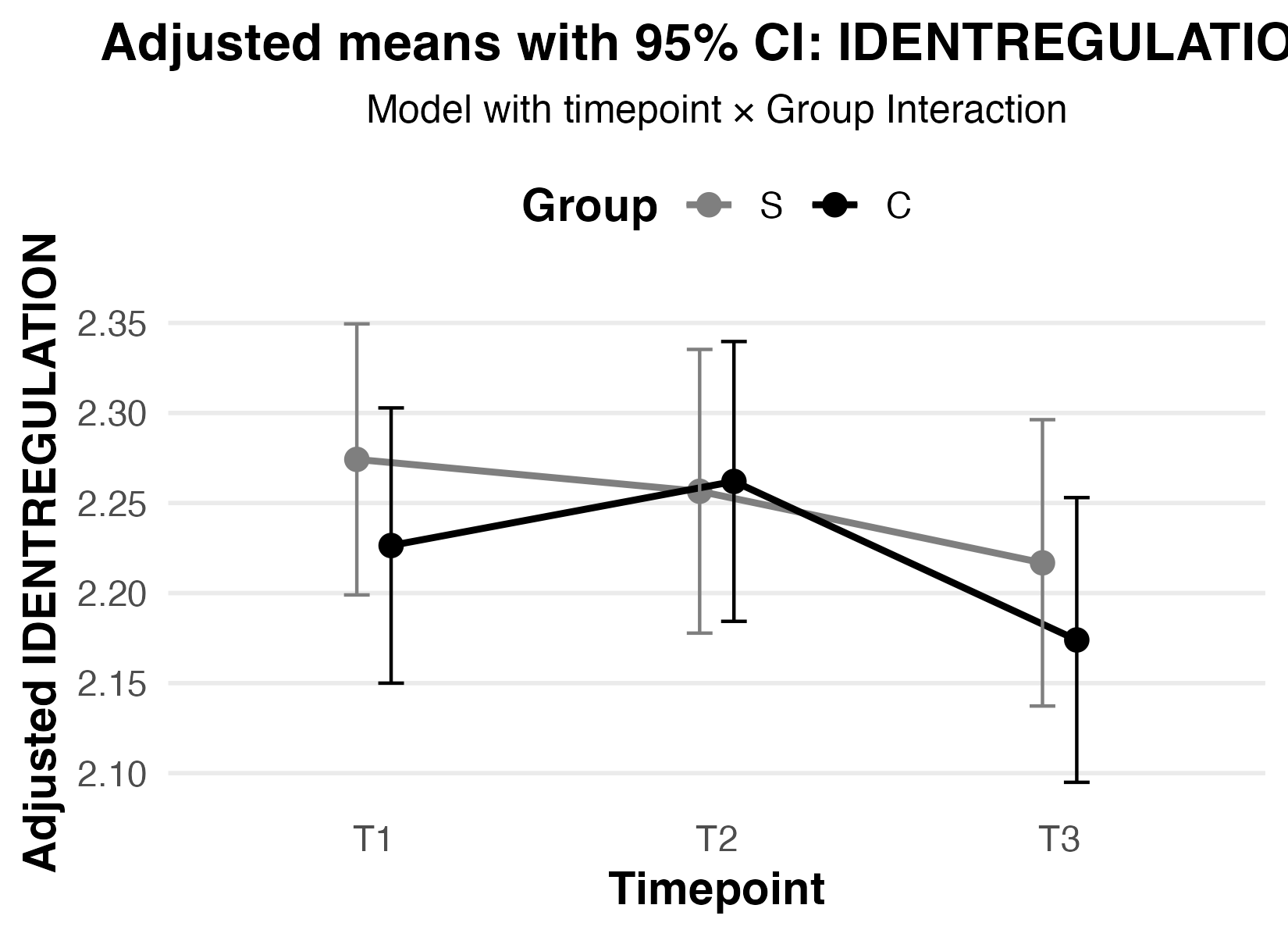


# Outcome: intrinregulation

## Number of Participants Included: 79

## Distribution of DV at Baseline


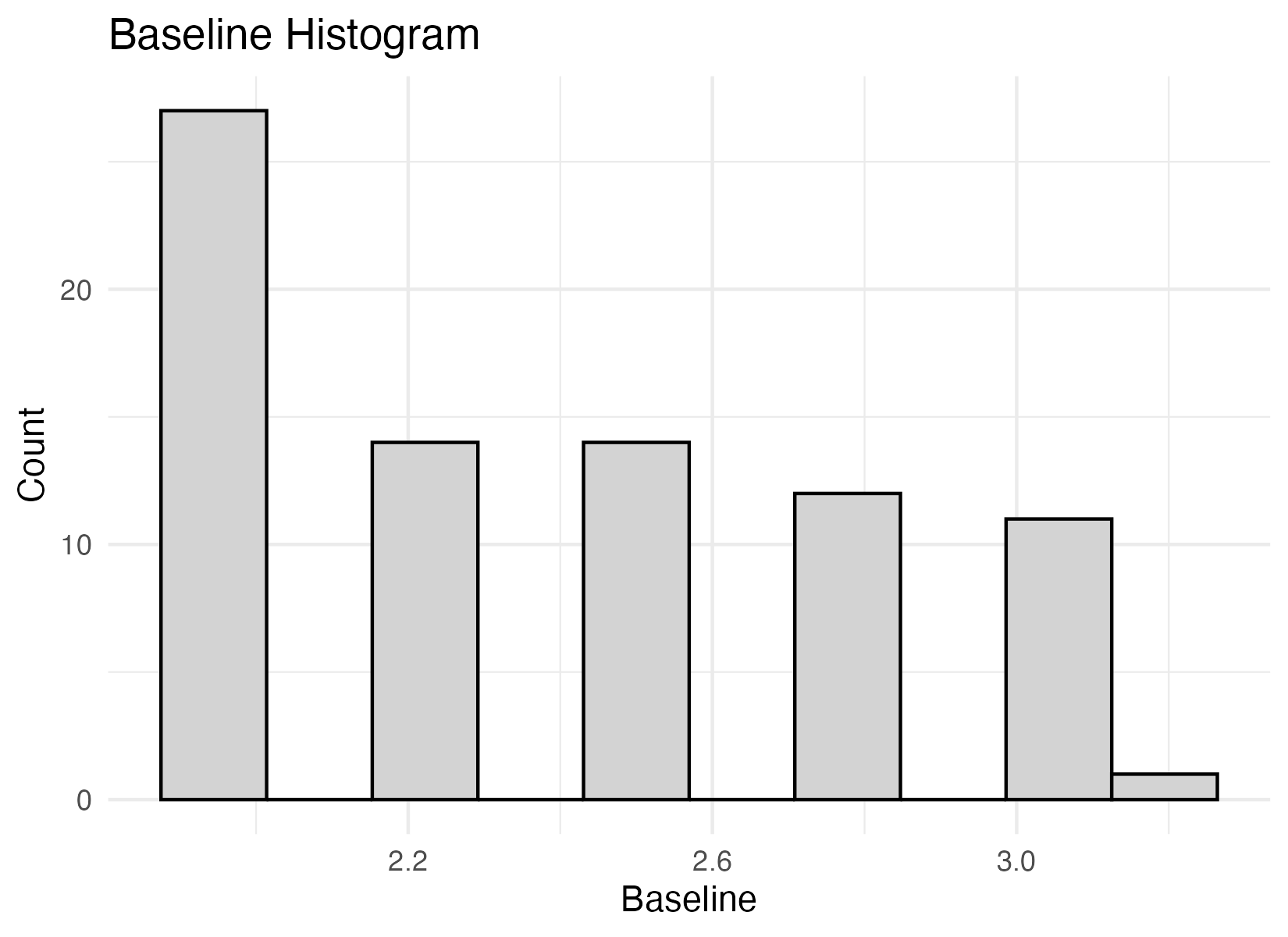


## Fitted vs Residuals


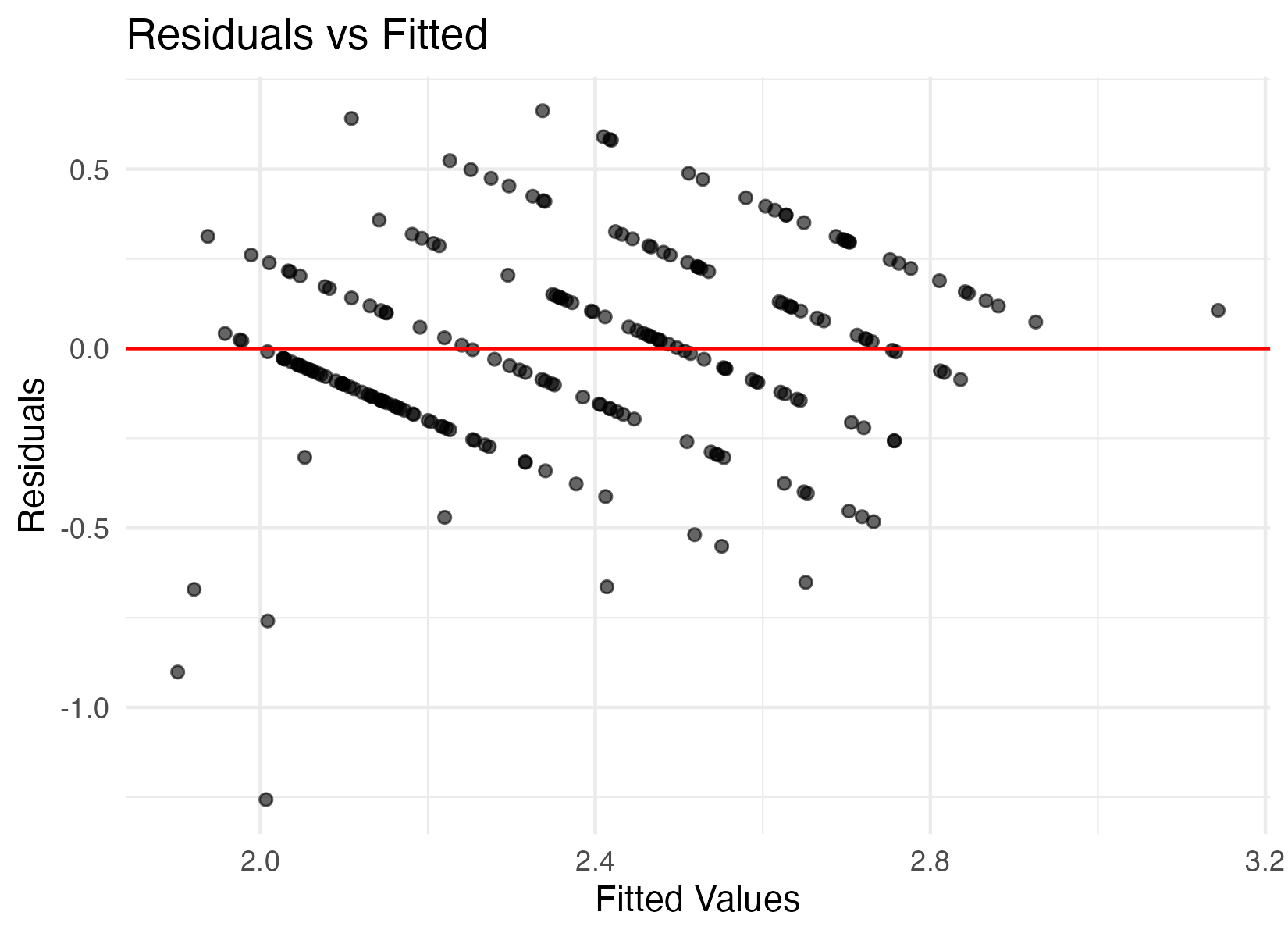


## QQ Plot


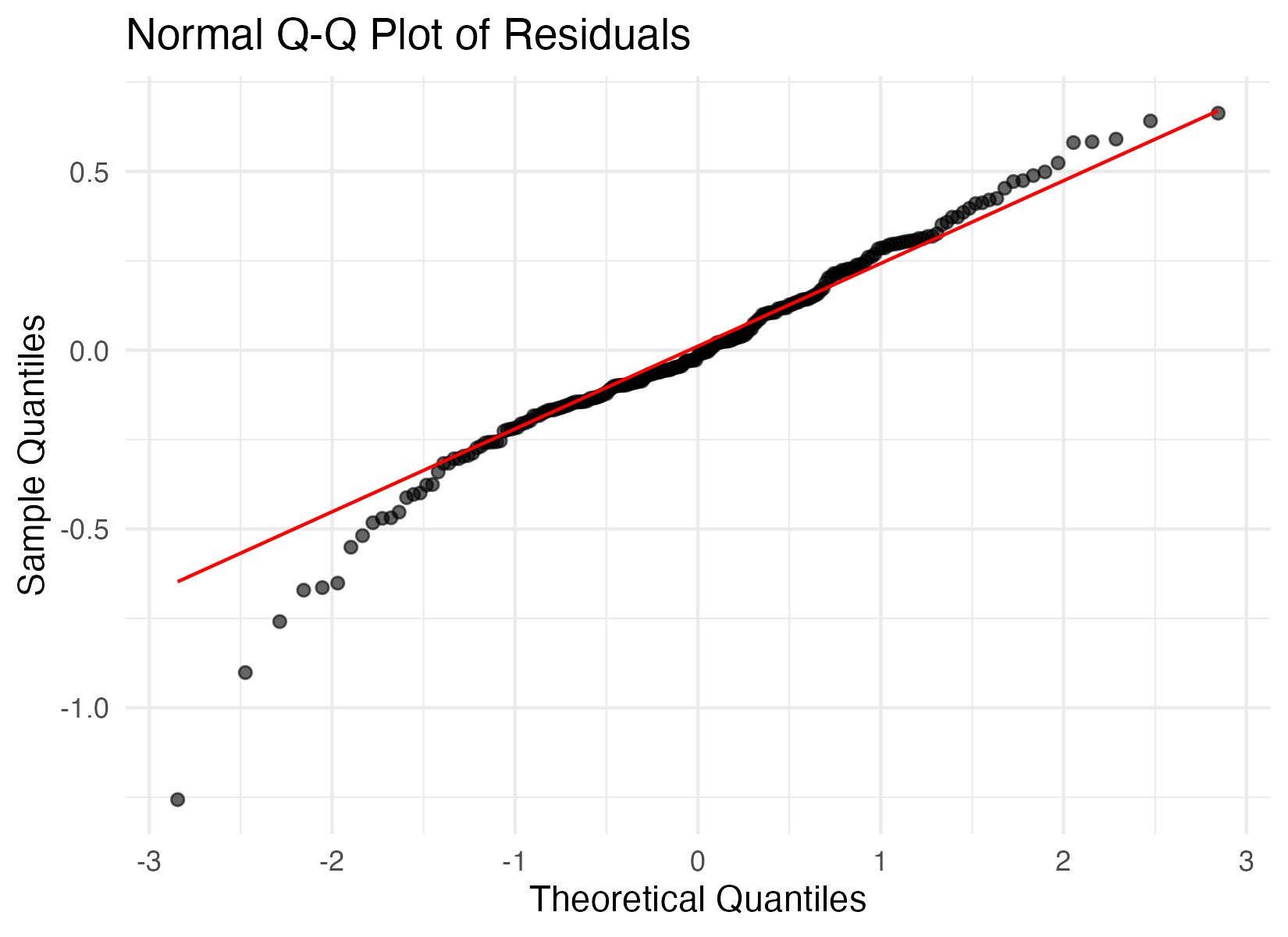


## Within-group change (baseline to follow-up)

| contrast | group | estimate | SE | df | lower.CL | upper.CL | t.ratio | p.value | effect_size |
| --- | --- | --- | --- | --- | --- | --- | --- | --- | --- |
| T2 - T1 | C | -0.090 | 0.066 | 212 | -0.246 | 0.066 | -1.357 | 0.365 | -0.226 |
| T3 - T1 | C | -0.109 | 0.067 | 212 | -0.266 | 0.048 | -1.642 | 0.231 | -0.275 |
| T3 - T2 | C | -0.020 | 0.068 | 212 | -0.180 | 0.141 | -0.289 | 0.955 | -0.049 |
| T2 - T1 | S | 0.069 | 0.067 | 212 | -0.089 | 0.228 | 1.036 | 0.555 | 0.175 |
| T3 - T1 | S | 0.072 | 0.068 | 212 | -0.088 | 0.231 | 1.058 | 0.541 | 0.180 |
| T3 - T2 | S | 0.002 | 0.068 | 212 | -0.158 | 0.162 | 0.031 | 0.999 | 0.005 |

## Between-group difference in change (interaction)

| timepoint_revpairwise | group_revpairwise | estimate | SE | df | lower.CL | upper.CL | t.ratio | p.value | effect_size |
| --- | --- | --- | --- | --- | --- | --- | --- | --- | --- |
| T2 - T1 | S - C | 0.159 | 0.094 | 212 | -0.026 | 0.345 | 1.690 | 0.092 | 0.401 |
| T3 - T1 | S - C | 0.181 | 0.095 | 212 | -0.006 | 0.368 | 1.906 | 0.058 | 0.456 |
| T3 - T2 | S - C | 0.022 | 0.096 | 212 | -0.168 | 0.211 | 0.226 | 0.822 | 0.055 |

## Adjusted Means Over Time (with 95% CI)


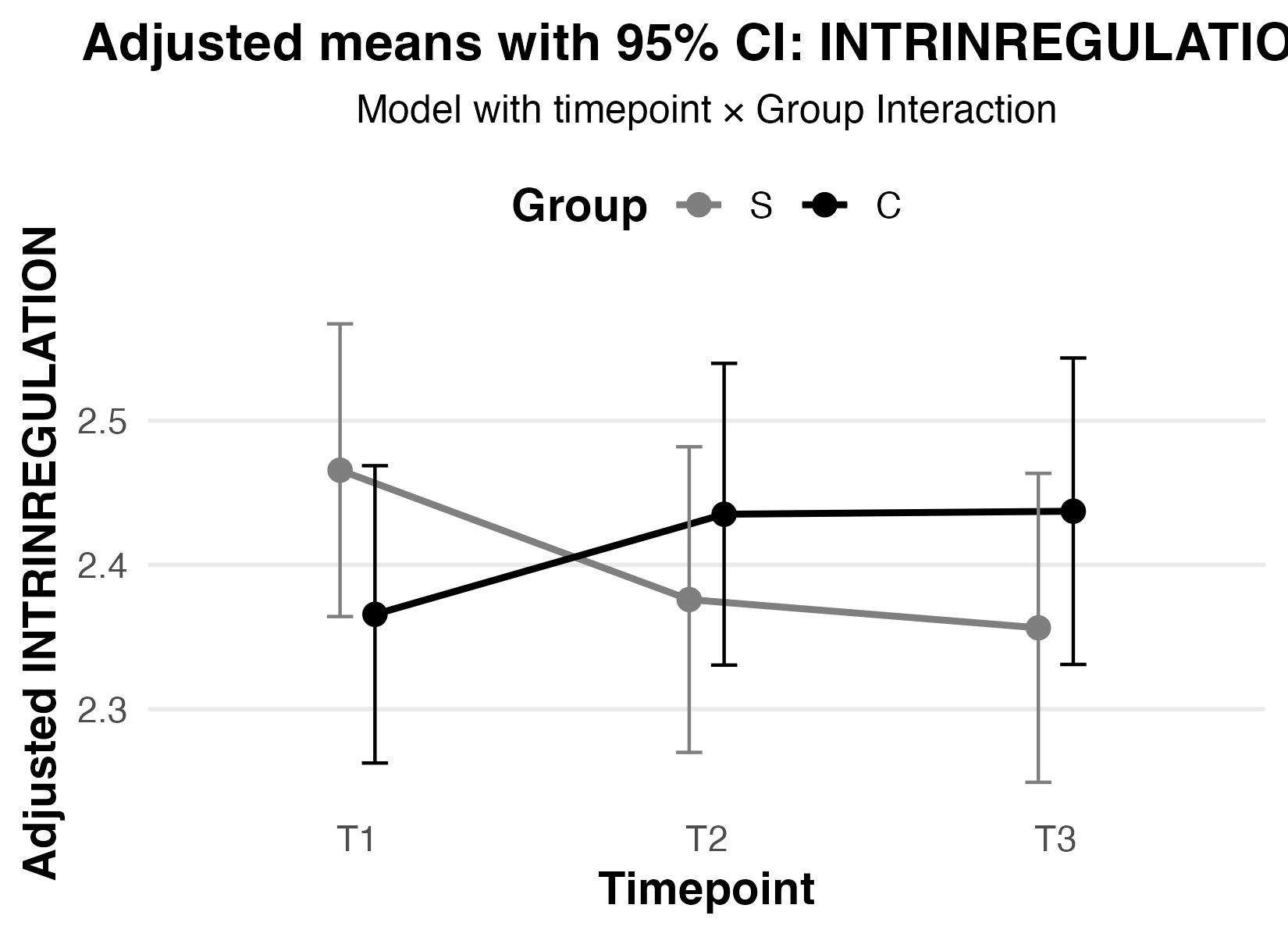

Supplement: Supplementary file 7 — Supporting Information S7 [file EJSC-26-e70211-s005.docx]
