# Supplementary material for: Community‐Based High‐Intensity Multimodal Training: A Mixed‐Method Evaluation of a Randomised Control Trial
Source: Eur J Sport Sci. 2026 Jun 26;26(7):e70211. doi: 10.1002/ejsc.70211 (PMC13309294; doi:10.1002/ejsc.70211)
Supplement: Supplementary file 8 — Supporting Information S8 [file EJSC-26-e70211-s003.docx]

Supplementary Material 8 Code Frequencies for Open-ended Survey Responses

| **Question** | **Code** | **Total Frequency (n)** | **HIMT** | | **ISCT** | |
| --- | --- | --- | --- | --- | --- | --- |
| Timepoint | |  | Post-testing | Follow up | Post-testing | Follow up |
| *Why or why not has your physical activity participation changed in the last 4 weeks compared to prior to participating in this study?* | | |  |  |  |  |
|  | Life logistics | 24 |  | 10 |  | 14 |
|  | Intrinsic factors | 12 |  | 5 |  | 7 |
|  | Physical results/ improvements | 11 |  | 6 |  | 5 |
|  | Study created routine | 11 |  | 5 |  | 6 |
|  | Already engaged (no change) | 8 |  | 7 |  | 1 |
| *What did you enjoy most about the training mode you participated in?* | | |  |  |  |  |
|  | Exercise Prescription Variables | 28 | 8 |  | 20 |  |
|  | Variety | 16 | 10 |  | 6 |  |
|  | Group environment | 12 | 7 |  | 5 |  |
|  | The "feeling" | 11 | 6 |  | 5 |  |
|  | Routine/ consistency | 10 | 4 |  | 6 |  |
|  | Physical/ health Improvement | 9 | 5 |  | 4 |  |
|  | Coaches | 6 | 4 |  | 2 |  |
|  | Challenge | 3 | 3 |  | 0 |  |
|  | Physical Environment | 0 | 0 |  | 0 |  |
| *What did you enjoy the least about the training mode you participated in?* | | |  |  |  |  |
|  | Exercise Prescription Variables | 37 | 16 |  | 21 |  |
|  | Physical discomfort | 20 | 7 |  | 13 |  |
|  | Time commitment | 8 | 4 |  | 4 |  |
|  | Physical environment | 2 | 0 |  | 2 |  |
|  | Motivation | 1 | 1 |  | 0 |  |
| *Why or why not has your confidence in your ability to exercise four times per week for 45 minutes changed since participating in this study?* | | |  |  |  |  |
|  | Accountability | 74 | 15 | 19 | 21 | 19 |
|  | Positive feelings/ enablers | 32 | 12 | 6 | 7 | 7 |
|  | Saw benefits | 24 | 2 | 7 | 7 | 8 |
|  | Barriers | 19 | 5 | 5 | 2 | 7 |
|  | Education | 17 | 4 | 3 | 7 | 3 |
| *Why or why not has your motivation to exercise changed since participating in this study?* | | |  |  |  |  |
|  | Routine | 43 | 10 | 7 | 13 | 13 |
|  | Physical Benefits | 42 | 12 | 12 | 9 | 9 |
|  | Exercise selection/ type | 20 | 8 | 3 | 6 | 3 |
|  | Intrinsic Benefits | 13 | 3 | 3 | 3 | 4 |
|  | Negative psycho-social feelings | 8 | 1 | 3 | 1 | 3 |
|  | Group | 4 | 1 | 1 | 0 | 2 |
| *“Do you have any other feedback for the research team on the training sessions you completed during this study?”* | | |  |  |  |  |
|  | Thankful for the positive experience | 20 |  | 8 |  | 12 |
|  | Felt supported by interventionists | 8 |  | 1 |  | 6 |
|  | Provided constructive feedback | 5 |  | 1 |  | 4 |
|  | Group environment | 2 |  | 1 |  | 1 |
| *“Do you have any other feedback for the research team on your general experiences during this study?”* | | |  |  |  |  |
|  | Thankful for the positive experience | 17 |  | 7 |  | 10 |
|  | Program was well organised | 6 |  | 4 |  | 2 |
|  | Felt supported by interventionists | 3 |  | 2 |  | 1 |
|  | Provided constructive feedback | 3 |  | 2 |  | 1 |

HIMT, *High-Intensity Multimodal Training,* ISCT, *Inter-Session Concurrent Training,* n, *number*
